# Human Activity Impacts on Macrofungal Diversity: A Case Study of Grazing in Subtropical Forests

**DOI:** 10.3390/jof11100749

**Published:** 2025-10-20

**Authors:** Kun L. Yang, Xunan Xiong, Zejia Luo, Yanqun Huang, Rong Huang, Huajie Chen, Jia Y. Lin, Zhu L. Yang, Guang-Mei Li, Xiaorong Jia

**Affiliations:** 1 College of Forestry and Landscape Architecture, South China Agricultural University, Guangzhou 510642, China; mugoture@gmail.com (K.L.Y.);; 2 College of Plant Protection, South China Agricultural University, Guangzhou 510642, China; mycofloscula@gmail.com; 3 Yunnan Key Laboratory for Fungal Diversity and Green Development, Kunming Institute of Botany, Chinese Academy of Sciences, Kunming 650201, China; fungi@mail.kib.ac.cn (Z.L.Y.);

**Keywords:** macrofungal diversity, alpha-diversity, beta-diversity, mushrooms, human activities, grazing, subtropical forests, plantations, taxonomy, new taxa

## Abstract

Concerns about potential negative impacts of human activity on macrofungal diversity are spreading globally, yet research on this topic remains scarce. This study focuses on forest grazing (silvopasture), a popular economic practice whose impacts on macrofungal diversity are underexplored. Through investigation and comparison of macrofungal diversity and selected environmental factors in three types of subtropical forests (secondary mixed forests, dense-tree plantations and sparse-tree plantations) before and after two years of grazing at an intensity of 10 goats per hectare in South China, three key findings emerged: (1) Macrofungal alpha-diversity increased significantly after grazing, associated with an increase in large plant remains and a decrease in litterfall thickness; (2) dominance was monopolized by few taxa before grazing but became more balanced among a number of taxa after grazing; and (3) dominance of endemic taxa decreased in two of the three types of forests after grazing. Such findings suggest that grazing may create additional niches through foraging, trampling and excretion by livestock and thus recruit diverse macrofungi but may also lead to homogenization of fungal florae across regions and thus result in recessive beta-diversity loss. As this study heavily relies on taxonomy, allied updates for ambiguous taxa recognized in analyses are additionally proposed.

## 1. Introduction

In a traditional concept adopted in this study, macrofungi refer to true fungi and slime molds visible to the naked eye, encompassing a group of organisms with similar morphological and ecological characteristics but polyphyletic evolutionary origins [1,2]. They play crucial ecological roles in nature by decomposing organic debris and regulating the growth of plants and animals (e.g., the common saprotroph *Candelolepiota sinica* [3]). They directly or indirectly provide humans with foods, medicines, materials and energy, holding significant economic value (e.g., the renowned edible fungi *Termitomyces* spp. [4]). They also cause food spoilage, structural damage and diseases in humans and livestock, posing various adverse effects on human production and daily life (e.g., the shiitake-farm-contaminating fungus *Pycnoporus sinoruber* [5]). These complex interactions have drawn considerable attention to these organisms, with both the desire to preserve their diversity in natural environments to conserve bioresources and concern to prevent harmful ones from hindering development. A key foundation for achieving such goals is to elucidate the patterns of macrofungal diversity in response to environmental changes. However, certain aspects of this topic, particularly the impacts of human activities on macrofungal diversity, remain underexplored.

With the popularization of the concept “The Sixth Extinction”, human activities have been widely recognized as a major force influencing today’s global biodiversity [6]. Concerns about potential negative impacts of human activities on macrofungal diversity in natural environments have emerged worldwide, primarily focusing on mushroom harvesting. For instance, the government of Bhutan has implemented policies to limit the number of people harvesting *Ophiocordyceps sinensis* [7], some national forests in the United States restrict the weight of mushrooms that can be collected by the public [8], and four species of edible or medical macrofungi are legally protected in Mainland China, requiring permits for lawful harvesting [9]. However, these restrictions have sparked controversy in some regions due to insufficient scientific evidence. Although mushroom yield declines have been reported in some areas with harvesting traditions, related ecological studies generally suggest that mushroom harvesting has no significant impact on macrofungal diversity, with factors like climate change at various scales and ground trampling during harvesting more likely responsible [10,11,12,13,14,15,16]. The discrepancy between research findings and perceived negatives of some activities on macrofungal diversity implies that the regarding patterns need to be quantitatively reassessed.

Other types of activities have not garnered as much societal attention as mushroom harvesting, but some have attracted the interest of fungal ecologists. Ohtonen & Markkola (1989) [17] and Fellner (1990) [18] suggested that the decline of some ectomycorrhizal macrofungi, such as *Russula mustelina*, is associated with air pollution. Baar & Kuyper (1993) [19] found that mowing and removal of litter and humus layers exhibited positive impacts on species richness and yield of ectomycorrhizal macrofungi. Zervakis & Venturella (2007) [20] summarized the literature indicating that the application of some chemical fertilizers in forests would negatively affect the yield of many ectomycorrhizal and some saprotrophic macrofungi. Varenius et al. (2016) [21] studied impacts of tree harvesting on ectomycorrhizal fungal communities, which involved many macrofungi, and noticed community changes but no significant differences in species richness and common species data among natural forests, regenerated forests that experienced shelterwood cutting and planted forests that experienced clear cutting. Gómez-Hernández et al. (2021) [22] worked on impacts of urbanization on macrofungal communities and found that species richness decreased with increasing urbanization, primarily associated with microclimate changes. Furthermore, grazing is also a widespread activity that may affect macrofungal diversity, but few studies have addressed this point.

Given advantages like high productivity and broad incomes, livestock grazing in forests (silvopasture) has become an increasingly popular economic practice [23,24,25,26]. The livestock released into forests can easily alter the natural environment through processes like foraging, trampling and excretion [27], potentially affecting macrofungal communities. Based on published research, the response patterns of macrofungal diversity to forest grazing remain unknown, and therefore, we study this topic using the following approach: (1) investigating macrofungal diversity (characterization indices of species diversity, evenness and richness; dominant orders, species, trophic types, attachment types, sporocarp types, geographic components and edibility types) and selected potentially relevant environmental factors (litterfall thickness and abundance of large plant remains) before and after two years of grazing at an intensity of 10 goats per hectare in three common types of subtropical forests in South China (secondary mixed forests, dense-tree plantations and sparse-tree plantations); (2) analyzing differences in the above data before and after grazing; and (3) identifying the relationships between environmental factors and macrofungal diversity.

## 2. Materials and Methods

### 2.1. Experimental Site

**Figure 1:**
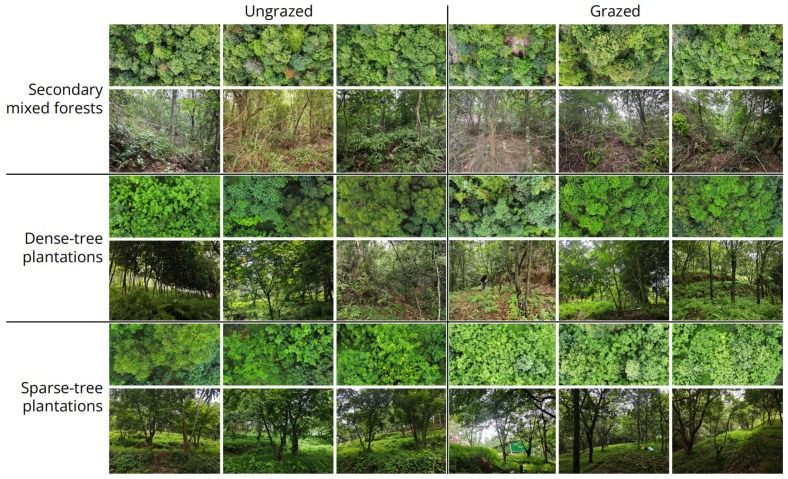
Local canopy and understory conditions of the 18 quadrats in six plots investigated in this study.

The experimental site was located in the hilly and mountainous area surrounding Qianfeng Village, Huiyang District, Huizhou City, Guangdong Province, in South China (22°58′27″ N, 114°36′09″ E–22°58′42″ N, 114°36′25″ E). This area features three distinct types of forests (Figure 1): (1) secondary mixed forests, exhibiting a well-developed canopy dominated by *Schima superba*, *Liquidambar formosana*, *Cratoxylum cochinchinense*, *Triadica cochinchinensis*, *Manglietia fordiana*, *Acacia auriculiformis*, *Ilex asprella*, *Toxicodendron succedaneum*, *Pinus massoniana*, *Camphora officinarum* and *Heptapleurum heptaphyllum*, some shrubs and few herbs dominated by *Dicranopteris pedata*, *Coniogramme japonica* and *Paspalum orbiculare*; (2) dense-tree plantations (generally >5 trees per 100 m^2^), exhibiting a well-developed canopy dominated by *Archidendron clypearia*, *Schima superba* and *Eucalyptus* spp., few shrubs and some herbs dominated by *Mikania micrantha*, *Lygodium microphyllum* and *Clerodendrum cyrtophyllum*; and (3) sparse-tree plantations (generally ≤5 trees per 100 m^2^), exhibiting a well-developed canopy dominated by *Archidendron clypearia* and *Magnolia grandiflora* and abundant herbs dominated by *Pteris cretica*, *Microstegium vimineum*, *Ageratum conyzoides*, *Lolium perenne* and *Stylosanthes* sp., without any shrubs.

### 2.2. Experimental Design

#### 2.2.1. Plot and Quadrat Establishment

Within each forest type, two areas were designated: one subjected to grazing at an intensity of 10 goats per hectare for two years, and one left untreated as a control. This resulted in six plot types (grazed secondary mixed forests, ungrazed secondary mixed forests, grazed dense-tree plantations, ungrazed dense-tree plantations, grazed sparse-tree plantations, and ungrazed sparse-tree plantations; Figure 1). Within each plot, three 400 m^2^ quadrats were established, each further divided into four 100 m^2^ subquadrats.

#### 2.2.2. Control of Extraneous Variables

Prior to grazing intervention, grazed and ungrazed quadrats exhibited similar environmental conditions, ensuring that observed impacts could be attributed to grazing activity itself rather than pre-existing environmental differences. To standardize fruiting conditions (e.g., temperature and humidity), the field investigation was conducted from 9 July to 2 August 2025. During this period, daily temperatures consistently ranged between 27 and 35 °C. Each investigation was conducted on the third day after a sufficient rainfall event (≥25 mm/day). Since the investigation process itself causes disturbance to the community (and thus repeated investigations would obtain data unrelated to grazing activity), each quadrat was surveyed only once, at a pace of 5 s/m^2^.

#### 2.2.3. Record of Variables of Interest

In the field, subquadrats served as minimum units for recording observed macrofungal species identified via macroscopic morphology, along with number of individuals, trophic types, attachment types and sporocarp types (Table 1). In densely vegetated areas, measures like clearing weeds, turning over litterfall and breaking up large plant remains were employed to thoroughly uncover hidden species. For species unable to be identified in the field, samples of sporocarps were collected and transported to the laboratory for microscopic and molecular identification (see Section 2.2.5). When necessary, taxonomic updates and revisions were applied to ensure accurate use of taxon concepts (see Section 2.2.5). For taxon complexes we were unable to resolve, morphological taxon concepts were applied. Classifications of the taxonomic order, geographic component and edibility types of each species were determined based on recent studies of the corresponding group.

As the primary ecological role of macrofungi is decomposer, litterfall thickness and abundance of large plant remains were considered as potential factors affecting macrofungal diversity. Such variables were also recorded at the subquadrat level along with fungal data (Table 1). Litterfall thickness was measured at the center of each subquadrat with three sublayers as minimum units, while abundance of large plant remains was assessed for the entire subquadrat and classified into four levels. During the plot establishment phase, we found no significant change in canopy density between grazed and ungrazed plots (Figure 1). Therefore, light-related variables were not considered.

#### 2.2.4. Data Analyses

The following three indices were selected to characterize macrofungal diversity: (1) Simpson’s index of diversity *Div* = 1 − Σ(*n_i_*/*N*)^2^ ∈ [0, 1], where *n_i_* was the individual number of each species and *N* the total individual numbers of all species, reflecting the probability of two individuals randomly selected in community belonging to the same species, with higher values as greater diversity [28]; (2) Pielou’s index of evenness *Eve* = −Σ[(*n_i_*/*N*) × ln(*n_i_*/*N*)]/ln(*S*) ∈ [0, 1], where *S* was the total number of species and *n_i_*, *N* same as abovementioned, reflecting the individual distribution uniformity of different species, with higher values as greater evenness [29]; and (3) Margalef’s index of richness *Ric* = (*S* − 1)/ln(*N*) ∈ [0, +∞), reflecting species richness per unit number of individuals, with higher values showing greater richness [30].

Due to the nested structure of subquadrats within quadrats, data from subquadrats were not entirely independent. Considering this, we applied the Mann–Whitney *U* test to determine whether differences in variables of interest between grazed and ungrazed quadrats were significant, with a two-sided test at a significance level of *α* = 0.05 [31]. The nonparametric effect size measure Cliff’s delta *δ* = (2*U*)/(*n*_1_*n*_2_) − 1 ∈ [−1, 1] was additionally applied to assess the direction and magnitude of effects, where *U* was the Mann–Whitney *U* statistic and *n_1_*, *n_2_* the sample sizes of two data groups [32]. The effects were interpreted as negligible by |*δ*| < 0.147, minor by 0.147 ≤ |*δ*| < 0.33, moderate by 0.33 ≤ |*δ*| < 0.474 and large by |*δ*| ≥ 0.474 [32].

Dominance index *Dom_j_* = {[(*q_j_*/*Q*)/Σ(*q_i_*/*Q*)] + (*n_j_*/*N*)}/2 ∈ [0, 100%], where *q_i_* was the number of subquadrats in which each species occurred, *q_j_* the number of subquadrats in which the concerned group occurred, *Q* the total number of subquadrats, *n_j_* the individual number of the concerned group and *N* same as abovementioned, reflects the relative frequency and density of the concerned group, with higher values showing greater dominance. This index was used to evaluate changes in dominant trophic types, attachment types, sporocarp types, geographical components, taxonomic orders and edibility types before and after grazing.

Principal component analysis (PCA) was employed to assess correlations between macrofungal diversity indices and environmental factors [33].

#### 2.2.5. Taxonomic Studies

To ensure the accuracy of taxon classification or definition in above analyses, we conducted taxonomic studies on some directly or indirectly related taxa with both morphological and molecular approaches.

For morphological studies, macroscopic characteristics were described based on field notes and photos of the collections, while microscopic characteristics were described from fresh, air-dried or silica-gel-dried collections after being sectioned and mounted in water, water solution of 1% Congo red, water solution of 5% KOH, lactic acid solution of 2% cotton blue or Melzer’s reagent (water solution of chloral hydrate, potassium iodide and iodine), generally following the terminology in Clémençon et al. (2012) [34] and Vellinga et al. (2018) [35]. Key colors were described following Yang (2024) [36]. Spore measurements (length, width and length/width ratio (*Q*)) followed the notation {*a*/*b*/*c*} (*d*) *e*–*f* (*g*) [*h* ± *i*, *j*], where *e*–*f* represents ≥90% of the measured values, *d* the minimum extreme value, *g* the maximum extreme value, *h* the average value, *i* the sample standard deviation and *j* the mode, measured from *a* spores of *b* sporocarps in *c* collections. Sections were studied using an MSD105 stereomicroscope (SM) (Murzider (Dongguan) Science and Technology Co., Dongguan, China) at a magnification of up to ×80, an MSD105 light microscope (LM) (Murzider (Dongguan) Science and Technology Co., Dongguan, China) at a magnification of up to ×1000 or a ZEISS Sigma 300 scanning electron microscope (SEM) (Carl Zeiss Co., Shanghai, China) at a magnification of up to ×20,000 and an extra high tension of 7000 V after coated with platinum.

For molecular studies, genomic DNA was extracted from tiny sporocarp fragments of air-dried or silica-gel-dried collections by using an Ezup Column Fungi Genomic DNA Purification Kit (Sangon Biotech Co., Shanghai, China). Loci for phylogenetic analyses were selected following the recent usage in certain groups, including the nuclear internal transcribed spacer region (ITS), the nuclear large subunit rDNA (nrLSU), the RNA polymerase II largest subunit gene (*rpb1*), the RNA polymerase II second largest subunit gene (*rpb2*), the translation elongation factor 1 alpha gene (*tef-1α*), the beta tubulin gene (*β-tub*) and the ATP synthase subunit 6 gene (*atp6*), amplified with the primer pairs and specific PCR settings listed in Table 2. All reactions were proceeded with a pre-denaturation at 94 °C for 3 min and a final elongation at 72 °C for 8 min. PCR products were purified and sequenced by Sangon Biotech Co. Raw sequences were checked and trimmed with Chromas v2.6.6 [37] and assembled with AliView v1.28 [38]. Assembled sequences were deposited in GenBank [39] with the accession numbers shown in Table 3. In phylogenetic analyses, samples of ingroup and outgroup were specifically selected for different studied taxa, with consideration on recently published studies of megaphylogeny for the corresponding groups. DNA sequences were aligned using MAFFT v7.450 [40] or MUSCLE v3.8.425 [41] and manually trimmed with AliView v1.28 [38] when necessary. Gaps were treated as missing data. Introns were removed. Alignments of different loci were concatenated by PhyloSuite v1.2.3 [42,43], and the final alignments for phylogenies presented in this article are attached in Alignments S1–S6. RaxmlGUI v2.0.10 [44] was used to determine the best-fit substitution model for the concatenated alignment under the Akaike information criterion (AIC) and to perform a maximum likelihood (ML) analysis employing the best-fit substitution model with 1000 rapid ML bootstrap replicates. Nodes receiving a maximum likelihood bootstrap of over 50% (MLB ≥ 50%) were considered significantly supported. Phylogenetic trees were visualized with FigTree v1.4.0 [45] and iTOL [46].

Holotypes and epitypes proposed in this study were deposited in the Herbarium of Cryptogams in Kunming Institute of Botany of Chinese Academy of Sciences (KUN-HKAS). Isotypes, isoepitypes and the rest were deposited in Kun L. Yang’s private herbarium (HTBM).

## 3. Results

### 3.1. Changes of Macrofungal Diversity After Grazing

**Figure 2:**
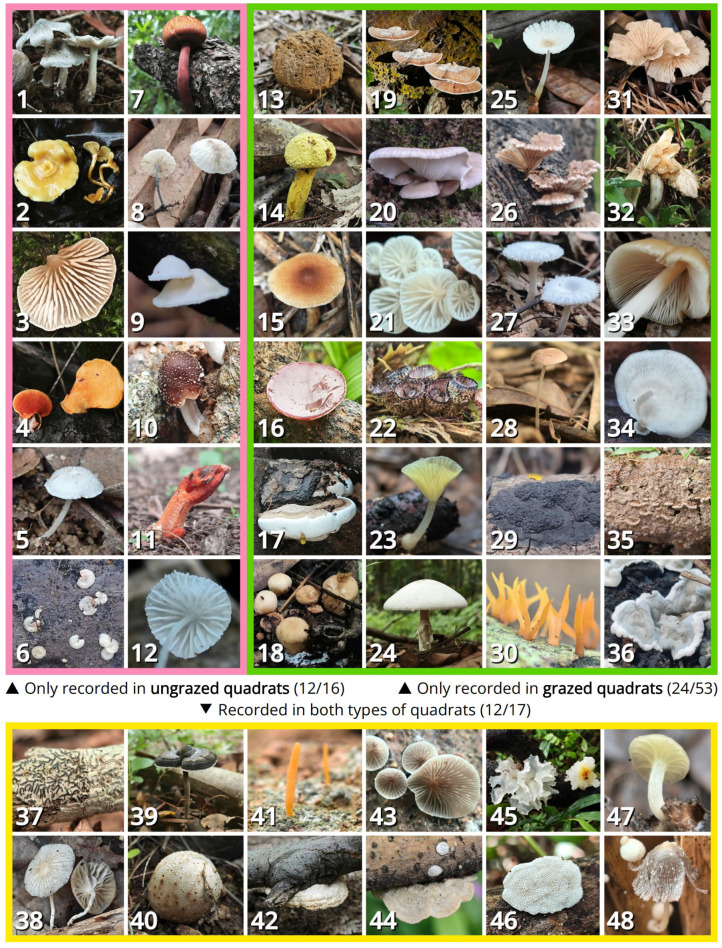
Representative macrofungal species unique to ungrazed quadrats (1–12, showing 12 of 16), unique to grazed quadrats (13–36, showing 24 of 53), or shared in both types of quadrats (37–48, showing 12 of 17): (1) *Termitomyces boluoshanensis*; (2) *Tricholomopsis flava*; (3) *Clitopilus hobsonii*; (4) *Pycnoporus sinoruber*; (5) *Micropsalliota pseudoarginea*; (6) *Hyalorbilia* sp.; (7) *Boletellus aurocontextus*; (8) *Tetrapyrgos parvispora*; (9) *Chaetocalathus galeatus*; (10) *Candolleomyces rubrobrunneus*; (11) *Lysurus mokusin*; (12) *Amparoina heteracantha*; (13) *Pisolithus albus*; (14) *Pulveroboletus icterinus*; (15) *Tricholomopsis rubroaurantiaca*; (16) *Auricularia cornea*; (17) *Ganoderma gibbosum*; (18) *Vascellum curtisii*; (19) *Daedalea atypa*; (20) *Neonothopanus nambi*; (21) *Paramarasmius palmivorus*; (22) *Cyathus striatus*; (23) *Gerronema kuruvense*; (24) *Agaricus praeclarefibrillosus*; (25) *Pluteus griseodiscus*; (26) *Schizophyllum commune*; (27) *Clitopilus crispus*; (28) *Micropsalliota globocystis*; (29) *Annulohypoxylon nitens*; (30) *Calocera sinensis*; (31) *Collybiopsis indocta*; (32) *Entoloma omiense*; (33) *E. luteum*; (34) *Favolus acervatus*; (35) *Trichaptum abietinum*; (36) *Kretzschmaria iranica*; (37) *Graphis scripta*; (38) *Stygiomarasmius scandens*; (39) *Sanguinoderma rugosum*; (40) *Scleroderma yunnanense*; (41) *Sulzbacheromyces sinensis*; (42) *Neofomitella guangxiensis*; (43) *Resupinatus applicatus*; (44) *Trametes hirsuta*; (45) *Tremella fuciformis*; (46) *Truncospora ochroleuca*; (47) *Gerronema microcarpum*; (48) *Coprinopsis urticicola*.

Grazed quadrats recorded 70 species, while ungrazed quadrats recorded 33 species. A total of 86 species were recorded, with 17 shared in both grazed and ungrazed quadrats, 53 unique in grazed quadrats and 16 unique in ungrazed quadrats (Figure 2).

**Figure 3:**
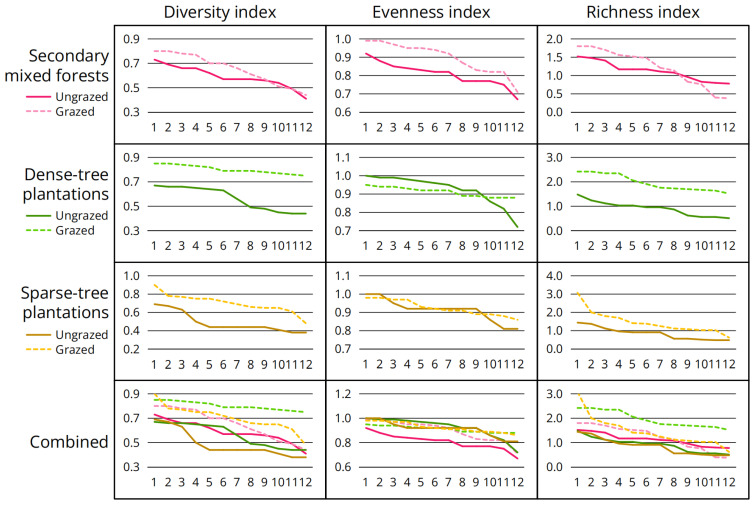
Differences in characterization indices of macrofungal diversity before and after grazing. Twelve quadrats were surveyed for each plot type, sorted in descending order from left to right based on index values.

Analysis of diversity indices (Table 4; Figure 3) revealed two patterns: (1) diversity and richness indices generally increased after grazing, with a significant increase in dense-tree plantations and sparse-tree plantations and a minor to moderate increase in secondary mixed forests; and (2) changes of evenness index varied by forest types, with a significant increase in secondary mixed forests, a moderate decrease in dense-tree plantations and almost no change in sparse-tree plantations.

Analysis of dominant species (Table 5) revealed two patterns: (1) all forest types possessed a dominant species with over 40% dominance before grazing, while after grazing, the dominance recorded for any species was ≤33.12%; and (2) changes in the top three dominant species after grazing varied by forest types, with no changes in secondary mixed forests, the third dominant species changed in sparse-tree plantations and only the first dominant species remained unchanged in dense-tree plantations, suggesting that grazing had the greatest impact on dominant species composition in dense-tree plantations followed by sparse-tree plantations and lastly secondary mixed forests.

Analysis of dominant orders (Table 6) yielded results generally consistent with those for dominant species: (1) all forest types were dominated by Ostropales before grazing, while after grazing, the dominance of the top three orders generally became more balanced; note that secondary mixed forests exhibited greater resistance to this effect compared to two plantation types; and (2) Agaricales and Polyporales demonstrated stronger adaptability in grazed environments, frequently displacing the original dominance of Ostropales.

Analysis of dominant trophic types (Table 7) revealed the following pattern: Before grazing, all forest types were more or less dominated by alga symbionts, while after grazing, wood saprotrophs significantly displaced alga symbionts, and soil saprotrophs mostly increased as well. Secondary mixed forests again exhibited greater resistance to this effect compared to the two plantation types.

Analysis of dominant attachment types (Table 8) revealed the following pattern: Across all forest types, the dominance ratios of wood-inhabiting and soil-inhabiting fungi generally remained stable before and after grazing, with wood-inhabiting fungi being dominated and soil-inhabiting fungi as the secondary components, suggesting that grazing had no significant impact on the attachment types of macrofungal communities.

Analysis of dominant sporocarp types (Table 9) yielded results generally consistent with those for dominant species, orders and trophic types: (1) before grazing, all forest types were monopolistically dominated by smaller, often lichenized ascomycetes, while after grazing, the dominance of the top three sporocarp types generally became more balanced; and (2) larger, usually saprotrophic agaricoid fungi and polyporoid fungi demonstrated stronger adaptability in grazed environments, frequently displacing the original dominance of ascomycetes.

Analysis of dominant geographical components (Table 10) revealed two patterns: (1) before grazing, all forest types contained only cosmopolitan and pantropical components, while after grazing, other components were introduced; and (2) grazing increased the dominance of cosmopolitan components but decreased the dominance of pantropical components in secondary mixed forests and sparse-tree plantations, while it decreased the dominance of cosmopolitan components but increased the dominance of pantropical components in dense-tree plantations.

Analysis of dominant edibility types (Table 11) revealed that commercial macrofungi generally increased after grazing. Secondary mixed forests again exhibited greater resistance to this effect compared to the two plantation tyspes.

### 3.2. Changes of Environmental Factors of Interest After Grazing

**Figure 4:**
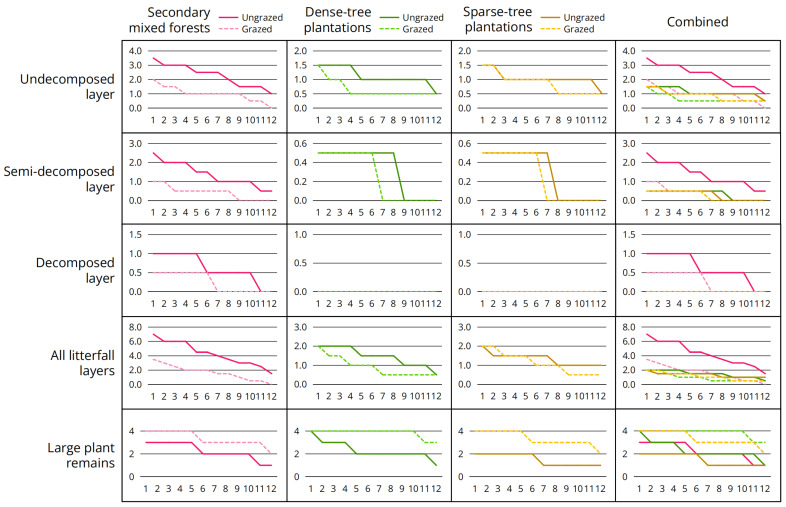
Differences in environmental factors of interest before and after grazing. Twelve quadrats were surveyed for each plot type, sorted in descending order from left to right based on index values.

Analysis of environmental factors of interest (Table 12; Figure 4) revealed two patterns: (1) Litterfall thickness decreased significantly after grazing, with the most pronounced decline observed in undecomposed layer; (2) Abundance of large plant remains increased significantly after grazing.

### 3.3. Correlation Between Macrofungal Diversity and Environmental Factors of Interest

**Figure 5:**
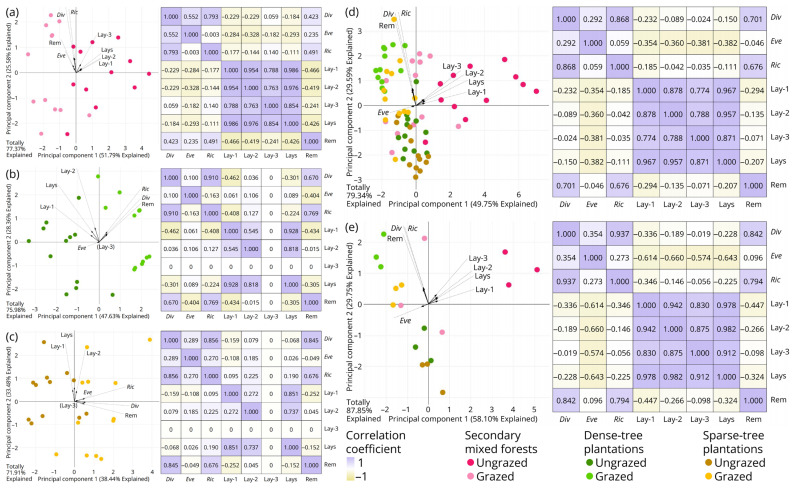
Principal component analyses (PCA) for characterization indices of macrofungal diversity and environmental factors of interest before and after grazing. (**a**) Secondary mixed forests, with 24 subquadrats as minimum units; (**b**) dense-tree plantations, with 24 subquadrats as minimum units; (**c**) sparse-tree plantations, with 24 subquadrats as minimum units; (**d**) three forest types integrated, with 72 subquadrats as minimum units; (**e**) three forest types integrated, with 18 quadrats as minimum units. Abbreviations: (*Div*) Simpson’s index of diversity; (*Eve*) Pielou’s index of evenness; (*Ric*) Margalef’s index of richness; (Lay-1) thickness of undecomposed layer; (Lay-2) thickness of semi-decomposed layer; (Lay-3) thickness of decomposed layer—note that this is excluded from the PCA calculation of (**b**,**c**) where its variance is zero; (Lays) thickness of all litterfall layers; (Rem) abundance of large plant remains.

Principal component analyses for characterization indices of macrofungal diversity and environmental factor variables (Figure 5) revealed two patterns: (1) diversity, evenness and richness indices were slightly negatively correlated with litterfall thickness; and (2) diversity and richness indices were significantly positively correlated with abundance of large plant remains, while evenness index showed no significant correlation with this factor.

### 3.4. Taxonomic Updates and Revisions

**Figure 6:**
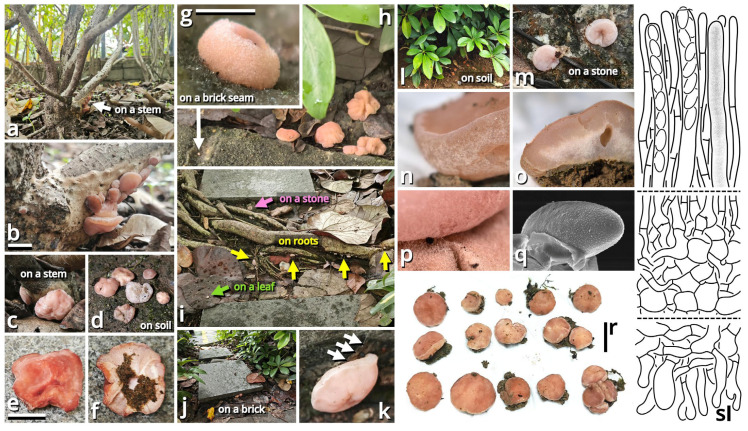
*Purpureodiscus masticophilus* (photos by Kun L. Yang & Jia Y. Lin; drawings by Kun L. Yang from HKAS150755 (holotype)). (**a**–**r**) Ascomata and their habitats. (**a**,**b**) HTBM2877, growing on a stem of *Hibiscus rosa-sinensis*; (**c**) HTBM2878, growing on a stem of *Hibiscus rosa-sinensis*, note the whitish to yellowish subiculum (see also (**p**)); (**d**) HTBM2447, growing on soil, without a subiculum (see also o); (**e**,**f**) HTBM2874 placed on ground, originally growing on a stem of *Hibiscus rosa-sinensis*; (**g**,**h**) HTBM2489, a group of ascomata found on soil covered by a thick litter layer, with an isolated, tiny ascoma situated on a near brick seam; note that an indistinct subiculum surrounding the ascoma can be recognized; (**i**) HTBM2491, a group of ascomata occurring in various microhabitats, including a stone (see also (**m**)), a leaf and roots of *Talipariti tiliaceum*; (**j**,**k**) HTBM2490, two ascomata clinging on a brick with a subiculum soaked by dark algae (one manually raised from the brick, showing the detached subiculum traced by white arrows); (**l**) HKAS150755 (holotype), a group of ascomata growing on soil under *Heptapleurum arboricola* (see also (**n**,**r**)); (**m**) HTBM2491, two ascomata clinging on a stone (see also (**i**)); (**n**) close-up of the external surface of an ascoma from HKAS150755 (holotype) (see also (**l**,**r**)); (**o**) close-up of the longitudinal section of an ascoma from HTBM2447 (see also d), worm channels frequently present; (**p**) close-up of the subiculum surrounding an ascoma from HTBM2878 (see also (**c**)); (**q**) an ascospore from HKAS150755 (holotype) under SEM, note the fine, sparsely verrucose ornamentation; (**r**) ascomata from HKAS150755 (holotype) (see also (**l**,**n**)). (**s**) Microstructures in a longitudinal section, including the top part of hymenium (**top**), the transition area from hymenium to excipulum (**middle**), and the external surface (**bottom**). Bars: (**b**,**e**,**r**) = 1 cm, (**g**) = 1 mm, (**s**) = 10 µm.

(1) Fungi R.T. Moore.

(1.1) Ascomycota Caval.-Sm.

(1.1.1) Pezizomycetes.

(1.1.1.1) Pezizales.

(1.1.1.1.1) Pezizaceae.

(1.1.1.1.1.1) *Purpureodiscus* (G. Hirsch) Van Vooren.

(1.1.1.1.1.1.1) *Purpureodiscus masticophilus* Kun L. Yang, Jia Y. Lin & Zhu L. Yang, sp. nov. (Figure 6 and Table 10).

Registration identifier: FN573007.

Etymology: Referring to its morphology like chewing gums (anciently derived from mastic) when mature.

Type: China. Guangdong Province: Foshan City, Nanhai District, Qiandenghu Lake Park, 23°03′15″ N, 113°08′33″ E, elevation 10 m, 10 May 2025, Jia Y. Lin & Kun L. Yang, L25166 (HKAS150755, holotype; HTBM2448, isotype).

Diagnosis: Differing from the similar species *Purpureodiscus bananicola* [55,56] by darker subiculum, non-stratified excipulum and smaller, indistinctly ornamented ascospores.

Description: Ascomata tiny to small, sessile, often situated on a felty, merino white (#F9F5EC), thatch yellow (#F1ECC5) to butter orange (#F2DF8F) subiculum; odor indistinct; taste indistinct. Apothecium 5–20 mm broad, discoid at first, becoming applanate to convex, with a straight to undulate margin. Hymenium surface smooth to undulate, merino white (#F9F5EC), light apricot orange (#F7D7B2), eggshell orange (#F4C291), papaya red (#F99565), dark raspberry red (#B96F62) to sandal red (#BA8A73). External surface felty, brighter than hymenium surface. Context fleshy when fresh, becoming fragile after drying. Ascospores unicellular, {40/4/1} (14.5) 15–19 (19.5) [16.40 ± 1.16, 16.50] × 6.5–8.5 [7.09 ± 0.68, 6.50] µm, *Q* = 1.93–2.62 [2.33 ± 0.20, 2.54], oblong to subcylindrical, with abundant granular to globular contents, thin-walled to slightly thick-walled, nearly colorless, smooth under LM, but with fine, sparsely verrucose ornamentation under SEM, inamyloid. Asci cylindrical, operculate, eight-spored, unitunicate, nearly colorless, diffusely amyloid, 10.5–13 µm wide at sporiferous part, 200–270 µm long in total, with a usually curved basal part with a crozier. Paraphyses subcylindrical, unbranched, nearly colorless to slightly reddish to brownish, 195–240 × 5.5–10 µm, thin-walled, septate, tapering downwards. Subhymenium weakly differentiated, as an inner area of excipulum with slightly smaller inflated cells and thinner filamentous hyphae. Excipulum nearly colorless, composed of a textura globulosa-angularis to textura inflata by slightly thick-walled to thick-walled inflated cells (9–42 µm in diameter, abundant at hymenium side, becoming scarce towards the external surface) and filamentous hyphae (2.5–23 µm wide, scarce at hymenium side, becoming abundant towards the external surface). External surface composed of a tomentum to trichoderm by oblique to erect, thin-walled to slightly thick-walled, 5.5–16 µm wide, nearly colorless, unbranched or branched cystidioid cells or short filamentous hyphae.

Habit and distribution: Gregarious, seeming saprotrophic, found on various substrates (soil, stones, bricks, roots, branches, stems and barks, etc.), in subtropical forests or urban areas. Currently known from South to Central China.

Other collections examined: China. Hunan Province: Changsha City, exact location unknown, 15 June 2024, Wu-Ping Luo, Jia Y. Lin & Kun L. Yang, S24016 (HTBM2073). Guangdong Province: Foshan City, Nanhai District, Qiandenghu Lake Park, 23°03′15″ N, 113°08′33″ E, elevation 10 m, 10 May 2025, Jia Y. Lin & Kun L. Yang, L25165 (HTBM2447); same location, 11 May 2025, Jia Y. Lin & Kun L. Yang, L25167 (HTBM2449), K25022 (HTBM2874), K25025 (HTBM2877), K25026 (HTBM2878) and K25027 (HTBM2879); same location, 18 May 2025, L25207 (HTBM2489), L25208 (HTBM2490) and L25209 (HTBM2491).

Notes: The genus *Purpureodiscus* is recently circumscribed with four species, typified by *P. subisabellinus*, separating from *Peziza* s. str. with phylogenetic evidence [57]. This genus features sessile, fleshy and purplish apothecia, eight-spored, operculate, diffusely amyloid asci with a crozier, eguttulate, smooth to ornamented ascospores and an ectal excipulum composed of textura globulosa-angularis. Our relative collections are classified as a new species of this genus morphologically, and also supported by the sequences shown in Table 3 as closely related to *Purpureodiscus bananicola* in an nrLSU phylogeny.

The ascomata of this species only sporulate after reaching a substantial size and are frequently nested by worms. They are known to occur on a wide variety of substrates as described in habit and distribution. On plant substrates, particularly *Hibiscus rosa-sinensis* and *Talipariti tiliaceum*, a conspicuous, felty subiculum is usually present under the ascomata, but no adverse effects have been observed on these plants. It remains uncertain whether this fungus is epiphytic or endophytic.

**Figure 7:**
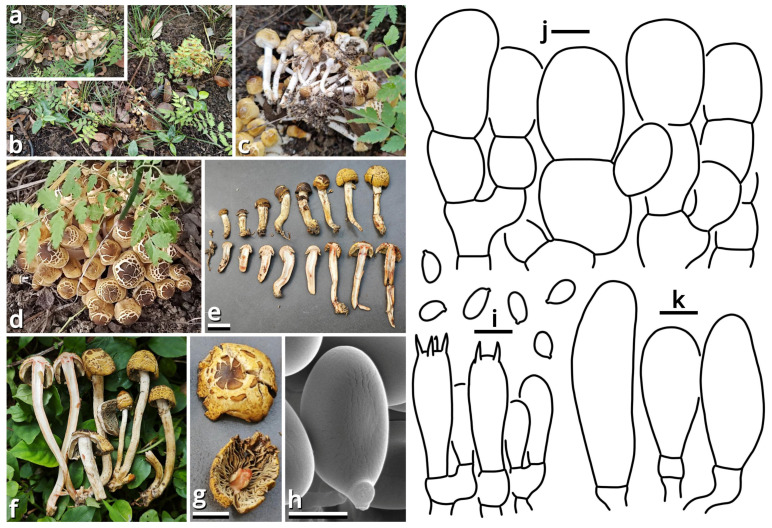
*Xanthagaricus popcorneus* (photos by Kun L. Yang & Cheng-Cheng Hu; drawings by Kun L. Yang from HKAS150767 (holotype)). (**a**,**b**) Habitat. (**c**–**g**) Basidiomata ((**c**,**d**,**f**) HKAS150767 (holotype); (**e**,**g**) HTBM2744). (**h**,**i**) Basidiospores and hymenium ((**h**) HTBM2744). (**j**) Pileus squamules. (**k**) Cheilocystidia. Bars: (**e**,**g**) = 1 cm, (**h**) = 2 µm, (**i**–**k**) = 5 µm.

In Haizhu District of Guangzhou City, during the summers of 2015 and 2020, Kun L. Yang found some cup fungi morphologically conspecific with this species on *Ficus* roots and *Ruellia* stems, respectively, but the collections are unavailable for sequencing.

(1.2) Basidiomycota R.T. Moore.

(1.2.1) Agaricomycetes Doweld.

(1.2.1.1) Agaricales Underw.

(1.2.1.1.1) Agaricaceae Chevall.

(1.2.1.1.1.1) *Xanthagaricus* (Heinem.) Little Flower, Hosag. & T.K. Abraham.

(1.2.1.1.1.1.1) *Xanthagaricus popcorneus* Kun L. Yang, Jia Y. Lin, Cheng-Cheng Hu & Zhu L. Yang, sp. nov. (Figure 7 and Table 10).

Registration identifier: FN572632.

Etymology: Referring to the pileus-like popcorns.

Type: China. Jiangsu Province: Nanjing City, Jiangning District, Nanjing University of Aeronautics and Astronautics (Jiangjun Road Campus), exact location unknown, 27 June 2025, Cheng-Cheng Hu, Kun L. Yang & Jia Y. Lin, L25461 (HKAS150767, holotype; HTBM2743, isotype).

Diagnosis: Differing from other species of *Xanthagaricus* by the sometimes special odor (similar to roasted sweet potatoes, roast chicken or chips), robust and strongly caespitose basidiomata, yellowish to brownish pileus with distinct flaky squamules, crowded brownish lamellae with a nearly smooth edge, context and stipe surface distinctly turning reddish after damage and smooth basidiospores sized 3.5–5 × 2.5–3.5 µm.

Description: Basidiomata small, robust, with context and stipe surface distinctly turning reddish after damage; odor indistinct or pleasant like roasted sweet potatoes, roast chicken or chips; taste unknown. Pileus 7–30 mm in diameter, hemispherical at first, becoming plano-convex, light cream orange (#FFF5E0) to milky yellow (#FFF07A) at background, covered with distinct, flaky, straw brown (#C4B179), dirty brass brown (#B47F5A), dull beaver brown (#92745E) to coffee red (#7B5B4E) squamules. Lamellae free to emarginate, crowded, butter orange (#F2DF8F) to kelp brown (#ACA47E), with a nearly smooth edge, interspersed with abundant lamellulae. Stipe 18–70 mm long, 3–6 mm thick, tapering downwards, more or less curved, slightly longitudinally striate with minute fibrous squamules, ceramic white (#FEFEFA), with a superior, easily broken and fugacious annulus. Context composed of a holomonomitic thigmoplect without clamp connections on hyphal septa. Basidiospores {40/2/2} 3.5–5 [4.40 ± 0.49, 4.00] × 2.5–3.5 [3.06 ± 0.25, 3.00] µm, *Q* = 1.33–1.67 [1.44 ± 0.11, 1.33], ellipsoid to oblong, slightly thick-walled, smooth under both LM and SEM, yellowish to brownish, with a small apiculus. Basidia 16–20 × 5–5.5 μm, clavate, two- or four-spored, thin-walled, nearly colorless. Lamella trama subregular, composed of 2–6 µm wide, thin-walled, nearly colorless to slightly brownish, compact, rarely to moderately branching hyphae. Cheilocystidia moderately abundant, 9–29 × 7–10 µm, clavate, thin-walled, smooth, nearly colorless. Pleurocystidia absent. Pileus squamules composed of a conioderm (epithelium) to subhymeniderm by subglobose, polygonal to elongate, slightly thick-walled and encrusted, yellowish to brownish inflated cells measured 6.5–24 × 6.5–20 µm.

Habit and distribution: Gregarious, strongly caespitose, saprotrophic on ground in subtropical to temperate forests. Currently known from East China.

Other collections examined: China. Jiangsu Province: Nanjing City, Jiangning District, Nanjing University of Aeronautics and Astronautics (Jiangjun Road Campus), exact location unknown, 27 June 2025, Cheng-Cheng Hu, Kun L. Yang & Jia Y. Lin, L25462 (HTBM2744) and L25463 (HTBM2745).

Notes: Presence or absence of basidiospore ornamentation and violetish tinge of basidiomata are important characteristics for species delimitation within *Xanthagaricus* [36]. Previously, there was only one species of *Xanthagaricus* known as having smooth basidiospores but without a violetish tinge, namely, *X. boluoshanensis* [36]. This new species, sister to *X. montgomeryensis* with 57% MLB in an ITS phylogeny, now adds the second.

**Figure 8:**
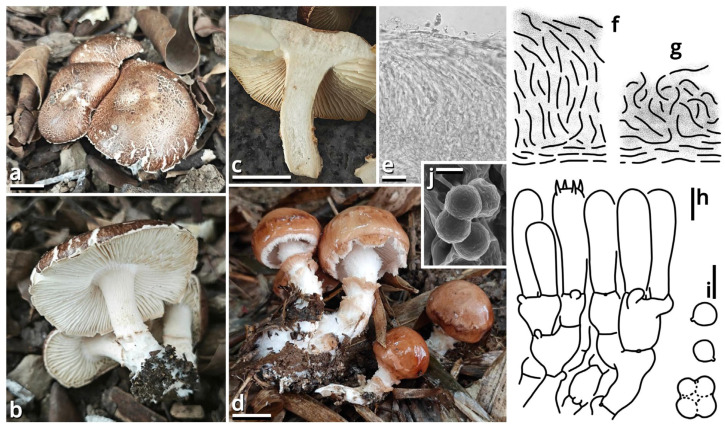
*Limacella yuexiuensis* (photos by Kun L. Yang; drawings by Kun L. Yang from HKAS150756 (holotype) and HTBM1240). (**a**–**d**) Basidiomata ((**a**–**c**) HKAS150756 (holotype) (note the damaged reddening coloration in (**c**)); (**d**) HTBM1240). (**e**–**g**) Pileipellis observed from dried collections ((**e**,**f**) Pileipellis of naturally moist basidiomata (in a moist state when found in the field, HTBM1240 (see also (**d**))), presenting as a plagiotrichoderm. (**g**) Pileipellis of naturally dried basidiomata (already in a dried state when found in the field, HKAS150756 (holotype) (see also (**a**–**c**)), presenting as a tomentum). (**h**) Hymenium and subhymenium. (**i**,**j**) Basidiospores, some agglutinated together by myxosporium ((**j**) HKAS150756 (holotype)). Bars: (**a**,**c**,**d**) = 1 cm, (**e**) = 20 µm, (**h**,**i**) = 5 µm, (**j**) = 2 µm.

**Figure 9:**
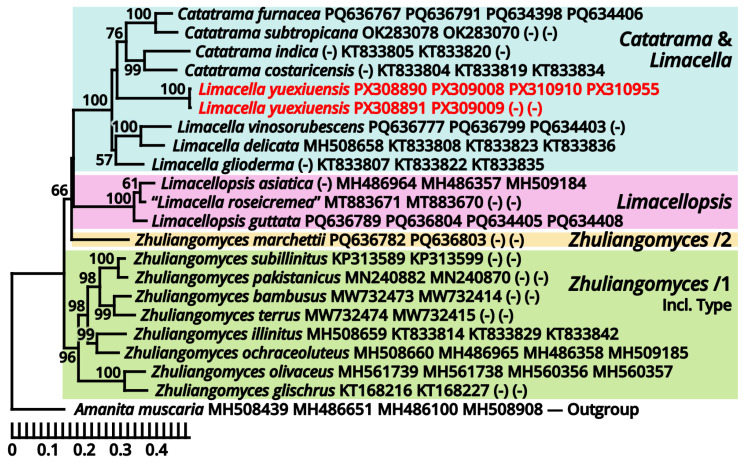
Phylogeny of *Catatrama*, *Limacella*, *Limacellopsis* and *Zhuliangomyces* based on four loci (ITS-nrLSU-*rpb2*-*tef-1α*), rooted with *Amanita* as outgroup. Nodes are annotated if supported by ≥50% MLB. Collections sequenced in this study are highlighted in red.

(1.2.1.1.2) Amanitaceae E.-J. Gilbert.

(1.2.1.1.2.1) *Limacella* Earle.

(1.2.1.1.2.1.1) *Limacella yuexiuensis* Kun L. Yang, Jia Y. Lin, Wen-Ju Ye & Zhu L. Yang, sp. nov. (Figure 8 and Figure 9 and Table 10).

Registration identifier: FN572633.

Etymology: Referring to the type locality.

Type: China. Guangdong Province: Guangzhou City, Yuexiu District, Dongshanhu Lake Park, in a parterre of a *Ficus* tree, 23°06′59″ N, 113°17′06″ E, elevation 1 m, 6 June 2025, Jia Y. Lin & Kun L. Yang, L25343 (HKAS150756, holotype; HTBM2625, isotype).

Diagnosis: Differing from other species of *Catatrama*, *Limacella*, *Limacellopsis* and *Zhuliangomyces* [58,59,60,61,62,63] by the sometimes special odor (similar to *Tessaratoma* stinkbugs or the agaric *Clitopilus crispus*), the context slightly turning reddish after damage, the stipe with several annuli, small basidiospores sized 3–3.5 × 2.5–3.5 µm and the habit of occurring under *Ficus* trees or in bamboo forests.

Description: Basidiomata small, with context slightly turning reddish after damage; odor fungal or similar to *Tessaratoma* stinkbugs or the agaric *Clitopilus crispus*; taste unknown. Pileus 16–39 mm in diameter, viscous and smooth when moist, becoming cracked, felty and even squamulose once dried, hemispherical at first, becoming plano-convex, light apricot orange (#F7D7B2), dark raspberry red (#B96F62) to sandal red (#BA8A73), exposing whitish context when cracked. Lamellae free to emarginate, sometimes with decurrent tooth, crowded, merino white (#F9F5EC) to ceramic white (#FEFEFA), with a more or less serrate edge, interspersed with abundant lamellulae. Stipe 18–34 mm long, 5–7 mm thick, subcylindrical, sometimes tapering upwards and bulbous at base, while sometimes expanded both at top and at base, slightly longitudinally striate, merino white (#F9F5EC) to ceramic white (#FEFEFA), with several annuli concolorous with the background or the pileus. Context composed of a holomonomitic thigmoplect with clamp connections on hyphal septa, with thromboplerous hyphae containing oil-like deuteroplasm sometimes present. Basidiospores {40/2/2} 3–3.5 [3.38 ± 0.22, 3.50] × 2.5–3.5 [3.06 ± 0.32, 3.50] µm, *Q* = 1.00–1.20 [1.11 ± 0.09, 1.17], globose, subglobose to broadly ellipsoid, thin-walled, slightly thick-walled to thick-walled, smooth under LM, rugose under SEM, often agglutinated together by myxosporium, nearly colorless, inamyloid, with a small apiculus. Basidia 14–25 × 4.5–6 μm, clavate, two- or four-spored, thin-walled, nearly colorless. Subhymenium composed of 1–3 layers of subglobose to ellipsoid, thin-walled, nearly colorless inflated cells sized 4.5–14 × 4–9.5 µm. Lamella trama bilateral, moderately compact, composed of a mediostratum by 3–8.5 µm wide, thin-walled to slightly thick-walled, nearly colorless, moderately branching hyphae, and two lateral strata each diverging at an angle of about 30 to 65 degree by 2.5–11 µm wide, thin-walled to slightly thick-walled, nearly colorless, moderately branching, more or less inflated hyphae. Cheilocystidia absent. Pleurocystidia absent. Pileipellis (observed from dried materials) composed of a gelatinized plagiotrichoderm to tomentum by thin-walled, 2–6 µm wide, nearly colorless to brownish, rarely, moderately to frequently branching hyphae.

Habit and distribution: Gregarious, saprotrophic on ground in parterres of *Ficus* trees or bamboos. Currently known from South China.

Other collection examined: China. Guangdong Province: Guangzhou City, Tianhe District, South China Agricultural University, in a parterre of bamboo, 23°09′38″ N, 113°20′46″ E, elevation 20 m, 15 September 2023, Wen-Ju Ye & Kun L. Yang, K23368 (HTBM1240).

Notes: *Catatrama*, *Limacella*, *Limacellopsis* and *Zhuliangomyces* are four closely related genera in a monophyletic group in Amanitaceae [60,61,62]. Among them, *Catatrama* and *Limacella* are sisters to each other, originally considered as distinctly different, that *Catatrama* has persisting veil elements on pileus and asperulate to warty basidiospores, while *Limacella* does not [59,60,61]. Recent phylogenetic analyses by Consiglio & Setti (2024) [63], however, revealed that some species morphologically belonging to *Limacella* are actually closer to the type species of *Catatrama* and thus were transferred to *Catatrama*. Our present four-locus phylogeny (Figure 9) reconfirms the result of Consiglio & Setti (2024) [63], and recognizes this new species as seeming to be a transition from *Limacella* to *Catatrama*, further blurring the boundary of the two genera. Given the basidiospores without distinct warts and the absence of inflated hyphae in pileus covering of this species, we recognize it in *Limacella* for the moment. If *Limacella* and *Catatrama* are synonymized together, *Limacella* also holds priority over *Catatrama*.

**Figure 10:**
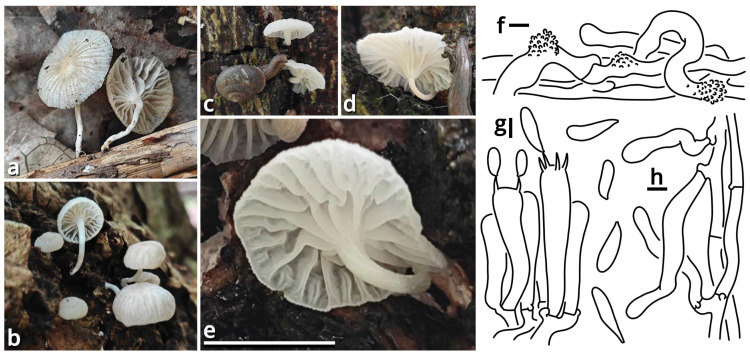
*Stygiomarasmius scandens* (photos by Kun L. Yang; drawings by Kun L. Yang from HTBM0776). (**a**–**e**) Basidiomata ((**a**) HTBM0776; (**b**) HTBM0778; (**c**–**e**) HTBM1236). (**f**) Pileipellis. (**g**) Hymenium. (**h**) Stipitipellis. Bars: (**e**) = 1 cm, (**f**–**h**) = 5 µm.

(1.2.1.1.3) Campanellaceae J.S. Oliveira, Desjardin & Moncalvo.

(1.2.1.1.3.1) *Stygiomarasmius* Kun L. Yang, Jia Y. Lin & Zhu L. Yang, gen. nov. (Figure 10 and Table 10).

Registration identifier: FN572630.

Type: *Stygiomarasmius scandens* (Massee) Kun L. Yang, Jia Y. Lin & Zhu L. Yang (see Section 1.2.1.1.3.1.1).

Etymology: Referring to the pathogenic nature and the creeping, destructive hyphal cords or rhizomorphs [64,65] of this marasmioid taxon.

Diagnosis: Differing from other taxa of Campanellaceae by the ability to produce basidiomata with a developed stipe, strong and pathogenic hyphal cords or rhizomorphs, and very long (see Section 1.2.1.1.3.1.1) basidiospores.

Description: Basidiomata tiny to small, sessile or pseudostipitate or laterally, eccentrically to centrally stipitate, without a distinct color change after damage. Pileus circular on eccentrically to centrally stipitate basidiomata, flabelliform to reniform on sessile or pseudostipitate or laterally stipitate basidiomata, pale, felty or viscous, groovily striate. Lamellae distant to subdistant, pale, with a smooth edge, interspersed with lamellulae, frequently interveined. Stipe when present, tapering downwards, more or less curved, whitish, felty. Context composed of a holomonomitic ixoplect with clamp connections on hyphal septa. Basidiospores ellipsoid to lacrymoid, thin-walled, smooth, nearly colorless, inamyloid. Basidia clavate, two-, three- or four-spored, nearly colorless. Lamella trama subregular to irregular, gelatinized. Pleurocystidia absent or indistinguishable, or similar to cheilocystidia. Cheilocystidia present, with coralloid modifications. Pileipellis composed of a tomentocutis, clavicutis to epidermoid cutis. Stipitipellis generally composed of a rectocutis, but locally issuing caulocystidia and thus locally rendering a tomentum, tomentocutis to trichoderm.

**Figure 11:**
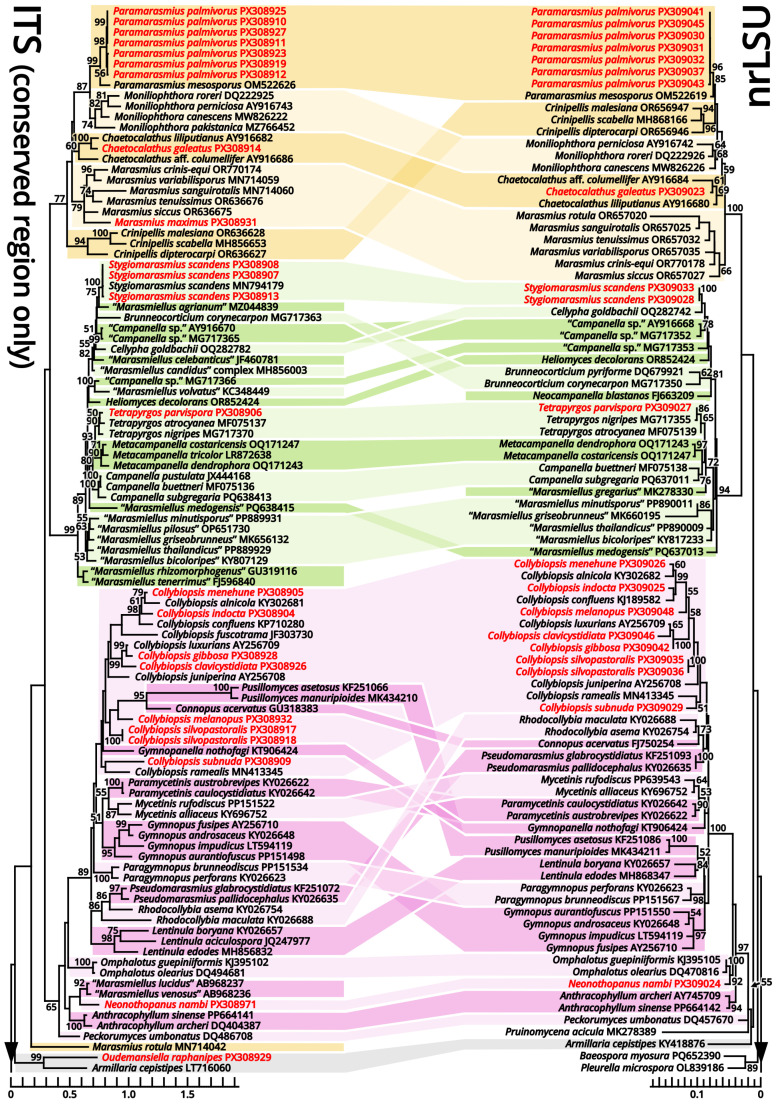

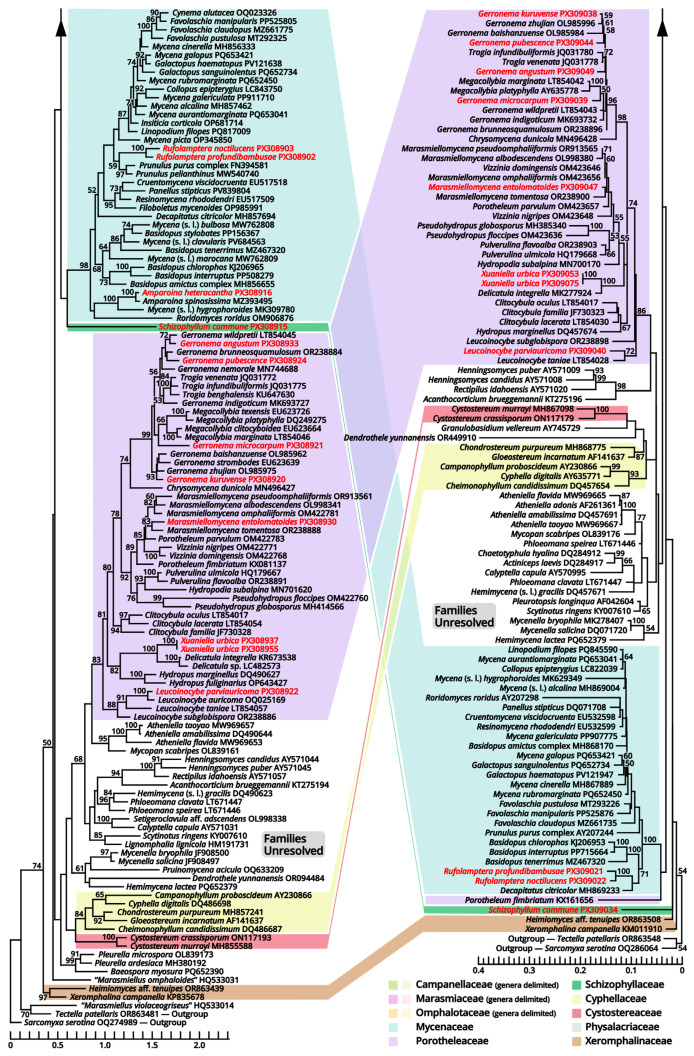
Comparative phylogenies focusing on recently accepted but ambiguous genera of Marasmiineae, Mycenineae and Schizophyllineae, rooted with Sarcomyxineae as outgroup. Nodes are annotated if supported by ≥50% MLB. Collections sequenced in this study are highlighted in red.

Notes: Oliveira et al. (2024) [66] reported a gorgeously comprehensive study on taxonomy of Marasmiaceae s. l., in which the genera *Brunneocorticium*, *Campanella*, *Neocampanella*, *Tetrapyrgos* and the so-called *Marasmiellus candidus* group were found to represent a distinct lineage separating from Marasmiaceae s. str. and Omphalotaceae, circumscribed as Campanellaceae by pale pileus, interveined lamellae, non-chitinous stipe and variform basidiospores. Petersen & Hughes (2025) [67] added one more genus *Metacampanella* to this family later with molecular evidence. In our present phylogeny (Figure 11), more collections labeled as *Marasmiellus* or *Heliomyces* are found to nest in Campanellaceae, but most are also undetermined in generic position. Some of our collections cluster with a topotype of the famous plant pathogen recently named *Marasmiellus scandens* (GH-80 from Ghana, ITS sequence available from MN794179 [64,65]) as a single phylogenetic species, representing a new monotypic genus, proposed as *Stygiomarasmius* here.

(1.2.1.1.3.1.1) *Stygiomarasmius scandens* (Massee) Kun L. Yang, Jia Y. Lin & Zhu L. Yang, comb. nov. (Figure 10 and Table 10).

Registration identifier: FN572629.

Basionym: *Marasmius scandens* Massee, *Bulletin of Miscellaneous Informations of the Royal Botanical Gardens Kew*: 1 (1910) (≡*Marasmiellus scandens* (Massee) Dennis & D.A. Reid, *Kew Bulletin* 12[11](2): 289 (1957)).

Notes: The type of this species was collected on branches of *Theobroma cacao* in Ghana [68]. Our collections are found on *Syzygium cumini* and *Talipariti tiliaceum*, generally exhibiting a well-developed stipe with a circular pileus and very long, lacrymoid basidiospores (13–19 × 3–4 µm, *Q* = 3.71–5.00) (Figure 10). They significantly differ from the description in the protologue of *Marasmius scandens* [68] and the combination *Marasmiellus scandens* [69] that outline a usually poorly developed stipe with a flabelliform to reniform pileus and ellipsoid basidiospores. Interestingly, the observation by Zhang et al. (2023) [70] on other collections identified as this species, isolated as endophytes from healthy living stem of *Aquilaria sinensis* from South China, recorded an intermediate basidiospore size between ours and Massee (1910) [68].

Given the conspecific phylogeny of our collections with well-studied topotype materials by Amoako-Attah et al. (2016, 2020) [64,65] (Figure 11), we recognize the differences as intraspecific at the moment and add the features from our collections to the generic definition. Cheilocystidia of our collections are in a gelatinized collapsed status and thus are not recorded to avoid misinterpretations. Pleurocystidia are present near the lamella edge but absent elsewhere in our collections, similar to cheilocystidia, and are also collapsed. Hyphal cords or rhizomorphs are weakly developed in our collections.

(1.2.1.1.3.2) *Campanella* Henn.

(1.2.1.1.3.2.1) *Campanella subgregaria* (Shun Liu & B. Zhu) Kun L. Yang, Jia Y. Lin & Zhu L. Yang, comb. nov.

Registration identifier: FN572599.

Basionym: *Marasmiellus subgregarius* Shun Liu & B. Zhu, in Liu, Pan, Cui & Zhu, *Mycology*: 10.1080/21501203.2025.2471382, 38 (2025).

Notes: This species described by Liu et al. (2025) [71] fits the morphological concept of genus *Campanella* [66]. Its holotype (Liu582 from China, ITS sequence available from PQ638413) also cluster with the epitype of *C. buettneri* (DED8276 from Sao Tome and Principe, ITS sequence available from MF075136 [72]), type species of *Campanella*, in our present phylogeny (Figure 11). The transfer is thus made.

**Figure 12:**
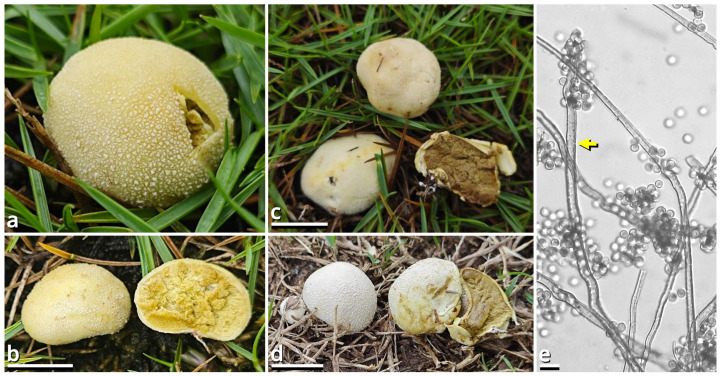
*Tortoperdon suspectum* (photos by Kun L. Yang). (**a**–**d**) Basidiomata ((**a**,**b**) HTBM1229; (**c**) HKAS150757 (holotype); (**d**) HTBM1266). (**e**) Capillitia and basidiospores of HKAS150757 (holotype), with a yellow arrow pointing a pit. Bars: (**b**–**d**) = 1 cm, (**e**) = 10 µm.

(1.2.1.1.4) Lycoperdaceae Bercht. & J. Presl.

(1.2.1.1.4.1) *Tortoperdon* Kun L. Yang, Jia Y. Lin & Zhu L. Yang, gen. nov. (Figure 12 and Table 10).

Registration identifier: FN572635.

Type: *Tortoperdon suspectum* Kun L. Yang, Jia Y. Lin & Zhu L. Yang (see Section 1.2.1.1.4.1.1).

Etymology: Referring to the basidiomata-like cakes.

Diagnosis: Differing from other genera of Lycoperdaceae by the brightly colored basidiomata without a sterile base, basidiospores not or shortly pedicellate and without distinct ornamentation, and capillitia of *Lycoperdon*-type to *Bovista*-*Lycoperdon* intermediate type.

Description: Basidiomata tiny to small, sessile, more or less subglobose to tuberiform, without a sterile base. Exoperidium present as a thin and easily removable layer of floccose to furfuraceous squamules. Endoperidium brightly colored, fragile. Gleba cottony, becoming brownish when mature. Basidiospores subglobose to broadly ellipsoid, thick-walled, smooth under LM, brownish, not or shortly pedicellate. Capillitia *Lycoperdon*-type to *Bovista*-*Lycoperdon* intermediate type, rarely septate, rarely branching, thick-walled and slightly encrusted, brownish, with abundant pits. Exoperidium composed of a spherocystoderm to conioderm. Endoperidium radially subregular, composed of colored hyphae without clamp connections.

Notes: Apart from morphology, this genus is distinct from all currently recognized genera of Lycoperdaceae [73,74] as supported by sequences (Table 3) and does not have a known relative in current phylogeny. It currently comprises two species. See Sections 1.2.1.1.4.1.1–1.2.1.1.4.1.2 for details.

(1.2.1.1.4.1.1) *Tortoperdon suspectum* Kun L. Yang, Jia Y. Lin & Zhu L. Yang, sp. nov. (Figure 12 and Table 10).

Registration identifier: FN572634.

Etymology: We did try to recognize this common species as *Bovista citrina* but finally found it unacceptable (see Notes).

Type: China. Guangdong Province: Guangzhou City, Nansha District, South Waterfront Cape (Nanbin Shuijiao) Park, 22°46′48″ N, 113°30′51″ E, elevation 1 m, September 10, 2023, Kun L. Yang & Jia Y. Lin, K23356 (HKAS150757, holotype; HTBM1228, isotype).

Diagnosis: Differing from *Tortoperdon citrinum* (see Section 1.2.1.1.4.1.2) by larger basidiospores without a distinct pedicel, and thinner capillitia with abundant pits (see Notes).

Description: Basidiomata tiny to small, 7–19 mm in diameter, 5.5–12 mm in height, sessile, oblately subglobose to tuberiform, without a sterile base; odor indistinct; taste unknown. Exoperidium present as a thin and easily removable layer of pure white (#FFFFFF) to ceramic white (#FEFEFA), floccose to furfuraceous squamules. Endoperidium 0.5–1 mm, moon yellow (#FAF9AA), milky yellow (#FFF07A) to cardamon yellow (#E0E091), fragile. Gleba cottony, concolorous with peridium at first, becoming straw brown (#C4B179) to dull olive brown (#736938). Basidiospores {40/3/2} 3.5–5.5 [4.25 ± 0.52, 4.00] × 3–5 [3.61 ± 0.67, 3.00] µm, *Q* = 1.00–1.33 [1.19 ± 0.10, 1.33], subglobose to broadly ellipsoid, thick-walled, smooth under LM, brownish, not or shortly pedicellate. Capillitia 2–5.5 μm wide, *Lycoperdon*-type to *Bovista*-*Lycoperdon* intermediate type, rarely septate, rarely branching, thick-walled and slightly encrusted, brownish, with abundant pits. Exoperidium composed of a spherocystoderm to conioderm by subglobose, thin-walled to slightly thick-walled, nearly colorless inflated cells 14–42 µm in diameter. Endoperidium radially subregular, composed of 1.5–5 μm wide, more or less thick-walled, sometimes encrusted, yellowish to brownish, compact, moderately to frequently branching hyphae without clamp connections.

Habit and distribution: Usually gregarious, occasionally solitary, saprotrophic on ground on lawns or grasslands of Poaceae plants. Currently known from South and East China, and probably also from Japan (GenBank accession no. AB067724, as *Bovista pusilla*).

Other collections examined: China. Zhejiang Province: Hangzhou City, Xixi Wetland, exact location unknown, 10 June 2023, Qing-Qing Huang, S23119 (HTBM0362). Fujian Province: Fuzhou City, Minhou County, Minhou Riverside Ecological Park, exact location unknown, 12 June 2023, Fei-Yang Chen, S23124 (HTBM0367) and S23125 (HTBM0368). Guangxi Zhuang Autonomous Region: Nanning City, Jiangnan Park, exact location unknown, 13 June 2023, Jun-Jie Liu, S23148 (HTBM0391). Guangdong Province: Guangzhou City, Nansha District, South Waterfront Cape (Nanbin Shuijiao) Park, 22°46′48″ N, 113°30′51″ E, elevation 1 m, 10 September 2023, Kun L. Yang & Jia Y. Lin, K23357 (HTBM1229). Guangdong Province: Guangzhou City, Haizhu District, Pazhou Pagoda Park, 23°06′05″ N, 113°22′16″ E, elevation 5 m, 20 August 2024, Jia Y. Lin & Kun L. Yang, L23285 (HTBM1266).

Notes: This species resembles *Tortoperdon citrinum* (see Section 1.2.1.1.4.1.2) described from Sri Lanka but differing from its protologue [75] by larger basidiospores without a distinct pedicel. Such differences are further supported by a monograph of Lycoperdaceae members in China by Fan (2019) [76], which gave a consistent description acknowledging the distinctly pedicellate basidiospores for “*Bovista citrina*”. Fan (2019) [76] also described the capillitia of “*Bovista citrina*” as without pits and 6–10 μm in thickness, while capillitia of our collections abundantly possess pits and are thinner (Figure 12).

(1.2.1.1.4.1.2) *Tortoperdon citrinum* (Berk. & Broome) Kun L. Yang, Jia Y. Lin & Zhu L. Yang, comb. nov.

Registration identifier: FN572636.

Basionym: *Lycoperdon citrinum* Berk. & Broome, *Journal of the Linnean Society, Botany* 14(74): 80 (1873) [1875].

Notes: This species does not have available sequence data. However, given its significant morphological and biogeographic relationships with *Tortoperdon suspectum*, we propose this transfer.

(1.2.1.1.5) Mycenaceae Overeem.

Notes: The majority of this family has been well studied with morphological concepts [77,78,79]. In a gorgeous and modern monograph of Mycenaceae by Bau et al. (2021) [80], eleven genera were accepted in this family, namely, *Cruentomycena*, *Favolaschia*, *Flabellimycena*, *Hemimycena*, *Mycena*, *Panellus*, *Resinomycena*, *Roridomyces*, *Sarcomyxa*, *Tectella* and *Xeromphalina*. Updated with recent phylogenetic studies [81,82,83], *Flabellimycena*, *Hemimycena*, *Sarcomyxa*, *Tectella* and *Xeromphalina* are now excluded from Mycenaceae, leaving the remaining six genera. However, these six genera are still difficult to delimit from each other at present, mainly due to the seeming non-monophyletic nature of the species-rich genus *Mycena* [80,83,84,85,86]. Therefore, we inferred three phylogenies in different scales with broad sampling for both the current *Mycena* species and some genera near Mycenaceae to approach a classification system better in monophyly for identification of our related collections.

**Figure 13:**
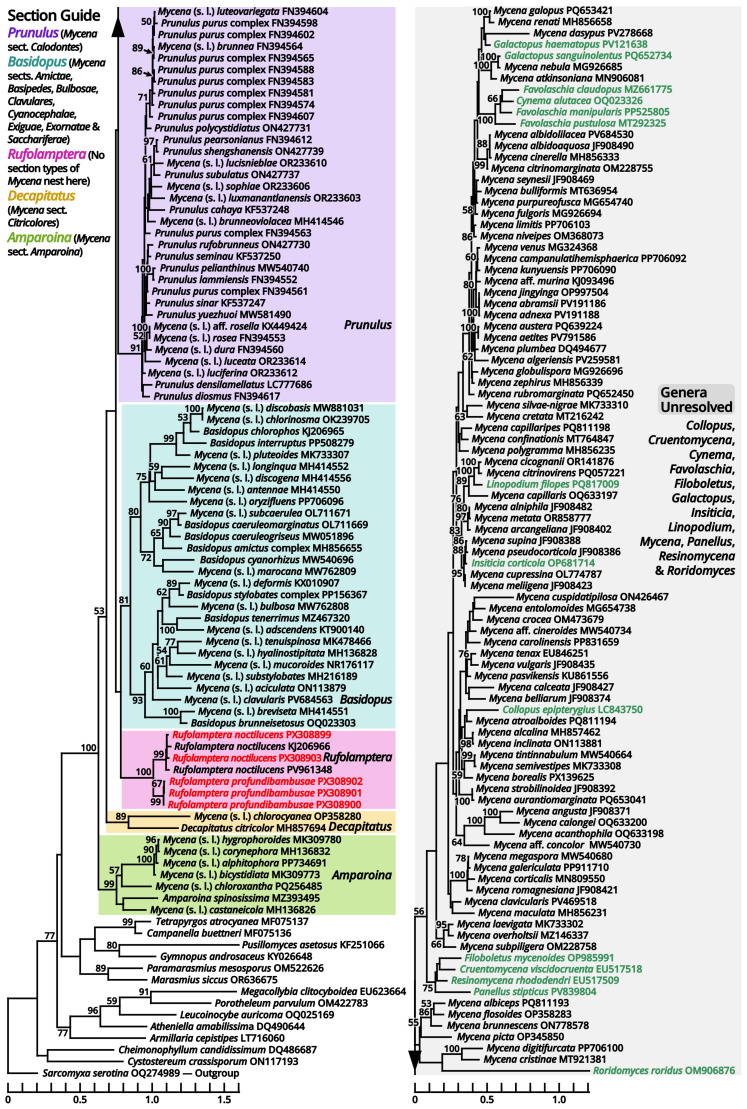
Phylogeny focusing on recently accepted *Mycena* species within Mycenaceae based on conserved region of ITS locus, rooted with *Sarcomyxa* (Sarcomyxineae) as outgroup. Nodes are annotated if supported by ≥50% MLB. Collections sequenced in this study are highlighted in red. Collections of unresolved genera not labeled as *Mycena* are highlighted in green.

**Figure 14:**
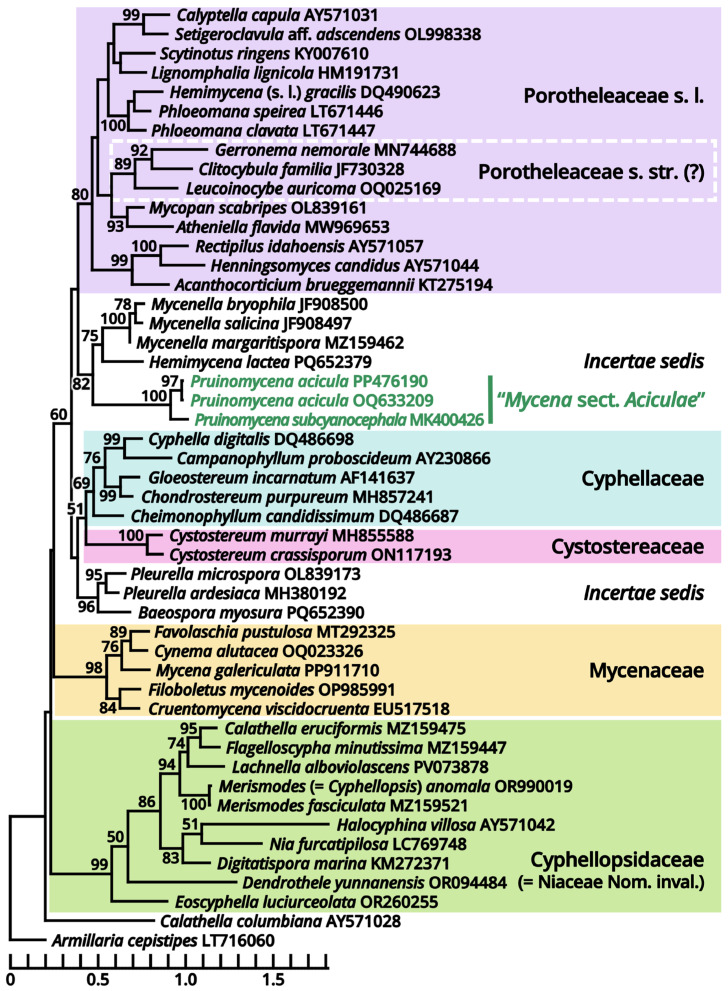
Phylogeny focusing on recently accepted *Mycena* species now excluded from Mycenaceae (highlighted in green) based on conserved region of ITS locus, rooted with *Armillaria* (Physalarcriaceae) as outgroup. Nodes are annotated if supported by ≥50% MLB.

According to our present phylogenies, the abovementioned six genera (*Cruentomycena*, *Favolaschia*, *Mycena*, *Panellus*, *Resinomycena* and *Roridomyces*) plus a monotypic genus *Cynema* establish a significantly supported clade representing Mycenaceae (Figure 11). Within this family, *Mycena* is found to be a strongly polyphyletic genus, distinguished as six parts as follows (Figure 13): (1) the separate genus *Amparoina*, a name previously synonymized with *Mycena*, corresponding to Mycena sect. Amparoina (see Section 1.2.1.1.5.1); (2) the separate genus Decapitatus, a name previously synonymized with Mycena, corresponding to Mycena sect. Citricolores (see Section 1.2.1.1.5.2); (3) the separate genus Rufolamptera, a new genus comprising our collections of two species (see Section 1.2.1.1.5.3); (4) the separate genus Basidopus, a name previously synonymized with Mycena, corresponding to Mycena sects. Amictae, Basipedes, Bulbosae, Clavulares, Cyanocephalae, Exiguae, Exornatae and Sacchariferae (see Section 1.2.1.1.5.4); (5) the separate genus Prunulus, a name previously synonymized with Mycena, corresponding to Mycena sect. Calodontes (see Section 1.2.1.1.5.5); and (6) the remaining part, a polyphyletic mass poor in phylogenetic signals, including the type species of Mycena and other recently accepted genera of Mycenaceae (see Section 1.2.1.1.5.6). Given the fact that part (6) includes several widely accepted genera without distinct genetic distance, it becomes clear that Mycena should be split sooner or later. In this study, we commented on this revised frame of Mycenaceae with 17 potential genera based on present phylogenies (*Amparoina*, *Basidopus*, *Collopus*, *Cruentomycena*, *Cynema*, *Decapitatus*, *Favolaschia*, *Filoboletus*, *Galactopus*, *Insiticia*, *Linopodium*, *Mycena*, *Panellus*, *Prunulus*, *Resinomycena*, *Roridomyces* and *Rufolamptera*), used it to identify our collections and made selected combinations of our necessaries. A total of 12 significant generic names either synonymized with or threatening these 17 generic names applied in this study are listed in Table 13 for reference of further studies. Lastly, the recently accepted section *Aciculae* of *Mycena* is now excluded from Mycenaceae following our present phylogenies (Figure 11 and Figure 14), dealt with in Section 1.2.1.1.9.2 as a genus of incertae sedis.

(1.2.1.1.5.1) *Amparoina* Singer.

Notes: This genus corresponds to *Mycena* sect. *Amparoina*, equal to the former sect. *Sacchariferae* stirps *Amparoina* plus *Alphitophora* [87,88]. It is characterized by slender basidiomata with a pileus with furfuraceous squamules, a stipe bulbous or forming a disc at base and the presence of cherocytes in some species. In our present phylogeny (Figure 13), seven species, including the type species of *Amparoina* (*A. spinosissima*), establish an isolated clade distant from the type species of *Mycena* (*M. galericulata*), and thus *Amparoina* may be recovered as a separate genus.

(1.2.1.1.5.2) *Decapitatus* Redhead & Seifert.

Notes: This genus was proposed by Redhead et al. (2000) [89] to accommodate *Stilbum flavidum* Cooke (1880) [90], the anamorph of *Mycena citricolor* (≡*Agaricus citricolor* Berk. & M.A. Curtis (1868) [1869]). Since the application of Melbourne Code, *Decapitatus* had become a synonym of *Mycena* due to the priority, corresponding to *Mycena* sect. *Citricolores* [91]. In our present phylogeny (Figure 13), *M. citricolor* and *M. chlorocyanea* form an isolated clade distant from the type species of *Mycena* (*M. galericulata*). This clade may be recovered as *Decapitatus*, but given the unique habit and asexual morphology of *M. citricolor* [90,91,92], a careful evaluation on other potential members is necessary. As the earlier name of the type species (*Agaricus citricolor*) lacks a binomial combination in this genus, *Decapitatus citricolor* is proposed below.

(1.2.1.1.5.2.1) *Decapitatus citricolor* (Berk. & M.A. Curtis) Kun L. Yang, Jia Y. Lin & Zhu L. Yang, comb. nov.

Registration identifier: FN572600.

**Figure 15:**
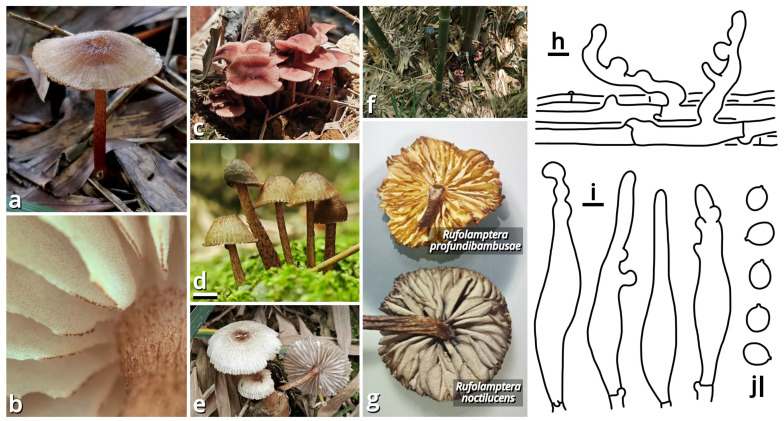
*Rufolamptera profundibambusae*, compared with *R. noctilucens* (photos by Kun L. Yang & Jia Y. Lin; drawings by Kun L. Yang from HKAS150760 (holotype)). (**a**–**e**) Basidiomata ((**a**) HTBM0921; (**b**,**e**) HKAS150760 (holotype); (**c**) HTBM2235; (**d**) HTBM2233). (**f**) Habitat (HTBM2235). (**g**) A special characteristic to distinguish *R. profundibambusae* from its similar species *R. noctilucens*, that lamellae of *R. noctilucens* turn grayish after drying (represented by a basidioma from HTBM2224), while lamellae of *R. profundibambusae* do not (represented by a basidioma from HKAS150760 (holotype)). (**h**) Pileipellis. (**i**) Cheilocystidia. (**j**) Basidiospores. Bars: (**d**) = 1 cm, (**h**–**j**) = 5 µm.

Basionym: *Agaricus citricolor* Berk. & M.A. Curtis, *Journal of the Linnean Society, Botany* 10(45): 285 (1868) [1869] (≡*Mycena citricolor* (Berk. & M.A. Curtis) Sacc., *Sylloge Fungorum* 5: 263 (1887); =*Stilbum flavidum* Cooke, *Grevillea* 9(49): 11 (1880); =*Decapitatus flavidus* (Cooke) Redhead & Seifert, in Redhead et al., *Taxon* 49(4): 795 (2000)).

(1.2.1.1.5.3) *Rufolamptera* Kun L. Yang, Jia Y. Lin & Zhu L. Yang, *gen. nov.* (Figure 15 and Table 10).

Registration identifier: FN572638.

Type: *Rufolamptera profundibambusae* Kun L. Yang, Jia Y. Lin & Zhu L. Yang (see Section 1.2.1.1.5.3.1).

Etymology: Referring to the reddish-brown basidiomata with bioluminescent potential of this group.

Diagnosis: Differing from other genera of *Mycena* s. l. by the combination of reddish-brown and relatively robust basidiomata, pileus and stipe with distinct fibrous squamules, elements of pileipellis and stipitipellis with rostrate to capitate modifications, and presence of pleurocystidia.

Description: Basidiomata small, without a distinct color change after damage; odor indistinct; taste unknown. Pileus paraboloid to plano-convex, depressed at center, covered with fibrous squamules, reddish brown, groovily striate. Lamellae emarginate to sinuate, sometimes with decurrent tooth, subdistant, pale, but with a serrate, dark edge, interspersed with lamellulae, interveined. Stipe subcylindrical, more or less curved, covered with fibrous squamules, reddish brown. Context composed of a holomonomitic thigmoplect, often with clamp connections on hyphal septa. Basidiospores more or less ellipsoid, smooth, nearly colorless, amyloid, with a small apiculus. Basidia clavate, thin-walled, nearly colorless. Lamella trama subregular to irregular. Cheilocystidia present. Pleurocystidia present. Pileipellis composed of a rectocutis to tomentocutis by hyphae with rostrate to capitate modifications. Stipitipellis similar to pileipellis.

Notes: This genus currently comprises two species, the new species Rufolamptera profundibambusae proposed below (see Section 1.2.1.1.5.3.1) and the previously described Mycena noctilucens Corner (see Section 1.2.1.1.5.3.2). We further found that M. noctilucens may actually be a complex with more cryptic species pending to be resolved (see Section 1.2.1.1.5.3.2), and thus *R. profundibambusae* is selected as the generic type to avoid potential ambiguity.

(1.2.1.1.5.3.1) *Rufolamptera profundibambusae* Kun L. Yang, Jia Y. Lin & Zhu L. Yang, sp. nov. (Figure 15 and Table 10).

Registration identifier: FN572637.

Etymology: Referring to its habit of occurring deep in bamboo forests.

Type: China. Guangdong Province: Foshan City, Nanhai District, Qiandenghu Lake Park, 23°03′18″ N, 113°08′26″ E, elevation 1 m, 11 May 2025, Kun L. Yang & Jia Y. Lin, K25034 (HKAS150760, holotype; HTBM2886, isotype).

Diagnosis: Differing from *Rufolamptera noctilucens* [93,94] (see Section 1.2.1.1.5.3.2) by fewer and sparser squamules on pileus and stipe, lamellae not turning grayish after drying, rare presence of pleurocystidia and the bamboo-associated habit.

Description: Basidiomata small, without a distinct color change after damage; odor indistinct; taste unknown. Pileus 10–42 mm in diameter, paraboloid at first, becoming plano-convex, depressed at center, alabaster white (#F9F9F9), merino white (#F9F5EC) to lotus-root orange (#F5E9D9) at background, covered with fibrous, dark raspberry red (#B96F62) to dull beaver brown (#92745E) squamules, groovily striate. Lamellae emarginate to sinuate, sometimes with decurrent tooth, subdistant, alabaster white (#F9F9F9), merino white (#F9F5EC) to light apricot orange (#F7D7B2), not turning grayish after drying, spotted with tiny dark dots of pleurocystidia, with a serrate, dark raspberry red (#B96F62) to dull beaver brown (#92745E) edge, interspersed with abundant lamellulae, interveined. Stipe 26–62 mm long, 1.5–3.5 mm thick, subcylindrical and expanded at apex, more or less curved, merino white (#F9F5EC) to lotus-root orange (#F5E9D9) at background, covered with fibrous, dark raspberry red (#B96F62) to dull beaver brown (#92745E) squamules. Context composed of a holomonomitic thigmoplect, often with clamp connections on hyphal septa. Basidiospores {40/3/2} (6) 6.5–7.5 [7.13 ± 0.38, 7.50] × (4.5) 5–6 [5.35 ± 0.36, 5.50] µm, *Q* = 1.25–1.50 [1.33 ± 0.07, 1.27], broadly ellipsoid to ellipsoid, thin-walled, smooth, nearly colorless, amyloid, frequently containing granular globular contents, with a small apiculus. Basidia 14–22 × 5.5–6.5 μm, clavate, four-spored, thin-walled, nearly colorless. Lamella trama subregular to irregular, composed of 3–24 µm wide, thin-walled to slightly thick-walled, nearly colorless, compact, rarely to moderately branching hyphae. Cheilocystidia very abundant, 25–100 × 6.5–14 µm, lageniform, often with rostrate to capitate modifications, thin-walled to slightly thick-walled, smooth, yellowish to brownish. Pleurocystidia rare, similar to cheilocystidia. Pileipellis composed of a rectocutis to tomentocutis by thin-walled, 1.5–6.5 µm wide, brownish, rarely to moderately branching hyphae sometimes with rostrate to capitate modifications. Stipitipellis similar to pileipellis.

Habit and distribution: Usually gregarious, occasionally solitary, saprotrophic on bamboo remains in bamboo forests. Currently known from South China.

Other collections examined: China. Guangdong Province: Guangzhou City, Tianhe District, South China Agricultural University, Lit Bamboos, 23°09′49″ N, 113°21′46″ E, elevation 40 m, 19 June 2023, Jia Y. Lin & Kun L. Yang, L23088 (HTBM0921). Guangdong Province: Foshan City, Nanhai District, Qiandenghu Lake Park, 23°03′18″ N, 113°08′26″ E, elevation 1 m, 26 August 2024, Kun L. Yang & Jia Y. Lin, L24248 (HTBM2233) and L24250 (HTBM2235); same location, 28 June 2025, Jia Y. Lin & Kun L. Yang, L25458 (HTBM2740).

Notes: Whether this species is bioluminescent like its sister taxon *R. noctilucens* remains unknown. We collected them in the daytime and have not paid attention to its bioluminescent ability.

(1.2.1.1.5.3.2) *Rufolamptera noctilucens* (Corner) Kun L. Yang, Jia Y. Lin & Zhu L. Yang, comb. nov. (Figure 15 and Table 10).

Registration identifier: FN572639.

Basionym: *Mycena noctilucens* Corner, *Transactions of the British Mycological Society* 37(3): 264 (1954).

Collections examined: China. Guangdong Province: Guangzhou City, Haizhu District, in a parterre of a *Phoenix* tree, 23°04′02″ N, 113°19′18″ E, elevation 10 m, 24 August 2024, Kun L. Yang & Jia Y. Lin, L24239 (HTBM2224). Guangdong Province: Guangzhou City, Yuexiu District, Dongshanhu Lake Park, in a parterre of a *Ficus* tree, 23°06′59″ N, 113°17′06″ E, elevation 1 m, 6 June 2025, Kun L. Yang & Jia Y. Lin, L25341 (HTBM2623).

Notes: This species was described by Corner (1954) [93] from Micronesia, without a distinct substrate preference like *Rufolamptera profundibambusae* [94]. In our present phylogeny (Figure 13), one collection from Malaysia (ACL054, ITS sequence available from KJ206966 [94]), one collection from India (PYKM82, ITS sequence available from PV961348, direct submission by Yuvarani & Kumaresan), and two of our collections found on *Phoenix* roots (HTBM2224) or *Ficus* roots (HTBM2623) in South China, cluster into a significantly supported clade that can be recognized as this species. However, the internal genetic distance of this clade looks heterogenous (e.g., the similarity between KJ206966 and PV961348 is 97.58%), and it seems that four collections represent four cryptic species. This issue should be evaluated with more collections made in future.

Interestingly, the lamellae of our collections HTBM2224 and HTBM2623 consistently turned grayish after drying, while lamellae of *R. profundibambusae* did not (Figure 15). We recognized this reaction as a characteristic to distinguish the two taxa.

Corner (1954) [93] described *R. noctilucens* as luminescent with a pale blue light. Chew et al. (2014) [94], however, recorded a yellowish-green light. Whether our collections are bioluminescent remains unknown. We collected them in the daytime and have not paid attention to their bioluminescent ability.

(1.2.1.1.5.4) *Basidopus* Earle.

Notes: As stated in the protologue [95], this genus is typified by *Mycena stylobates*, corresponding to *Mycena* sect. *Basipedes*, and thus features a stipe bulbous or forming a disc at base. In our present phylogeny (Figure 13), *M. stylobates* cluster with the type species of *Mycena* sect. *Bulbosae* (*M. bulbosa*), sect. *Clavulares* (*M. clavularis*) and sect. *Sacchariferae* (*M. tenerrima*) into a single significantly supported clade, and this clade is sister to another significantly supported clade that comprises the type species of sect. *Amictae* (*M. amicta*), sect. *Cyanocephalae* (*M. interrupta*), sect. *Exiguae* (*M. marocana*) and sect. *Exornatae* (*M. chlorophos*). These two clades further establish a large significantly supported clade distant from the type species of *Mycena* (*M. galericulata*), comprising species usually with tiny and slender basidiomata, a stipe bulbous or forming a disc at base and absence of cherocytes, and thus *Basidopus* may deserve a separate genus. The type species (*Agaricus stylobates*) still lacks a binomial combination in this genus.

(1.2.1.1.5.4.1) *Basidopus stylobates* (Pers.) Kun L. Yang, Jia Y. Lin & Zhu L. Yang, comb. nov.

Registration identifier: FN572601.

Basionym: *Agaricus stylobates* Pers., *Synopsis Methodica Fungorum (Göttingen)* 2: 390 (1801) (≡*Mycena stylobates* (Pers.) P. Kumm., *Der Führer in die Pilzkunde (Zerbst)*: 108 (1871)).

(1.2.1.1.5.4.2) *Basidopus amictus* (Fr.) Kun L. Yang, Jia Y. Lin & Zhu L. Yang, comb. nov.

Registration identifier: FN572602.

Basionym: *Agaricus amictus* Fr., *Systema Mycologicum* 1: 141 (1821) (≡*Mycena amicta* (Fr.) Quél., *Mémoires de la Société d’Émulation de Montbéliard, Ser. 2* 5: 243 (1872)).

Notes: This name is recombined based on the phylogenetic evidence of the collection CBS:352.50 (from France, ITS sequence available from MH856655 [96]). It actually represents a large complex containing several cryptic undescribed species as revealed by our collections. We cannot resolve this complex due to the insufficient collections, so this name is tentatively cited in Table 5.

The stipe base of this complex, which is sometimes rooted rather than forming a disc, seems contrary to the majority of *Basidopus*. We consider it acceptable in this genus by the presence of intermediates like *Basidopus caeruleogriseus*, *B*. *caeruleomarginatus* and *B. cyanorhizus* (stipe base neither forming a distinct disc nor rooted [79,97]; see Sections 1.2.1.1.5.4.3–1.2.1.1.5.4.5). Such differences may be related to substrate preferences, for example, the genus *Oudemansiella* (Physalacriaceae) with similar cases, in which *O. mucida* complex growing on aerial substrates exhibits a bulbous to disced stipe base, while *O. radicata* complex growing on buried substrates possesses a rooted stipe [98].

(1.2.1.1.5.4.3) *Basidopus caeruleogriseus* (Q. Na, Y.P. Ge & H. Zeng) Kun L. Yang, Jia Y. Lin & Zhu L. Yang, comb. nov.

Registration identifier: FN572603.

Basionym: *Mycena caeruleogrisea* Q. Na, Y.P. Ge & H. Zeng, in Na, Liu, Zeng, Ke, Song, Cheng & Ge, *MycoKeys* 90: 126 (2022).

Notes: This name is recombined based on the phylogenetic evidence of its holotype FFAAS0001 (from China, ITS sequence available from MW051896 [97]).

(1.2.1.1.5.4.4) *Basidopus caeruleomarginatus* (Q. Na & Y.P. Ge) Kun L. Yang, Jia Y. Lin & Zhu L. Yang, comb. nov.

Registration identifier: FN572604.

Basionym: *Mycena caeruleomarginata* Q. Na & Y.P. Ge, in Na, Liu, Zeng, Ke, Song, Cheng & Ge, *MycoKeys* 90: 131 (2022).

Notes: This name is recombined based on the phylogenetic evidence of its holotype FFAAS0357 (from China, ITS sequence available from OL711669 [97]).

(1.2.1.1.5.4.5) *Basidopus cyanorhizus* (Quél.) Kun L. Yang, Jia Y. Lin & Zhu L. Yang, comb. nov.

Registration identifier: FN572605.

Basionym: *Mycena cyanorhiza* Quél., *Mémoires de la Société d’Émulation de Montbéliard, Ser. 2* 5: 436 (1875).

Notes: This name is recombined based on the phylogenetic evidence of the collection J24082010 (from Finland, ITS sequence available from MW540696, direct submission by Dima et al.).

(1.2.1.1.5.4.6) *Basidopus chlorophos* (Berk. & M.A. Curtis) Kun L. Yang, Jia Y. Lin & Zhu L. Yang, comb. nov.

Registration identifier: FN572606.

Basionym: *Agaricus chlorophos* Berk. & M.A. Curtis, *Proceedings of the American Academy of Arts and Sciences* 4: 113 (1860) (≡*Mycena chlorophos* (Berk. & M.A. Curtis) Sacc., *Sylloge Fungorum* 5: 301 (1887)).

Notes: This name is recombined based on the phylogenetic evidence of the collection ACL051 (from Malaysia, ITS sequence available from KJ206965 [94]).

(1.2.1.1.5.4.7) *Basidopus interruptus* (Berk.) Kun L. Yang, Jia Y. Lin & Zhu L. Yang, comb. nov.

Registration identifier: FN572607.

Basionym: *Agaricus interruptus* Berk., in Hooker, *Botany of the Antarctic Voyage III Flora Tasmaniae* 2: 243 (1859) [1860] (≡*Mycena interrupta* (Berk.) Sacc., *Sylloge Fungorum* 5: 299 (1887)).

Notes: This name is recombined based on the phylogenetic evidence of the collection MEL2497283 (from Australia, ITS sequence available from PP508279, direct submission by Holmes).

(1.2.1.1.5.4.8) *Basidopus brunneisetosus* (Corner) Kun L. Yang, Jia Y. Lin & Zhu L. Yang, comb. nov.

Registration identifier: FN572608.

Basionym: *Mycena brunneisetosa* Corner, *Beihefte zur Nova Hedwigia* 109: 172 (1994).

Notes: This name is recombined based on the phylogenetic evidence of the collection LE-BIN3329 (from Vietnam, ITS sequence available from OQ023303, direct submission by Senik & Manzhieva).

(1.2.1.1.5.4.9) *Basidopus tenerrimus* (Berk.) Kun L. Yang, Jia Y. Lin & Zhu L. Yang, comb. nov.

Registration identifier: FN572609.

Basionym: *Agaricus tenerrimus* Berk., in Smith, *The English Flora (Edn 2) (London)* 5(2): 61 (1836) (≡ *Mycena tenerrima* (Berk.) Quél., *Mémoires de la Société d’Émulation de Montbéliard, Ser. 2* 5: 151 (1872)).

Notes: This name is recombined based on the phylogenetic evidence of the collection G.M.2014-09-30.5 (from Luxembourg, ITS sequence available from MZ467320, direct submission by Hermant & Marson).

(1.2.1.1.5.4.10) *Basidopus indigoticus* (C.L. Wei & R. Kirschner) Kun L. Yang, Jia Y. Lin & Zhu L. Yang, comb. nov.

Registration identifier: FN573033.

Basionym: *Mycena indigotica* C.L. Wei & R. Kirschner, *Mycoscience* 60: 11 (2018) [2019].

Notes: This name is recombined based on its close relationship to *Basidopus amictus* and *B. chlorophos* according to the protologue.

(1.2.1.1.5.5) *Prunulus* Gray.

Notes: This genus is lectotypified by *Agaricus denticulatus* (=*Prunulus pelianthinus*), corresponding to *Mycena* sect. *Calodontes* [99]. In our present phylogeny (Figure 13), *Prunulus pelianthinus* and other members of *Mycena* sect. *Calodontes* establish a large significantly supported clade distant from the type species of *Mycena* (*M. galericulata*) and featuring purplish-brown, relatively robust basidiomata, smooth and more or less umbonate pileus and nearly smooth stipe [100,101,102], and thus *Prunulus* may deserve a separate genus.

(1.2.1.1.5.5.1) *Prunulus pearsonianus* (Dennis ex Singer) Kun L. Yang, Jia Y. Lin & Zhu L. Yang, comb. nov.

Registration identifier: FN572610.

Basionym: *Mycena pearsoniana* Dennis ex Singer, *Sydowia* 12(1–6): 233 (1959) [1958].

Notes: This name is recombined based on the phylogenetic evidence of the collection JV06890 (from Denmark, ITS sequence available from FN394612, direct submission by Harder).

(1.2.1.1.5.5.2) *Prunulus densilamellatus* (Nagamune, S. Kigawa & N. Endo) Kun L. Yang, Jia Y. Lin & Zhu L. Yang, comb. nov.

Registration identifier: FN572611.

Basionym: *Mycena densilamellata* Nagamune, S. Kigawa & N. Endo, in Nagamune et al., *Mycoscience* 65(3): 118 (2024).

Notes: This name is recombined based on the phylogenetic evidence of its ex-holotype SuR20190914-112 (from Japan, ITS sequence available from LC777686 [103]).

(1.2.1.1.5.5.3) *Prunulus cahaya* (A.L.C. Chew & Desjardin) Kun L. Yang, Jia Y. Lin & Zhu L. Yang, comb. nov.

Registration identifier: FN572612.

Basionym: *Mycena cahaya* A.L.C. Chew & Desjardin, *Mycologia* 106(5): 979 (2014).

Notes: This name is recombined based on the phylogenetic evidence of its holotype KLU-M:1221 (from Malaysia, ITS sequence available from KF537248 [84]).

(1.2.1.1.5.5.4) *Prunulus sinar* (A.L.C. Chew & Desjardin) Kun L. Yang, Jia Y. Lin & Zhu L. Yang, comb. nov.

Registration identifier: FN572613.

Basionym: *Mycena sinar* A.L.C. Chew & Desjardin, *Mycologia* 106(5): 983 (2014).

Notes: This name is recombined based on the phylogenetic evidence of its paratype KLU-M:1220 (from Malaysia, ITS sequence available from KF537247 [84]).

(1.2.1.1.5.5.5) *Prunulus sinar* var. *tangkaisinar* (A.L.C. Chew & Desjardin) Kun L. Yang, Jia Y. Lin & Zhu L. Yang, comb. nov.

Registration identifier: FN572614.

Basionym: *Mycena sinar* var. *tangkaisinar* A.L.C. Chew & Desjardin, *Mycologia* 106(5): 984 (2014).

Notes: This name is together recombined with the type variety of *Mycena sinar* (see Section 1.2.1.1.5.5.4) [84].

(1.2.1.1.5.5.6) *Prunulus seminau* (A.L.C. Chew & Desjardin) Kun L. Yang, Jia Y. Lin & Zhu L. Yang, comb. nov.

Registration identifier: FN572615.

Basionym: *Mycena seminau* A.L.C. Chew & Desjardin, *Mycologia* 106(5): 985 (2014).

Notes: This name is recombined based on the phylogenetic evidence of its holotype KLU-M:1223 (from Malaysia, ITS sequence available from KF537250 [84]).

(1.2.1.1.5.5.7) *Prunulus polycystidiatus* (Z.W. Liu, Y.P. Ge, L. Zou & Q. Na) Kun L. Yang, Jia Y. Lin & Zhu L. Yang, comb. nov.

Registration identifier: FN572616.

Basionym: *Mycena polycystidiata* Z.W. Liu, Y.P. Ge, L. Zou & Q. Na, in Liu, Ge, Zeng, Cheng & Na, *MycoKeys* 93: 31 (2022).

Notes: This name is recombined based on the phylogenetic evidence of its holotype FFAAS0417 (from China, ITS sequence available from ON427731 [101]).

(1.2.1.1.5.5.8) *Prunulus rufobrunneus* (Z.W. Liu, Y.P. Ge & Q. Na) Kun L. Yang, Jia Y. Lin & Zhu L. Yang, comb. nov.

Registration identifier: FN572617.

Basionym: *Mycena rufobrunnea* Z.W. Liu, Y.P. Ge & Q. Na, in Liu, Ge, Zeng, Cheng & Na, *MycoKeys* 93: 36 (2022).

Notes: This name is recombined based on the phylogenetic evidence of its holotype FFAAS0416 (from China, ITS sequence available from ON427730 [101]).

(1.2.1.1.5.5.9) *Prunulus shengshanensis* (Z.W. Liu, Y.P. Ge & Q. Na) Kun L. Yang, Jia Y. Lin & Zhu L. Yang, comb. nov.

Registration identifier: FN572618.

Basionym: *Mycena shengshanensis* Z.W. Liu, Y.P. Ge & Q. Na, in Liu, Ge, Zeng, Cheng & Na, *MycoKeys* 93: 41 (2022).

Notes: This name is recombined based on the phylogenetic evidence of its holotype FFAAS0424 (from China, ITS sequence available from ON427739 [101]).

(1.2.1.1.5.5.10) *Prunulus subulatus* (Z.W. Liu, Y.P. Ge & Q. Na) Kun L. Yang, Jia Y. Lin & Zhu L. Yang, comb. nov.

Registration identifier: FN572619.

Basionym: *Mycena subulata* Z.W. Liu, Y.P. Ge & Q. Na, in Liu, Ge, Zeng, Cheng & Na, *MycoKeys* 93: 46 (2022).

Notes: This name is recombined based on the phylogenetic evidence of its holotype FFAAS0423 (from China, ITS sequence available from ON427737 [101]).

(1.2.1.1.5.5.11) *Prunulus yuezhuoi* (Z.W. Liu, Y.P. Ge & Q. Na) Kun L. Yang, Jia Y. Lin & Zhu L. Yang, comb. nov.

Registration identifier: FN572620.

Basionym: *Mycena yuezhuoi* Z.W. Liu, Y.P. Ge & Q. Na, in Liu, Na, Cheng, Wu & Ge, *Phytotaxa* 511(2): 153 (2021).

Notes: This name is recombined based on the phylogenetic evidence of its paratype FFAAS0344 (from China, ITS sequence available from MW581490 [100]).

(1.2.1.1.5.6) *Collopus*, *Cruentomycena*, *Cynema*, *Favolaschia*, *Filoboletus*, *Galactopus*, *Insiticia*, *Linopodium*, *Mycena*, *Panellus*, *Resinomycena* and *Roridomyces.*

Notes: As our present phylogeny shown (Figure 13), the type of these 12 genera actually or probably nest in a polyphyletic mass dominated by a large number of *Mycena*-labeled species (many of which feature a tomentose stipe base) poor in phylogenetic signals. We have tried to proceed with multilocus sequencing on our collections related to this group but found they quite frequently obtained multipeaked results. This problem is expected to be resolved by genome sequencing in the near future, following the milestones set by Ke et al. (2020) [85] and Harder et al. (2024) [104]. The type species of *Linopodium* (*Agaricus filopes*) still lacks a binomial combination with *Linopodium*. To ensure that our label of “*Linopodium filopes*” in Figure 11 and Figure 13 makes sense, we propose it below.

(1.2.1.1.5.6.1) *Linopodium filopes* (Bull.) Kun L. Yang, Jia Y. Lin & Zhu L. Yang, comb. nov.

Registration identifier: FN572621.

Basionym: *Agaricus filopes* Bull., *Herbier de la France (Paris)* 7: pl. 320 (1788) (≡*Mycena filopes* (Bull.) P. Kumm., *Der Führer in die Pilzkunde (Zerbst)*: 110 (1871)).

(1.2.1.1.6) Omphalotaceae Bresinsky.

(1.2.1.1.6.1) *Collybiopsis* (J. Schröt.) Earle.

Notes: The type species of *Collybiopsis* (J. Schröt.) Earle (1909) (≡*Marasmius* III *Collybiopsis* J. Schröt., in Cohn 1889) and *Marasmiellus* Murrill (1915) nest in a single generic clade. For the genus they represent, *Collybiopsis* has priority over *Marasmiellus* [105,106].

**Figure 16:**
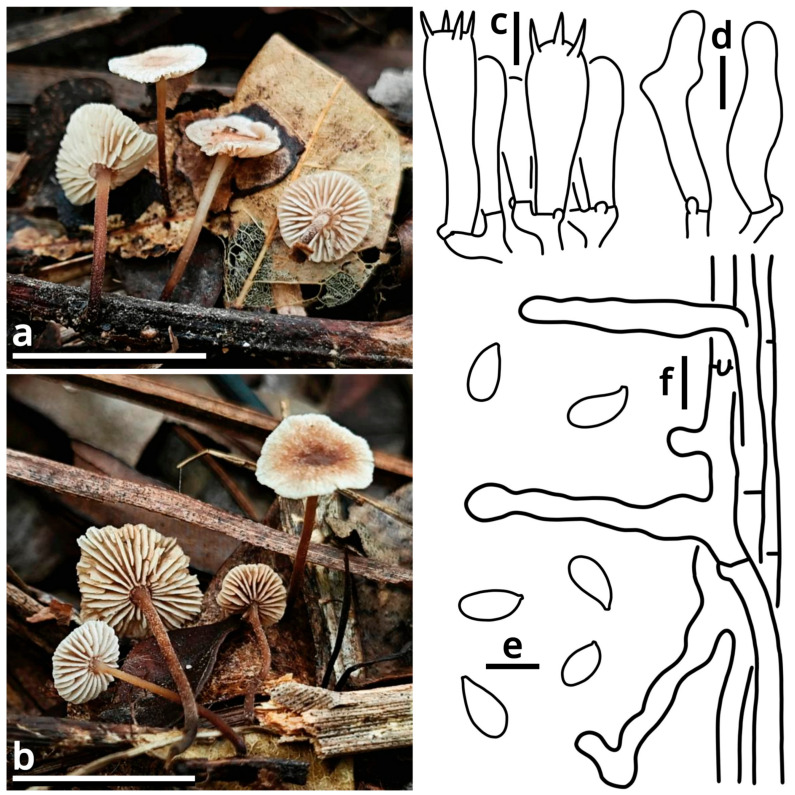
*Collybiopsis silvopastoralis* (photos by Kun L. Yang; drawings by Kun L. Yang from HKAS150758 (holotype) and HTBM3041). (**a**,**b**) Basidioma ((**a**) HKAS150758 (holotype); (**b**) HTBM3041). (**c**) Hymenium. (**d**) Cheilocystidia. (**e**) Basidiospores. (**f**) Stipitipellis. Bars: (**a**,**b**) = 1 cm, (**c**–**f**) = 5 µm.

(1.2.1.1.6.1.1) *Collybiopsis silvopastoralis* Kun L. Yang, Jia Y. Lin & Zhu L. Yang, sp. nov. (Figure 16 and Table 10).

Registration identifier: FN572640.

Etymology: Referring to the collection site, a forest for silvopasture.

Type: China. Guangdong Province: Huizhou City, Huiyang District, Qianfeng Village, 22°58′33″ N, 114°36′13″ E, elevation 150 m, 31 July 2025, Kun L. Yang, K25138 (HKAS150758, holotype; HTBM3040, isotype).

Diagnosis: Differing from other species of *Collybiopsis* by the indistinct odor, tiny, felty to pubescent and brownish basidiomata with a more or less depressed pileus, basidiospores sized 7–8.5 × (3) 3.5–4 µm, rare presence of cheilocystidia and pileipellis composed of a rectocutis to tomentocutis.

Description: Basidiomata tiny; odor indistinct; taste unknown. Pileus 4–8 mm in diameter, applanate, more or less depressed at center, felty to pubescent, ceramic white (#FEFEFA), merino white (#F9F5EC), dirty brass brown (#B47F5A) to coffee red (#7B5B4E), with a slightly groovily striate margin. Lamellae emarginate to sinuate, sometimes with decurrent tooth, nearly crowded, merino white (#F9F5EC) to beach yellow (#F9F3C9), with a serrate edge, interspersed with abundant lamellulae. Stipe 8–13 mm long, 0.5–1 mm thick, subcylindrical or slightly tapering downwards, more or less curved, felty to pubescent, merino white (#F9F5EC) to beach yellow (#F9F3C9) at upper part, becoming dirty brass brown (#B47F5A) to coffee red (#7B5B4E) downwards. Context composed of a holomonomitic thigmoplect to ixoplect, often with clamp connections on hyphal septa. Basidiospores {40/2/2} 7–8.5 [7.83 ± 0.45, 8.00] × (3) 3.5–4 [3.58 ± 0.24, 3.50] µm, *Q* = 1.88–2.43 [2.20 ± 0.15, 2.29], more or less lacrymoid, thin-walled, smooth, nearly colorless, inamyloid, with a small apiculus. Basidia 18–24 × 4.5–6.5 μm, clavate, two-, three- or four-spored, thin-walled, nearly colorless. Lamella trama subregular, composed of 2.5–6 µm wide, thin-walled to slightly thick-walled, nearly colorless, compact, rarely to moderately branching hyphae. Cheilocystidia rare, 23–27 × 4–6.5 µm, generally clavate, but often with capitate modifications, thin-walled to slightly thick-walled, smooth, nearly colorless. Pleurocystidia rare, similar to cheilocystidia. Pileipellis composed of a rectocutis to tomentocutis by thin-walled, slightly thick-walled to thick-walled, sometimes encrusted, 2–10 µm wide, more or less brownish, rarely to moderately branching hyphae. Stipitipellis composed of a pseudotrichoderm by slightly thick-walled to thick-walled, 3.5–7 µm wide, brownish, flexuous, non-septate hyphal branches issued by an underlying rectocutis.

Habit and distribution: Gregarious, saprotrophic on litterfall in subtropical forests. Currently known from China.

Other collection examined: Guangdong Province: Huizhou City, Huiyang District, Qianfeng Village, 22°58′33″ N, 114°36′13″ E, elevation 150 m, 31 July 2025, Kun L. Yang, K25139 (HTBM3041).

Notes: Morphological characteristics collaborated with sequences (Table 3) and phylogeny (Figure 11) support this species as new.

(1.2.1.1.6.1.2) *Collybiopsis alnicola* (J.L. Mata & Halling) Kun L. Yang, Jia Y. Lin & Zhu L. Yang, comb. nov.

Registration identifier: FN572622.

Basionym: *Gymnopus alnicola* J.L. Mata & Halling [as “*alnicolus*”], in Mata, Halling & Petersen, *Fungal Diversity* 16: 115 (2004) (≡*Marasmiellus alnicola* (J.L. Mata & Halling) J.S. Oliveira, in Oliveira et al., *Mycological Progress* 18(5): 734 (2019)).

Notes: This species described by Mata et al. (2004) [107] fits the morphological concept of genus *Collybiopsis* [66,105,106]. Its collection near type locality (URM90019 from Brazil, ITS sequence available from KY302681, direct submission by Coimbra et al.) also nests within the clade dominated by *Collybiopsis* species in our present phylogeny (Figure 11). The transfer is thus made.

(1.2.1.1.6.1.3) *Collybiopsis fuscotrama* (Mešić, Tkalčec & Chun Y. Deng) Kun L. Yang, Jia Y. Lin & Zhu L. Yang, comb. nov.

Registration identifier: FN572623.

Basionym: *Gymnopus fuscotramus* Mešić, Tkalčec & Chun Y. Deng, in Mešić et al., *Mycotaxon* 117: 324 (2011) (≡*Marasmiellus fuscotramus* (Mešić, Tkalčec & Chun Y. Deng) J.S. Oliveira, in Oliveira et al., *Mycological Progress* 18(5): 734 (2019)).

Notes: This species described by Mešić et al. (2011) [108] fits the morphological concept of genus *Collybiopsis* [66,105,106]. Its holotype (GDGM26313 from China, ITS sequence available from JF303730) also nests within the clade dominated by *Collybiopsis* species in our present phylogeny (Figure 11). The transfer is thus made.

**Figure 17:**
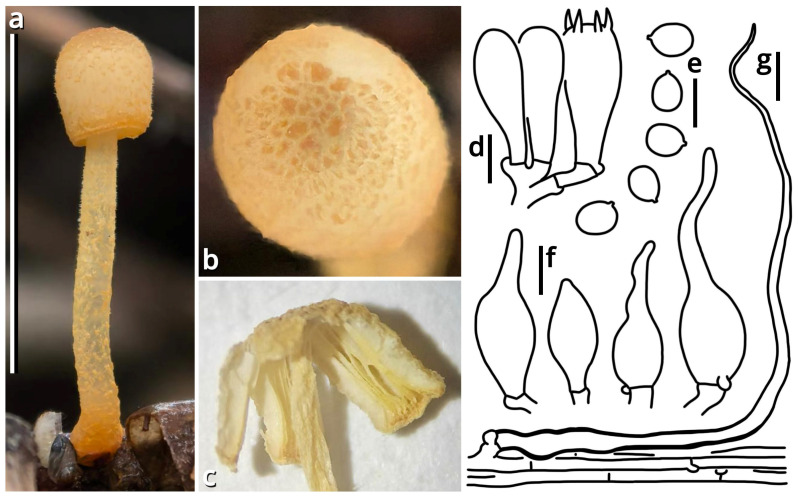
*Leucoinocybe parviauricoma* (photos by Kun L. Yang; drawings by Kun L. Yang from HKAS150759 (holotype)). (**a**–**c**) Basidioma of HKAS150759 (holotype) ((**c**) in air-dried state). (**d**) Hymenium. (**e**) Basidiospores. (**f**) Cheilocystidia. (**g**) Pileipellis. Bars: (**a**) = 1 cm, (**d**–**g**) = 5 µm.

(1.2.1.1.7) Porotheleaceae Murrill.

(1.2.1.1.7.1) *Leucoinocybe* Singer ex Antonín, Borov., Holec & Kolařík.

(1.2.1.1.5.3.1) *Leucoinocybe parviauricoma* Kun L. Yang, Jia Y. Lin & Zhu L. Yang, sp. nov. (Figure 17 and Table 10).

Registration identifier: FN572641.

Etymology: Referring to its basidioma like that of *Leucoinocybe auricoma* but smaller.

Type: China. Guangdong Province: Huizhou City, Huiyang District, Qianfeng Village, 22°58′33″ N, 114°36′13″ E, elevation 150 m, 31 July 2025, Kun L. Yang, K25156 (HKAS150759, holotype; HTBM3058, isotype).

Diagnosis: Differing from *Leucoinocybe auricoma* by the tiny basidioma and generally smaller size of other structures.

Description: Basidioma tiny, without a distinct color change after damage; odor indistinct; taste unknown. Pileus 3 mm in diameter, cylindrical, light cream orange (#FFF5E0) at background, covered with floccose to fibrous, bread orange (#F3C374) to honey orange (#FFAC2A) squamules. Lamellae free, crowded, light cream orange (#FFF5E0), with a very slightly serrate, yolk orange (#FEDC3D) edge, interspersed with scarce lamellulae. Stipe 12 mm long, 1.5 mm thick, subcylindrical, slightly bulbous at base, subtransparently whitish at background, covered with furfuraceous, bread orange (#F3C374) to honey orange (#FFAC2A) squamules. Context composed of a seeming holomonomitic thigmoplect to ixoplect, with clamp connections on hyphal septa. Basidiospores {20/1/1} 4.5–5 [4.70 ± 0.24, 5.00] × 3–3.5 [3.20 ± 0.24, 3.00] µm, *Q* = 1.43–1.50 [1.47 ± 0.03, 1.50], ellipsoid, thin-walled, smooth, nearly colorless, seeming amyloid, with a small apiculus. Basidia 11.5–18 × 5–5.5 μm, clavate, two-, three- or four-spored, thin-walled, nearly colorless. Lamella trama subregular, composed of 3–12 µm wide, thin-walled to slightly thick-walled, nearly colorless, compact, rarely to moderately branching hyphae. Cheilocystidia moderately abundant, 11–24 × 5.5–7.5 µm, lageniform with a long rostrate apex, thin-walled, smooth, nearly colorless to slightly yellowish or orangish. Pleurocystidia absent. Pileipellis generally composed of a rectocutis by thin-walled, 2–4.5 µm wide, nearly colorless, rarely to moderately branching hyphae, but locally issuing clusters of lageniform, thick-walled, yellowish to orangish, thromboplerous cystidia 32–125 µm long and 2–4.5 µm at widest part and thus locally rendering a trichoderm. Stipitipellis similar to pileipellis.

Habit and distribution: Solitary, saprotrophic on dead branches in subtropical forests. Currently known from South China.

Notes: Morphological characteristics collaborated with sequences (Table 3) and phylogeny (Figure 11) support this species as new.

**Figure 18:**
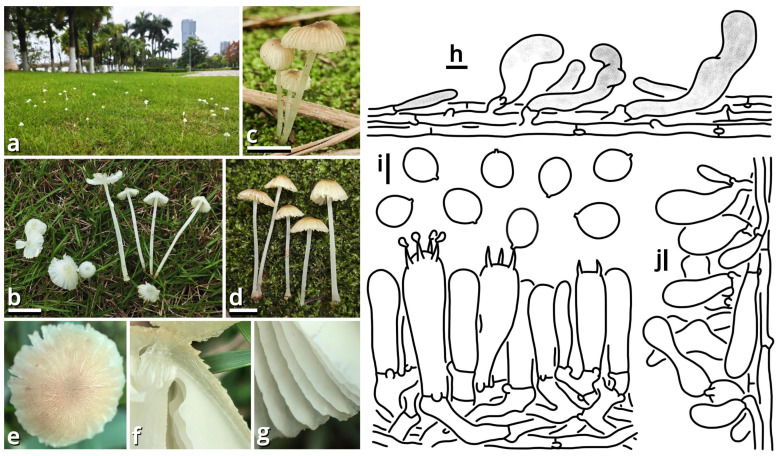
*Xuaniella urbica* (photos by Kun L. Yang; drawings by Kun L. Yang from HKAS150761 (holotype)). (**a**,**b**) Basidiomata growing on *Zoysia* lawns (HTBM1996). (**c**,**d**) Basidiomata growing in bamboo gardens ((**c**) HTBM2897; (**d**) HTBM2884). (**e**–**g**) Close-ups of basidioma characteristics (**e**) HTBM2884; (**f**,**g**) HTBM2536). (**h**) Pileipellis. (**i**) Basidiospores, hymenium, subhymenium and lamella trama. (**j**) Stipitipellis. Bars: (**b**–**d**) = 1 cm, (**h**–**j**) = 5 µm.

(1.2.1.1.7.2) *Xuaniella* Kun L. Yang, Jia Y. Lin & Zhu L. Yang, gen. nov. (Figure 18 and Table 10).

Registration identifier: FN572643.

Type: *Xuaniella urbica* Kun L. Yang, Jia Y. Lin & Zhu L. Yang (see Section 1.2.1.1.7.2.1).

Etymology: Implying the similarity with the close genus *Atheniella*, as the goddess Jiu Tian Xuan Nü (Goddess Xuan of the Empyrean) in Chinese mythology is possibly identical to Athena in Greek mythology.

Diagnosis: Differing from *Atheniella* [109,110] by larger basidiomata without hymenial cystidia and from *Delicatula* [111,112,113,114] by larger and vividly colored basidiomata with developed lamellae producing subglobose to broadly ellipsoid basidiospores.

Description: Basidiomata tiny to small, without a distinct color change after damage. Pileus paraboloid, campanulate, plano-convex to applanate, more or less umbonate or papillary at center, colored, covered with minute squamules. Lamellae sinuate to decurrent, subdistant, whitish, occasionally interspersed with lamellulae. Stipe subcylindrical, whitish, covered with minute squamules. Context composed of an ixoplect with clamp connections on hyphal septa. Basidiospores subglobose to broadly ellipsoid, thin-walled, smooth, nearly colorless, inamyloid. Basidia clavate, two-, three- or four-spored, nearly colorless. Lamella trama subregular. Hymenial cystidia absent. Pileipellis generally composed of a rectocutis, but locally issuing clusters of hyphae and thus locally rendering a tomentum to plagiotrichoderm. Stipitipellis generally composed of a rectocutis, but locally issuing clusters of hyphae or inflated cells and thus locally rendering a tomentum to plagiotrichoderm.

Notes: This is a monotypic genus sister to *Delicatula* (Figure 11). See Section 1.2.1.1.7.2.1 for details.

(1.2.1.1.7.2.1) *Xuaniella urbica* Kun L. Yang, Jia Y. Lin & Zhu L. Yang, sp. nov. (Figure 18 and Table 10).

Registration identifier: FN572642.

Etymology: The first known discovery of this fungus is by Kun L. Yang on a planted *Zoysia* lawn in the new urban district of Guangzhou City ten years ago (2015). Until now, this fungus has never been recorded in a natural habitat (see also Notes).

Type: China. Guangdong Province: Foshan City, Nanhai District, Qiandenghu Lake Park, 23°03′18″ N, 113°08′26″ E, elevation 1 m, 11 May 2025, Jia Y. Lin & Kun L. Yang, K25033 (HKAS150761, holotype; HTBM2885, isotype).

Description: Basidiomata tiny to small, without a distinct color change after damage; odor indistinct; taste indistinct. Pileus 6–35 mm in diameter, paraboloid to campanulate at first, becoming plano-convex to applanate, more or less umbonate or papillary at center, pure white (#FFFFFF), moon yellow (#FAF9AA), beach yellow (#F9F3C9), cream orange (#FEF3CE), to milky yellow (#FFF07A) at background, covered with pruinose, floccose to fibrous, concolorous or bright teak brown (#AF9C6D), mire brown (#B89F89) to dirty brass brown (#B47F5A) squamules. Lamellae sinuate to decurrent, subdistant, pure white (#FFFFFF) to alabaster white (#F9F9F9), with a more or less undulate edge, occasionally interspersed with lamellulae. Stipe 19–52 mm long, 1.5–4 mm thick, subcylindrical, more or less curved, subtransparently whitish, covered with pruinose, alabaster white (#F9F9F9) squamules. Context composed of a seeming not sarcodimitic, not or slightly cyanophilic ixoplect with abundant clamp connections on hyphal septa, with slender thromboplerous hyphae containing oil-like deuteroplasm frequently present. Basidiospores {40/6/4} (5.5) 6–6.5 (7) [6.30 ± 0.31, 6.50] × 5–6 [5.29 ± 0.33, 5.00] µm, *Q* = 1.08–1.30 [1.19 ± 0.08, 1.20], subglobose to broadly ellipsoid, thin-walled, smooth, nearly colorless to slightly yellowish, inamyloid, slightly to strongly cyanophilic, frequently containing granular globular contents, with a small apiculus. Basidia 20–29 × 5.5–6.5 μm, clavate, two-, three- or four-spored, thin-walled, nearly colorless, surrounding by basidioles sized 16.5–28 × 3.5–6.5 μm. Lamella trama subregular, composed of 3–22 µm wide, thin-walled to slightly thick-walled, yellowish, compact, rarely to moderately branching hyphae. Hymenial cystidia not recognized. Pileipellis generally composed of a rectocutis by thin-walled, 3.5–6.5 µm wide, nearly colorless, moderately to frequently branching hyphae, but locally issuing oblique to erect clusters of thin-walled to slightly thick-walled, 3.5–10 µm wide, nearly colorless to brownish, moderately branching hyphae, and thus locally rendering a tomentum to plagiotrichoderm. Stipitipellis generally composed of a rectocutis by thin-walled, 2.5–5.5 µm wide, nearly colorless, rarely branching hyphae, but locally issuing oblique clusters of thin-walled to slightly thick-walled, 4–16 µm wide, nearly colorless, moderately to frequently branching hyphae or irregularly clavate inflated cells, and thus locally rendering a tomentum to plagiotrichoderm.

Habit and distribution: Usually gregarious, occasionally solitary, saprotrophic on planted *Zoysia* lawns or gardens (especially those with bamboos) in cities. Currently known from East and South China.

Other collections examined: China. Guangdong Province: Guangzhou City, Baiyun District, Guangzhou Children’s Park, 23°11′11″ N, 113°15′53″ E, elevation 60 m, 2 April 2023, Kun L. Yang & Jia Y. Lin, K23013 (HTBM0528). Guangdong Province: Guangzhou City, Haizhu District, Haizhuhu Lake Park, 23°04′31″ N, 113°19′20″ E, elevation 2 m, 21 October 2023, Jia Y. Lin & Kun L. Yang, L23470 (HTBM1451). Guangdong Province: Guangzhou City, Nansha District, South Waterfront Cape (Nanbin Shuijiao) Park, 22°46′48″ N, 113°30′51″ E, elevation 1 m, 27 April 2024, Kun L. Yang & Jia Y. Lin, L24109 (HTBM1996). Guangdong Province: Foshan City, Nanhai District, Qiandenghu Lake Park, 23°03′18″ N, 113°08′26″ E, elevation 1 m, 11 May 2025, Jia Y. Lin & Kun L. Yang, K25031 (HTBM2883), K25032 (HTBM2884), K25044 (HTBM2896) and K25045 (HTBM2897). Guangdong Province: Guangzhou City, Huangpu District, Huangpu Library, 23°10′39″ N, 113°29′44″ E, elevation 150 m, 20 May 2025, Jia Y. Lin & Kun L. Yang, L25254 (HTBM2536). Guangdong Province: Guangzhou City, Tianhe District, South China Botanical Garden, 23°11′22″ N, 113°21′32″ E, elevation 50 m, 24 May 2025, Kun L. Yang & Jia Y. Lin, L25267 (HTBM2549) and L25268 (HTBM2550). Guangdong Province: Guangzhou City, Liwan District, Liwanhu Lake Park, 23°07′21″ N, 113°13′36″ E, elevation 2 m, 1 June 2025, Jia Y. Lin & Kun L. Yang, L25304 (HTBM2586) and L25305 (HTBM2587). Guangdong Province: Guangzhou City, Haizhu District, Guangzhou International Bio-Island, 23°04′03″ N, 113°22′19″ E, elevation 5 m, 2 June 2025, Jia Y. Lin & Kun L. Yang, L25324 (HTBM2606). Guangdong Province: Guangzhou City, Tianhe and Haizhu Districts, Guangzhou Opera House, Haixinsha Island and Haixin Bridge, 23°06′46″ N, 113°19′00″ E, elevation 2 m, 3 June 2025, Kun L. Yang & Jia Y. Lin, L25328 (HTBM2610), L25330 (HTBM2612), L25331 (HTBM2613), L25332 (HTBM2614), L25334 (HTBM2616), K25058 (HTBM2910) and K25059 (HTBM2911). Guangdong Province: Guangzhou City, Haizhu District, Wanshengwei, 23°06′02″ N, 113°22′45″ E, elevation 5 m, 19 June 2025, Kun L. Yang & Jia Y. Lin, L25404 (HTBM2686). Guangdong Province: Guangzhou City, Huangpu District, Xiangxue Park, 23°11′02″ N, 113°31′08″ E, elevation 170 m, 20 June 2025, Kun L. Yang & Jia Y. Lin, L25415 (HTBM2697). Guangdong Province: Foshan City, Nanhai District, Wenhanhu Lake Park, 23°01′03″ N, 113°13′52″ E, elevation 3 m, 21 June 2025, Kun L. Yang & Jia Y. Lin, L25424 (HTBM2706), L25434 (HTBM2716), L25437 (HTBM2719) and L25439 (HTBM2721).

Notes: This fungus is very common in urban areas across East and South China, mostly found on lawns made of *Zoysia* plants, and sometimes also on ground in gardens (especially those with bamboos). Based on available records, this fungus, although common, has neither been found in a natural habitat nor recorded outside China. However, as inferred by its preference for growing on *Zoysia* lawns and the fact that some *Zoysia* species are native to China [115], a natural meadow with *Zoysia* could be a natural habitat for this fungus, and this fungus is also probably native to China.

Based on our observations, basidiomata of this fungus growing in habitats exposed to strong light often exhibit lighter colors (whitish to yellowish), while those in darker habitats often exhibit darker colors (brownish). This is contrary to some common experience on pigments.

This fungus is possibly poisonous. According to an Internet communication from 26 May 2025, a child from Guangzhou City (Guangdong Province) suffered stomachache and vomiting after eating this fungus.

**Figure 19:**
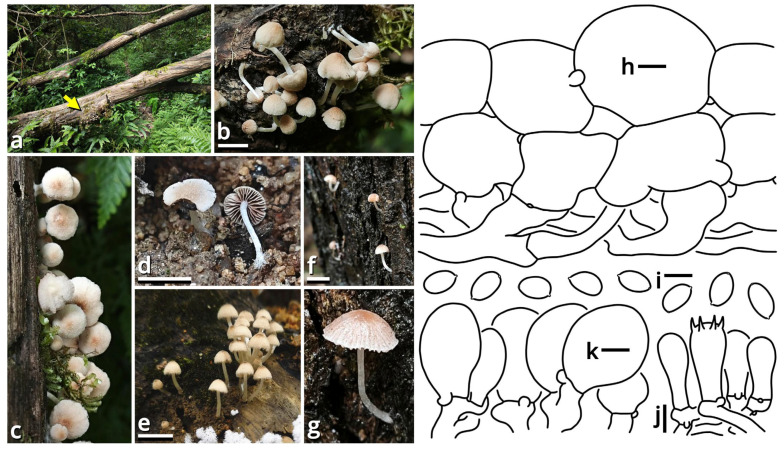
*Candolleomyces striginus* (photos by Kun L. Yang, Jia Y. Lin & Zhen-Chao Liu; drawings by Kun L. Yang from HKAS150762 (holotype)). (**a**) Habitat; the yellow arrow indicates a group of basidiomata for HKAS150762 (holotype). (**b**–**g**) Basidiomata ((**b**,**c**) HKAS150762 (holotype); (**d**) HTBM0800; (**e**) HTBM1548; (**f**,**g**) HTBM1399). (**h**) Pileipellis. (**i**) Basidiospores. (**j**) Hymenium, subhymenium and lamella trama. (**k**) Cheilocystidia. Bars: (**b**,**e**,**f**) = 1 cm, (**d**) = 5 mm, (**h**–**k**) = 5 µm.

Some collections cited under the name *Atheniella flavoalba* (≡*Mycena flavoalba*) in previous studies (e.g., Ge et al. 2021 [110]), if without hymenial cystidia, may now represent this species.

(1.2.1.1.8) Psathyrellaceae Vilgalys, Moncalvo and Redhead.

(1.2.1.1.8.1) *Candolleomyces* D. Wächt. & A. Melzer.

(1.2.1.1.8.1.1) *Candolleomyces striginus* Kun L. Yang, Jia Y. Lin, Zhen-Chao Liu & Zhu L. Yang, sp. nov. (Figure 19 and Table 10).

Registration identifier: FN572644.

Etymology: Referring to its brownish color and arboreal (lignicolous) habit like owls (Strigiformes).

Type: China. Guangdong Province: Shaoguan City, Shixing County, Chebaling Mountains, 24°42′02″ N, 114°11′27″ E, elevation 550 m, 14 July 2025, Kun L. Yang & Jia Y. Lin, K25099 (HKAS150762, holotype; HTBM2951, isotype).

Diagnosis: Differing from other species in *Candolleomyces candolleanus* complex by the tiny to small basidiomata with a lignicolous habit.

Description: Basidiomata tiny to small, without a distinct color change after damage; odor indistinct; taste indistinct. Pileus 4–23 mm in diameter, hemispherical, convex, paraboloid, plano-convex to applanate, hygrophanous, merino white (#F9F5EC), lotus-root orange (#F5E9D9), muddy brown (#BD936D) to brass brown (#B3794F), becoming lighter towards margin, becoming pale after drying, covered with fibrous, pure white (#FFFFFF) veil remnants. Lamellae adnexed, adnate to sinuate, subdistant, nearly crowded to crowded, merino white (#F9F5EC), lotus-root orange (#F5E9D9) to dull beaver brown (#92745E), with an entire, slightly undulate to slightly serrate, pale edge, interspersed with abundant lamellulae. Stipe 5–22 mm long, 0.5–2 mm thick, subcylindrical, often slightly tapering upwards, more or less curved, subtransparently whitish, slightly longitudinally striate, covered with furfuraceous to fibrous, opaquely whitish squamules. Basidiospores {40/3/2} 5.5–7 [6.20 ± 0.46, 6.50] × 3–4 (4.5) [3.63 ± 0.33, 3.50] µm, *Q* = 1.50–2.00 [1.71 ± 0.15, 1.86], oblong, thin-walled to slightly thick-walled, smooth, brownish, with an indistinct to distinct germ pore, with a tiny apiculus. Basidia 12–20 × 5.5–7 μm, clavate, two- or four-spored, thin-walled, nearly colorless. Lamella trama regular to subregular, composed of 2.5–13 µm wide, thin-walled, nearly colorless, compact, moderately branching hyphae. Cheilocystidia abundant, 8–22 × 8–13 µm, broadly clavate, thin-walled to slightly thick-walled, smooth, nearly colorless. Pleurocystidia absent. Pileipellis composed of an epidermoid cutis to paraderm by usually two layers of inflated cells sized 19–48 × 8–35 µm, broadly ellipsoid to elongate or clavate, thin-walled to slightly thick-walled, nearly colorless to brownish. Clamp connections present.

Habit and distribution: Gregarious, saprotrophic on deadwood or wood chips in subtropical forests. Currently known from South China.

Other collections examined: China. Guangdong Province: Guangzhou City, Tianhe District, Furnace Mountain (Huolushan) Forest Park, 23°11′07″ N, 113°23′05″ E, elevation 200 m, 13 June 2023, Kun L. Yang, K23285 (HTBM0800). Guangdong Province: Guangzhou City, Huangpu District, Jiangdong Village, Boluoshan Hill, 23°11′36″ N, 113°32′25″ E, elevation 150 m, 16 August 2023, Zhen-Chao Liu, Jia Y. Lin & Kun L. Yang, S23437 (HTBM1548). Guangdong Province: Guangzhou City, Tianhe District, South China Agricultural University, Arboretum, 23°09′27″ N, 113°21′22″ E, elevation 40 m, 12 September 2023, Jia Y. Lin, L23418 (HTBM1399).

Notes: This species had been previously recognized as new in the *Candolleomyces candolleanus* complex by Yang et al. (2025) [116] with three sequenced collections (HTBM0800, HTBM1399 and HTBM1548). Now one more collections with abundant basidiomata (HKAS150762 (holotype)) confirmed its unique position both morphologically (see Diagnosis) and molecularly (Table 3), and thus it is formally proposed here. For details on its phylogeny and the surrounding species complex, see Yang et al. (2025) [116].

**Figure 20:**
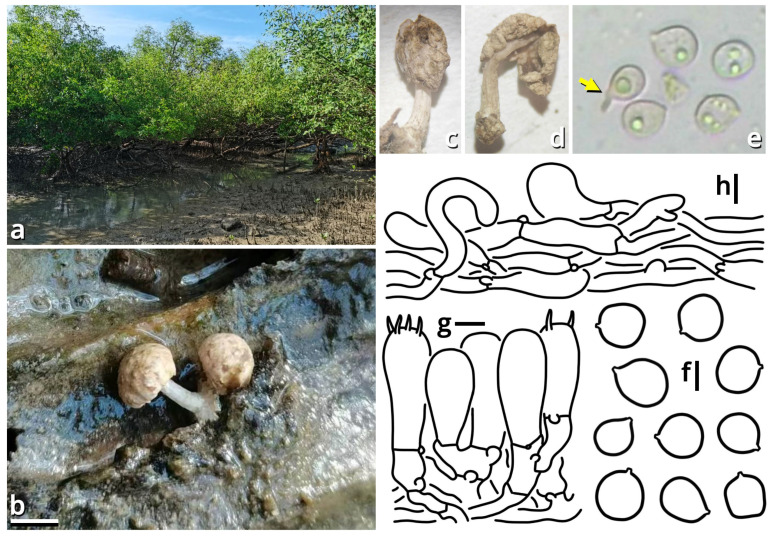
*Candolleomyces vagabundoides* (photos by Gu Miao & Kun L. Yang; drawings by Kun L. Yang from HKAS150768 (holotype)). (**a**) Habitat, an intertidal mangrove dominated by *Sonneratia apetala*. (**b**–**d**) Basidiomata of HKAS150768 (holotype) ((**b**) in situ; (**c**,**d**) under SM). (**e**,**f**) Basidiospores of HKAS150768 (holotype) ((**e**) under LM, the yellow arrow indicating a basidiospore with an apiculus protruding outwards the tip of a sterigma fragment, a characteristic for ballistospores; (**f**) line drawing). (**g**) Hymenium, subhymenium and lamella trama. (**h**) Pileipellis. Bars: (**b**) = 5 mm, (**f**–**h**) = 5 µm.

(1.2.1.1.8.1.2) *Candolleomyces vagabundoides* Kun L. Yang, Jia Y. Lin, Gu Miao & Zhu L. Yang, sp. nov. (Figure 20 and Table 10).

Registration identifier: FN573001.

Etymology: Referring to its similarity and close relationship to *Candolleomyces albovagabundus* and *C. brunneovagabundus*.

Type: China. Fujian Province: Xiamen City, Xiang’an District, Xiatanwei Coastal Wetland Park, exact location unknown, elevation 0 m, 29 June 2025, Gu Miao, Z. H. Zhang, Kun L. Yang & Jia Y. Lin, S25043 (HKAS150768, holotype; HTBM3032, isotype).

Diagnosis: Differing from *Candolleomyces albovagabundus* and *C. brunneovagabundus* by a better developed stipe, and basidiospores with thinner wall plus rounder in outline (*Q* = 1.00–1.18 (1.42)).

Description: Basidiomata tiny, germinating from the top of the substrate, without a distinct color change after damage; odor indistinct; taste unknown. Pileus 6–7 mm in diameter, hemispherical to convex, lotus-root orange (#F5E9D9), bone brown (#E3D3C4) to mist brown (#DCD8C9) at background, covered with flaky to felty, nearly concolorous squamules. Hymenophore sublamellate, bone brown (#E3D3C4) to thatch red (#BEA39C). Stipe 7–8 mm long, 1 mm thick, slightly tapering upwards, subtransparently whitish, longitudinally striate. Basidiospores {40/2/1} 6–8 (8.5) [7.01 ± 0.59, 6.50] × (5.5) 6–7 (7.5) [6.35 ± 0.44, 6.50] µm, *Q* = 1.00–1.18 (1.42) [1.11 ± 0.08, 1.15], often somewhat irregularly shaped, nearly subglobose to broadly ellipsoid, thin-walled to slightly thick-walled, smooth, nearly colorless to slightly brownish, inamyloid, frequently containing granular globular contents, with a small apiculus. Basidia 11.5–16 × 6–7 μm, clavate, one-, two-, three- or four-spored, thin-walled, nearly colorless, surrounding by basidioles sized 11–15 × 6–7 μm. Hymenophore trama subregular, composed of 2.5–6.5 µm wide, thin-walled to slightly thick-walled, nearly colorless, compact, moderately branching hyphae. Hymenial cystidia not recognized. Pileipellis generally composed of a rectocutis to clavicutis by abundant filamentous and often flexuous hyphae 2.5–7 µm wide, thin-walled, nearly colorless to slightly brownish, moderately compact to compact, moderately to frequently branching, and some inflated cells sized 15–32 × 4–15 µm, elongate or clavate or irregularly shaped, interjacent or terminal, thin-walled to slightly thick-walled, nearly colorless to brownish. Pileus trama radially subregular, composed of a thigmoplect to aeroplect by moderately abundant to abundant filamentous hyphae 2.5–7.5 µm wide, thin-walled, nearly colorless, moderately compact to compact, moderately to frequently branching, and abundant inflated cells sized 20–75 × 8–30 µm, ellipsoid to elongate or clavate or irregularly shaped, interjacent or terminal, thin-walled to slightly thick-walled, nearly colorless. Clamp connections present.

Habit and distribution: Gregarious, saprotrophic on deadwood of *Sonneratia apetala* in intertidal mangroves. Currently only known from the type locality (Fujian Province, China).

Notes: Two unique species of marine mushrooms, *Candolleomyces albovagabundus* and *C. brunneovagabundus*, were discovered from *Sonneratia* mangroves in Guangdong Province of China [117]. They exhibit features well-adapted to marine environment, producing sequestrate but still agaricoid basidiomata with a detachable pileus functioned as a driftable dissemination unit, thick-walled basidiospores and better viability in seawater culture [117]. They also form a distinct lineage within the genus *Candolleomyces*, with the relationship to other known species of *Candolleomyces* poorly resolved [116,117]. One more species recently discovered in Fujian Province of China is here added to this special lineage with support from sequences shown in Table 3. It looks similar to *C. albovagabundus* and *C. brunneovagabundus* both morphologically and ecologically but has better developed stipe, seeming more exposed hymenophore, basidiospores with a thinner wall and even seeming with a ballistic potential (Figure 20), and thus may be an earlier diverged transition species between terrestrial and marine *Candolleomyces*. Whether its pileus is also naturally detachable remains uncertain, but it has more abundant filamentous hyphae and fewer inflated cells in the pileus–stipe transition area that may support a stronger connection between pileus and stipe.

**Figure 21:**
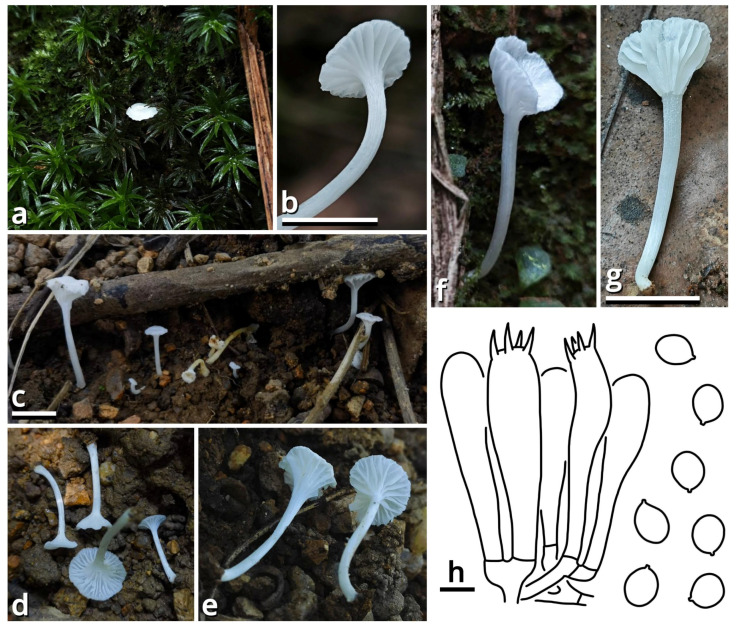
*Gugumyces columbarius* (photos by Zhen-Chao Liu, Kun L. Yang & Jia Y. Lin; drawings by Kun L. Yang from HKAS150763 (holotype)). (**a**–**g**) Basidiomata and their habitats ((**a**,**b**) HTBM2032; (**c**,**e**) HKAS150763 (holotype); (**d**) HTBM1559; (**f**,**g**) HTBM2166). (**h**) Hymenium and basidiospores. Bars: (**b**,**c**,**g**) = 1 cm, (**h**) = 10 µm.

(1.2.1.1.9) Incertae sedis.

(1.2.1.1.9.1) *Gugumyces* Kun L. Yang, Jia Y. Lin, Zhen-Chao Liu, Yu-Rong Liang & Zhu L. Yang, gen. nov. (Figure 21 and Table 10).

Registration identifier: FN573003.

Type: *Gugumyces columbarius* Kun L. Yang, Jia Y. Lin, Zhen-Chao Liu, Yu-Rong Liang & Zhu L. Yang (see Section 1.2.1.1.9.1.1).

Etymology: *Gugu*, pronunciation of a Chinese cyberword meaning pigeons and delay, referring to the silky whitish morphology like a pigeon’s plumage of its type species and the background that Kun L. Yang discovered its type species nine years ago (2016), but it has taken until now to obtain enough collections to enable a formal description.

Diagnosis: Differing from other genera of Agaricales by tiny to small, whitish, omphalioid basidiomata with silky pileus and stipe, sometimes forked lamellae, broadly ellipsoid to ellipsoid, thin-walled, smooth and inamyloid basidiospores, absent or indistinguishable hymenial cystidia, pileipellis and stipitipellis composed of a rectocutis and distinct ITS and nrLSU sequences.

Description: Basidiomata tiny to small, omphalioid, without a distinct color change after damage; odor indistinct; taste unknown. Pileus depressed to umbilicate at center, pale, silky. Lamellae decurrent, subdistant, pale, with a smooth to somewhat undulate edge, sometimes forked, interspersed with lamellulae. Stipe pale, silky, subcylindrical or tapering downwards, more or less curved, sometimes slightly bulbous at base. Context composed of a holomonomitic thigmoplect to ixoplect, without clamp connections on hyphal septa. Basidiospores broadly ellipsoid to ellipsoid, thin-walled, smooth, nearly colorless, inamyloid, with a small apiculus. Basidia clavate, two-, three- or four-spored, thin-walled, nearly colorless. Lamella trama slightly bilateral to subregular. Cheilocystidia absent or indistinguishable from larger basidioles. Pleurocystidia absent or indistinguishable from larger basidioles. Pileipellis composed of a rectocutis. Stipitipellis similar to pileipellis.

Notes: This genus currently comprises only a new species described below. We have performed Sanger sequencing on the *rpb2* and *tef-1α* loci of all collections of this species, but all received multipeaked results. In a two-locus (5.8S-nrLSU) phylogeny based on alignment in Vizzini et al. (2024) [83], this genus seems to nest in Callistosporiaceae but requires further confirmation.

(1.2.1.1.9.1.1) *Gugumyces columbarius* Kun L. Yang, Jia Y. Lin, Zhen-Chao Liu, Yu-Rong Liang & Zhu L. Yang, sp. nov. (Figure 21 and Table 10).

Registration identifier: FN573002.

Etymology: Referring to the silky whitish morphology like a pigeon’s plumage.

Type: China. Guangdong Province: Guangzhou City, Huangpu District, Jiangdong Village, Boluoshan Hill, 23°11′36″ N, 113°32′25″ E, elevation 150 m, 26 July 2023, Zhen-Chao Liu, Jia Y. Lin & Kun L. Yang, S23331 (HKAS150763, holotype; HTBM1042, isotype).

Description: Basidiomata tiny to small, omphalioid, without a distinct color change after damage; odor indistinct; taste unknown. Pileus 3–10.5 mm in diameter, silky, depressed to umbilicate at center, pure white (#FFFFFF) to zircon white (#F3F5FD). Lamellae decurrent, subdistant, concolorous with pileus, with a smooth to somewhat undulate edge, sometimes forked, interspersed with lamellulae. Stipe 11–31 mm long, 1–1.5 mm thick, silky, subcylindrical or tapering downwards, more or less curved, sometimes slightly bulbous at base, concolorous with pileus. Context composed of a holomonomitic thigmoplect to ixoplect, without clamp connections on hyphal septa. Basidiospores {40/2/2} (9) 9.5–10.5 (11.5) [9.85 ± 0.53, 9.50] × (6.5) 7–8.5 [7.58 ± 0.53, 7.50] µm, *Q* = 1.13–1.50 [1.31 ± 0.10, 1.27], mostly broadly ellipsoid to ellipsoid, rarely subglobose, thin-walled, smooth, nearly colorless, inamyloid, frequently containing globular contents, with a small apiculus. Basidia 40–52 × 10–14 μm, clavate, two-, three- or four-spored, thin-walled, nearly colorless. Lamella trama slightly bilateral to subregular, composed of 3–8 µm wide, thin-walled to slightly thick-walled, nearly colorless, compact, moderately to frequently branching hyphae. Cheilocystidia absent or indistinguishable from larger basidioles. Pleurocystidia absent or indistinguishable from larger basidioles. Pileipellis composed of a rectocutis by thin-walled to slightly thick-walled, 5–14 µm wide, nearly colorless, rarely to moderately branching hyphae. Stipitipellis similar to pileipellis.

Habit and distribution: Usually gregarious, occasionally solitary, saprotrophic on soil in subtropical forests. Currently known from South China.

Other collections examined: China. Guangdong Province: Guangzhou City, exact location unknown, 21 July 2023, Yu-Rong Liang, S23317-ACE84 (HTBM1028). Guangdong Province: Guangzhou City, Huangpu District, Jiangdong Village, Boluoshan Hill, 23°11′36″ N, 113°32′25″ E, elevation 150 m, 17 August 2023, Zhen-Chao Liu, Jia Y. Lin & Kun L. Yang, S23448 (HTBM1559). Guangdong Province: Huizhou City, Longmen County, Youtian Forest Farm, 23°38′49″ N 114°02′48″ E, elevation 100 m, 6 July 2024, Kun L. Yang, K24042 (HTBM2032). Guangdong Province: Guangzhou City, Baiyun District, Baiyun Mountain Scenic Area, 23°12′26″ N, 113°18′04″ E, elevation 100 m, 31 July 2024, Jia Y. Lin, L24181 (HTBM2166).

(1.2.1.1.9.2) *Pruinomycena* Kun L. Yang, Jia Y. Lin & Zhu L. Yang, gen. nov.

Registration identifier: FN572626.

Type: *Pruinomycena subcyanocephala* (W.N. Chou) Kun L. Yang, Jia Y. Lin & Zhu L. Yang (see Section 1.2.1.1.9.2.1).

Etymology: Referring to the pruinose to pubescent basidiomata of this group.

Diagnosis: Differing from *Hemimycena* and *Mycenella* by the vividly colored basidiomata and the pileipellis composed of a surface layer of branched filamentous hyphae lying on a coniodermal (pseudoparenchymatous) mass of inflated cells.

Description: Basidiomata tiny, without a distinct color change after damage. Pileus hemispherical, paraboloid, campanulate to plano-convex, pruinose to pubescent, vividly colored, indistinctly striate. Lamellae adnate, emarginate, adnexed to free, sometimes with decurrent tooth, subdistant to nearly crowded, pale, with a more or less serrate to pruinose edge, interspersed with abundant lamellulae, slightly interveined. Stipe subcylindrical, pruinose to pubescent, whitish or with a tinge of pileus color, more or less hairy at base. Context composed of a thigmoplect to ixoplect, often with clamp connections on hyphal septa. Basidiospores more or less ellipsoid, smooth, nearly colorless, inamyloid to amyloid. Basidia clavate, thin-walled, nearly colorless. Lamella trama subregular. Cheilocystidia present. Pleurocystidia present or possibly sometimes absent. Pileipellis composed of a surface layer of branched filamentous hyphae, lying on a coniodermal (pseudoparenchymatous) mass of subglobose to polygonal inflated cells. Stipitipellis similar to the surface layer of pileipellis.

Notes: This genus corresponds to *Mycena* sect. *Aciculae*, a section recently accepted with only its type species *M. acicula*, but is now excluded from Mycenaceae. It is recognized with two species in a strongly supported clade near *Hemimycena* and *Mycenella* (Figure 14). Its morphological concept is generally considered with studies of Singer (1962, 1986) [78,91], Maas Geesteranus (1992) [79], Chang & Chou (2019) [118] and Bau et al. (2021) [80].

Singer (1986) [78] mentioned some other possible species of *Mycena* sect. *Aciculae* like *M. oregonensis* and *M. xanthopoda*, but more sequence data are required for confirmation, due to their similarity with *Atheniella* and *Phloeomana* (Ge et al. 2021 [110], Harder et al. 2023 [119]).

(1.2.1.1.9.2.1) *Pruinomycena subcyanocephala* (W.N. Chou) Kun L. Yang, Jia Y. Lin & Zhu L. Yang, comb. nov.

Registration identifier: FN572627.

Basionym: *Mycena subcyanocephala* W.N. Chou, *Fungal Science, Taipei* 34(1): 13 (2019).

Notes: This species is described from Taiwan, an island in Southeast China [118]. Our present phylogenetic analysis on its holotype TNM F0032108 (ITS sequence available from MK400426) strongly supports its close relationship with “*Mycena acicula*” (Figure 14). Morphology does also corroborate this inference that the two species share a special pileipellis composed of a surface layer of branched filamentous hyphae lying on a coniodermal (pseudoparenchymatous) mass of inflated cells.

(1.2.1.1.9.2.2) *Pruinomycena acicula* (Schaeff.) Kun L. Yang, Jia Y. Lin & Zhu L. Yang, comb. nov.

Registration identifier: FN572628.

**Figure 22:**
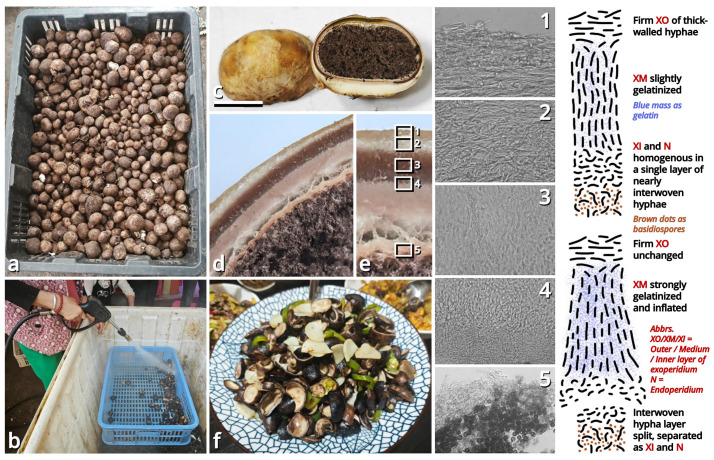
*Astraeusmaculatus* rediscovered together with *A. ryoocheoninii*, and the inferred mechanism for basidioma dehiscence based on microscopic observations of *A. maculatus* (**1**–**5**) (photos and drawings by Kun L. Yang). (**a**) A basket of *Astraeus* sold at a market, in which collections of *A. maculatus* and *A. ryoocheoninii* were found. (**b**) Skilled workers use a high-pressure water gun to wash off the soil on *Astraeus* basidiomata. (**c**) Epitype of *A. maculatus* (HKAS150766). (**d**,**e**) Radial sections of the peridium of *A. maculatus* (HKAS150766). (**f**) Tasty cuisine made with *A. maculatus* and *A. ryoocheoninii*. Bar: (**c**) = 1 cm.

Basionym: *Agaricus acicula* Schaeff., *Fungorum qui in Bavaria et Palatinatu circa Ratisbonam nascuntur Icones (Ratisbonae)* 4: 52 (1774) (≡*Mycena acicula* (Schaeff.) P. Kumm., *Der Führer in die Pilzkunde (Zerbst)*: 109 (1871)).

(1.2.1.2) Boletales E.-J. Gilbert.

(1.2.1.2.1) Diplocystidiaceae Kreisel.

(1.2.1.2.1.1) *Astraeus* Morgan.

(1.2.1.2.1.1.1) *Astraeus maculatus* (Pat.) Kun L. Yang, Jia Y. Lin & Zhu L. Yang, comb. nov. (Figure 22 and Table 10).

Registration identifier: FN572624.

Basionym: *Phlyctospora maculata* Pat., *Bulletin de la Société Mycologique de France* 8(4): 189 (1892).

Holotype examined (containing one and a half basidiomata and a piece of peridium): China. Chongqing Municipality: Chengkou County, exact location and date unknown, seeming hypogeous, provided by M. Farges, FH-Pat.-Herb. Sheet 1382, preserved in Harvard University Herbaria, USA (Patouillard (1892): “Chine, Su-Tchuen Oriental, district de Tchen-Keoutin; vraisemblablement hypogé”).

Epitype designated here (registration identifier: FN573004): China. Yunnan Province: Kunming City, Guandu District, Mushuihua Wild Mushroom Trading Center, 25°00′32″ N, 102°43′38″ E, elevation 1900 m, 19 July 2025, Kun L. Yang & Jia Y. Lin, purchased from a trader: K25228 (HKAS150766, epitype; HTBM3130, isoepitype).

Description: Basidiomata small, 10–30 mm in diameter, 10–20 mm in height, oblately subglobose, sessile; completely dehiscent state not observed; odor pleasant like taros; taste sweet and umami. Exoperidium leathery, 1–2 mm thick, composed of an outer layer rendering a felty basidioma surface in thatch yellow (#F1ECC5), a medium layer in coffee red (#7B5B4E), and an inner layer in pale meat brown (#E4C8BB). Endoperidium very thin, colored similar to outer and inner layers of exoperidium, opened at top. Gleba pulverulent, wood-ash brown (#A0968A). Basidiospores with echinulate ornamentation, {10/1/1} 8.5–11 [10.25 ± 0.81, 10.50] × 8–11 [9.80 ± 0.87, 10.50] µm, *Q* = 1.00–1.11 [1.05 ± 0.04, 1.00] including ornamentation and {10/1/1} 6.5–9 [7.95 ± 0.76, 8.00] × 6–9 [7.70 ± 0.81, 7.50] µm, *Q* = 1.00–1.08 [1.03 ± 0.03, 1.00] excluding ornamentation in holotype, {40/2/2} 8–11 [9.58 ± 0.85, 10.00] µm in diameter including ornamentation 0.5–1.5 µm high in other collections, mostly globose, rarely subglobose, thick-walled, brownish, often surrounded by remnants of 2–5 μm wide placental hyphae (“capillitia”). Outer layer of exoperidium composed of 2–5 μm wide, thick-walled, yellowish or nearly colorless, compact, moderately to frequently branching, bidirectionally regularly to subregularly arranged hyphae with clamp connections. Medium layer of exoperidium gelatinized, composed of 3–6 μm wide, slightly thick-walled to thick-walled, yellowish, compact, moderately branching, clamped hyphae arranged as perpendicular to the outer and inner layer. Inner layer of exoperidium composed of 2.5–6 μm wide, slightly thick-walled to thick-walled, yellowish or nearly colorless, compact, moderately to frequently branching, interwoven hyphae with clamp connections. Endoperidium composed of 2.5–5.5 μm wide, slightly thick-walled to thick-walled, yellowish or nearly colorless, compact, moderately to frequently branching, subregularly arranged to interwoven hyphae with clamp connections.

Habit and distribution: Gregarious on soil in subtropical to temperate forests. Currently known from Southwest China.

Other collections examined: China. Yunnan Province: Kunming City, Guandu District, Mushuihua Wild Mushroom Trading Center, 25°00′32″ N, 102°43′38″ E, elevation 1900 m, 19 July 2025, Kun L. Yang & Jia Y. Lin, purchased from a trader: K25229 (HTBM3131). Collections of *Astraeus ryoocheoninii* examined for comparison: China. Yunnan Province: Kunming City, Guandu District, Mushuihua Wild Mushroom Trading Center, 25°00′32″ N, 102°43′38″ E, elevation 1900 m, 19 July 2025, Kun L. Yang & Jia Y. Lin, purchased from a trader: L25560 (HTBM2842), K25225 (HTBM3127), K25226 (HTBM3128) and K25227 (HTBM3129).

Notes: Based on the position of type species, the genus *Phlyctospora* is now synonymized with *Scleroderma* [120]. *Phlyctospora maculata* was described by Patouillard (1892) [121] with only a holotype collection from Southwest China, and Guzmán (1970) [122] treated it as a later synonym of *Astraeus hygrometricus*. In 2006, Zhu L. Yang re-examined the holotype of *P. maculata*, consistently found it actually to be an *Astraeus* species like *A. hygrometricus* in an indehiscent state.

In 2025, in a market 900 km away from the type locality of *Phlyctospora maculata*, Kun L. Yang and Jia Y. Lin noticed a basket of *Astraeus* basidiomata sold as wild vegetables, which were expensively priced as CNY 100–110/kg, called “Yang Yan Jing Jun” (goat-eye mushrooms) by local people. Kun L. Yang and Jia Y. Lin bought some for dinner and taxonomic studies, and finally identified two species by six collections isolated from them through morphology and phylogeny: (1) A species clustered with KFI-DMZ002 (holotype of *A. ryoocheoninii*) as a single species in phylogeny, having basidiospores {40/2/2} 8–9.5 (10) [8.89 ± 0.59, 9.00] µm in diameter including ornamentation, generally consistent with the protologue of *A. ryoocheoninii* [123]; and (2) a species without relatives in the *A. koreanus–telleriae–smithii* complex in phylogeny (referred to Phosri et al. (2007, 2013) [124,125]), having basidiospores {40/2/2} 8–11 [9.58 ± 0.85, 10.00] µm in diameter including ornamentation, generally consistent with the observation for holotype of *Phlyctospora maculata* by Zhu L. Yang. Therefore, the species of (2) was recognized as *Phlyctospora maculata*, recombined as *Astraeus maculatus* here, with a collection designated as epitype to better introduce its concept.

**Figure 23:**
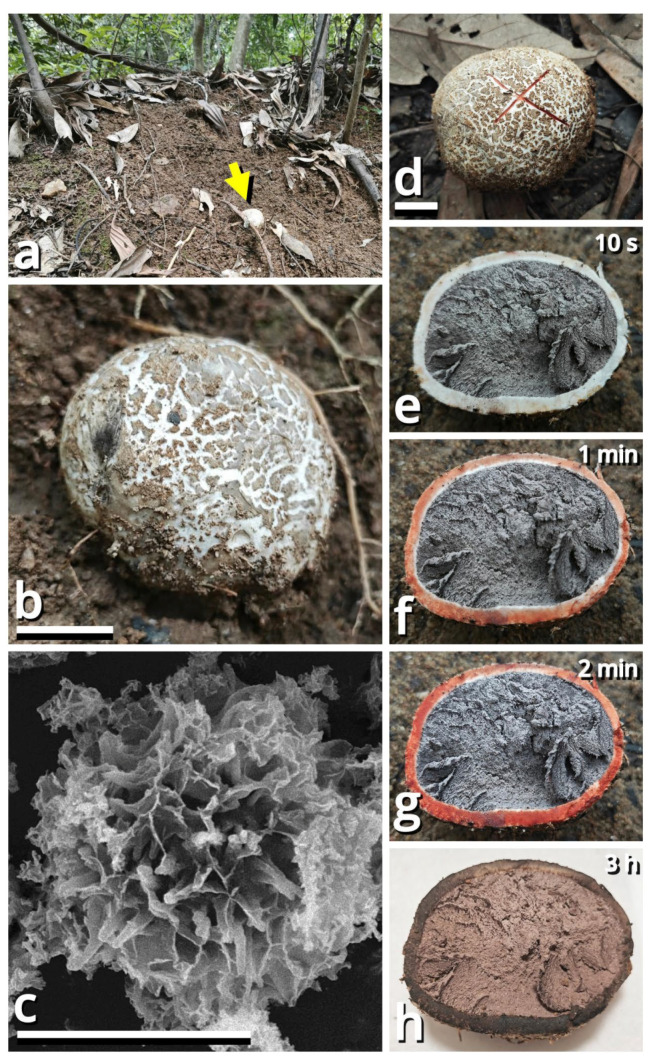
*Scleroderma cruentatum* (photos by Kun L. Yang & Jia Y. Lin). (**a**) Habitat, with a yellow arrow pointing a basidioma. (**b**) A basidioma of HTBM2663. (**c**) A basidiospore from HTBM3023. (**d**–**f**) A basidioma of HKAS150765 (holotype), showing the reddening coloration after cutting ((**e**) 10 s after cutting; (**f**) 1 min after cutting; (**g**) 2 min after cutting; (**h**) 3 h after cutting). Bars: (**b**,**d**) = 1 cm, (**c**) = 5 µm.

The mechanism for basidioma dehiscence of *Astraeus maculatus* was inferred based on microscopic observations on its peridium structure (Figure 22).

(1.2.1.2.2) Sclerodermataceae Corda.

(1.2.1.2.2.1) *Scleroderma* Pers.

(1.2.1.2.2.1.1) *Scleroderma cruentatum* Kun L. Yang, Jia Y. Lin, Zhen-Chao Liu & Zhu L. Yang, sp. nov. (Figure 23 and Table 10).

Registration identifier: FN573005.

Etymology: Referring to the bloody coloration of damaged basidiomata.

Type: China. Guangdong Province: Guangzhou City, Baiyun District, Baiyun Mountain Scenic Area, 23°12′26″ N, 113°18′04″ E, elevation 100 m, 7 June 2025, Jia Y. Lin & Kun L. Yang, L25382 (HKAS150765, holotype; HTBM2664, isotype).

Diagnosis: Differing from other Asian species of *Scleroderma* by the pale grayish basidiomata with a distinct reddening coloration after damage.

Description: Basidiomata small, 27–51 mm in diameter, 21–35 mm in height, oblately subglobose, sessile; odor fungal; taste unknown. Peridium leathery, 2–3 mm thick, pure white (#FFFFFF) to ceramic white (#FEFEFA), distinctly turning reddish and finally blackish after damage, with thin, flaky, cottonseed gray (#BEC0BB), ash brown (#C8BFB0) to dull beaver brown (#92745E) squamules. Gleba compact at first, becoming pulverulent, strong gray (#9D9D9D). Basal rhizomorphs moderately abundant, whitish. Basidiospores {40/2/2} 7–8 (8.5) [7.51 ± 0.44, 7.50] µm in diameter including echinulate to subreticulate ornamentation 0.5–1.5 µm high, mostly globose, rarely subglobose, thick-walled, brownish, often surrounded by remnants of placental cells. Basidia collapsed. Gleba trama composed of 2–4.5 μm wide, thin-walled to slightly thick-walled, nearly colorless to slightly brownish, compact, moderately to frequently branching, subregularly arranged to interwoven hyphae with clamp connections. Peridial squamules composed of 2–5 μm wide, slightly thick-walled to thick-walled, brownish to grayish, compact, moderately branching, regularly to subregularly arranged hyphae with clamp connections. Peridial trama composed of 3–29 μm wide, slightly thick-walled, nearly colorless, compact, moderately to frequently branching, subregularly arranged to interwoven hyphae or inflated cells with clamp connections.

Habit and distribution: Solitary or gregarious in tropical to subtropical forests, associated with *Acacia mangium* (Fabaceae) and *Lithocarpus* spp. (Fagaceae). Currently known from South China and possibly also Malaysia (GenBank accession no. KT123336 (uncultured *Scleroderma* clone OTU_4724)).

Other collections examined: China. Guangdong Province: Guangzhou City, Baiyun District, Baiyun Mountain Scenic Area, 23°12′26″ N, 113°18′04″ E, elevation 100 m, 7 June 2025, Jia Y. Lin & Kun L. Yang, L25381 (HTBM2663). Guangdong Province: Guangzhou City, Huangpu District, Jiangdong Village, Boluoshan Hill, 23°11′36″ N, 113°32′25″ E, elevation 150 m, 22 June 2025, Zhen-Chao Liu, Jia Y. Lin & Kun L. Yang, S25034 (HTBM3023).

Notes: This species possibly has also been found in Yunnan Province in Southwest China (GenBank accession no. ON794285 [126]). Sequences (Table 3) support its placement as new in *Scleroderma* sect. *Sclerangium* under the frame of Yang et al. (2025) [127] collaborated with morphology.

**Figure 24:**
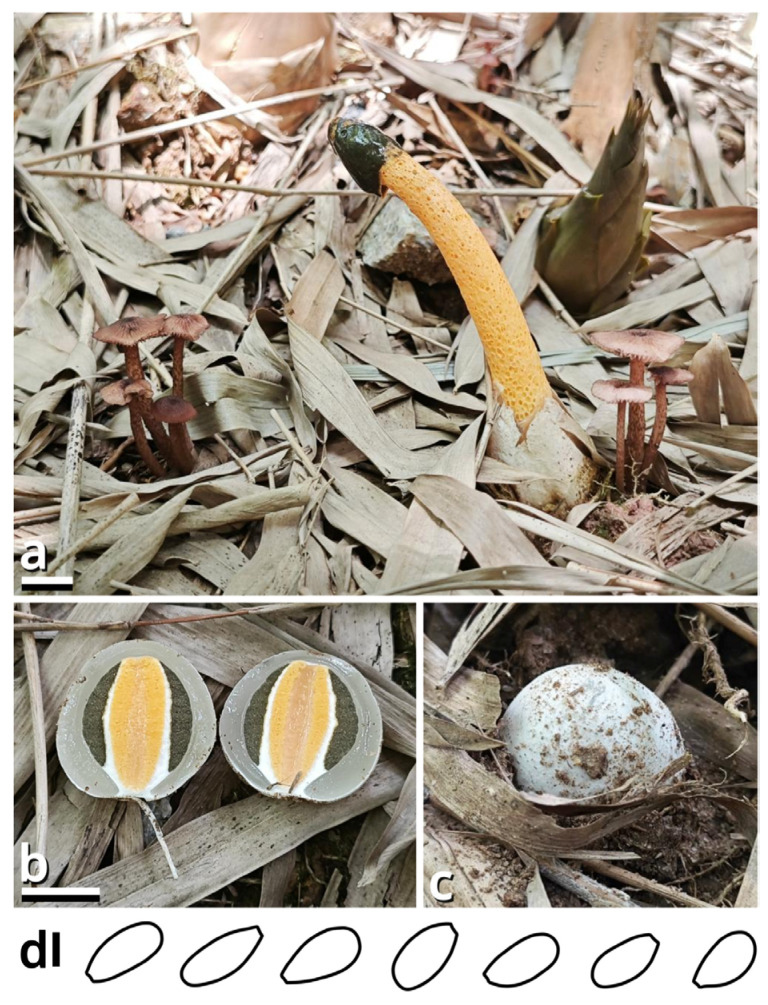
*Satyrus qiandenghuensis* (photos by Jia Y. Lin & Kun L. Yang; drawings by Kun L. Yang from HKAS150764 (holotype)). (**a**–**c**) Basidiomata ((**a**) HKAS150764 (holotype), co-occurring with *Rufolamptera profundibambusae*; (**b**,**c**) HTBM2237). (**d**) Basidiospores. Bars: (**a**,**b**) = 1 cm, (d) = 2 µm.

(1.2.1.3) Phallales E. Fisch.

(1.2.1.3.1) Phallaceae Corda.

(1.2.1.3.1.1) *Satyrus* Bosc.

(1.2.1.3.1.1.1) *Satyrus qiandenghuensis* Kun L. Yang, Jia Y. Lin & Zhu L. Yang, sp. nov. (Figure 24 and Table 10).

Registration identifier: FN573006.

Etymology: Referring to the type locality.

Type: China. Guangdong Province: Foshan City, Nanhai District, Qiandenghu Lake Park, 23°03′18″ N, 113°08′26″ E, elevation 1 m, 26 August 2024, Jia Y. Lin & Kun L. Yang, L24251 (HKAS150764, holotype; HTBM2236, isotype).

Diagnosis: Differing from other species of *Satyrus* by the fainter odor (easily becoming pleasant as gleba becomes thinner), orangish and smaller basidiomata, basidiospores sized 4–4.5 × 2–2.5 µm and the known distribution is limited in East Asia.

Description: Basidiomata medium-sized, each issued by a single rhizomorph; odor slightly rancid in thick gleba, while pleasant like syrups in thin gleba; taste unknown. Pileus (receptacle) 10–13 mm in diameter, 19–24 mm in height, conical, campanulate to paraboloid, slightly rugulose to verrucose, butter orange (#F2DF8F), bread orange (#F3C374) to honey orange (#FFAC2A), with a truncated apex, covered with gelatinized, dark swamp green (#505849) gleba. Stipe (pseudostipe) spongy, 65–78 mm long, 8–11 mm thick, tapering upwards, concolorous with the pileus in the upper part, slightly becoming lighter downwards; volval remnants membranous, with free limb up to 17 mm high, ceramic white (#FEFEFA) to merino white (#F9F5EC). Basidiospores {40/2/2} 4–4.5 [4.28 ± 0.25, 4.50] × 2–2.5 [2.24 ± 0.25, 2.00] µm, *Q* = 1.60–2.25 [1.93 ± 0.17, 1.80], oblong and more or less obovoid, thin-walled, smooth, greenish to brownish. Basidia not observed. Stipe (pseudostipe) composed of a textura angularis-globulosa by inflated cells sized 13–46 µm in diameter, slightly thick-walled, very slightly yellowish to orangish. Volval remnants around stipe (pseudostipe) base radially subregular, gelatinized, composed of filamentous hyphae 1.5–10 µm wide, thin-walled to slightly thick-walled, more or less encrusted, nearly colorless, compact, moderately to frequently branching. Clamp connections abundant.

Habit and distribution: Gregarious, on ground in tropical to subtropical bamboo forests. Currently known from South China and possibly also Pakistan (GenBank accession no. MT949887).

Other collections examined: China. Guangdong Province: Foshan City, Nanhai District, Qiandenghu Lake Park, 23°03′18″ N, 113°08′26″ E, elevation 1 m, 27 August 2024, Jia Y. Lin, L24252 (HTBM2237) and L24253 (HTBM2238).

Notes: The genus *Satyrus* had been synonymized with *Phallus* for a long time but is recently supported as independent in phylogeny, comprising two species characterized by reddish basidiomata with a rugose pileus surface, namely, *S. rubicundus* and *S. rugulosus* [5]. Sequences (Table 3) support our collections as an orangish new species of *Satyrus*, sister to *S. rubicundus* with 81% MLB in a three-locus (ITS-nrLSU-*atp6*) phylogeny based on alignment in Yang et al. (2025) [5].

(1.2.1.3.1.1.2) *Satyrus aurantiacus* (Mont.) Kun L. Yang, Jia Y. Lin & Zhu L. Yang, comb. nov.

Registration identifier: FN573034.

Basionym: *Phallus aurantiacus* Mont., *Annales des Sciences Naturelles, Botanique, Sér. 2* 16: 277 (1841).

Notes: This species described from India without available molecular data is similar to *Satyrus qiandenghuensis* but possesses more robust basidiomata. Generally it fits the circumscription of genus *Satyrus* better than *Phallus* s. str. [5] and thus is transferred.

(1.2.1.4) Polyporales Gäum.

(1.2.1.4.1) Polyporaceae Fr. ex Corda.

(1.2.1.4.1.1) *Daedaleopsis* J. Schröt.

(1.2.1.4.1.1.1) *Daedaleopsis glabra* (Lév.) Kun L. Yang, Jia Y. Lin & Zhu L. Yang, comb. nov.

Registration identifier: FN572625.

Basionym: *Hexagonia glabra* Lév. (as “*Hexagona*”), *Annales des Sciences Naturelles, Botanique, Sér. 3* 5: 143 (1846).

Collections examined: China. Guangdong Province: Guangzhou City, 31 March–20 April 2023, Jia Y. Lin & Kun L. Yang, K23006 (HTBM0521), K23055 (HTBM0570), K23094 (HTBM0609), K23135 (HTBM0650) and K23136 (HTBM0651).

**Figure 25:**
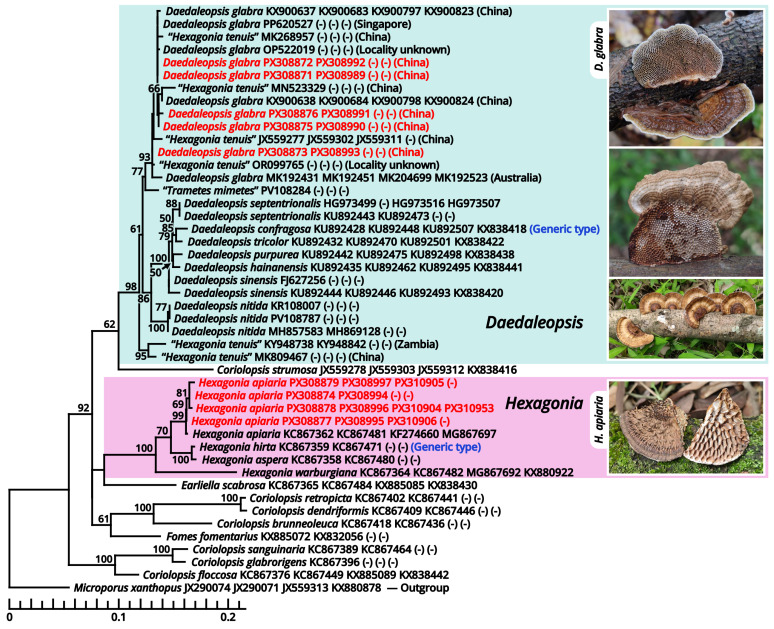
Phylogeny focusing on *Daedaleopsis*, *Hexagonia* and their allies based on four loci (ITS-nrLSU-*rpb2*-*tef-1α*), rooted with *Microporus* as outgroup (referred to Cui et al. (2019) [129]). Nodes are annotated if supported by ≥50% MLB. Collections sequenced in this study are highlighted in red. Localities for collections labeled as “*Daedaleopsis glabra*” or “*Hexagonia tenuis*” are also indicated. Photos by Kun L. Yang & Jia Y. Lin.

Notes: This species described from India is very common in South China. In our present phylogeny (Figure 25), its collections are distant from the type species of *Hexagonia* but nest in the clade recognized as *Daedaleopsis*. Generally, it also fits the morphological concept of *Daedaleopsis* emended by Cui et al. (2019) [128,129]. The transfer is thus made.

Collections labeled as “*Hexagonia tenuis*” group into two parts in the clade recognized as *Daedaleopsis* in our present phylogeny (Figure 25), with one part mixed with *Daedaleopsis glabra* and the other part isolated. It seems similar to *Daedaleopsis glabra* but also seems not originally described from Asia [130,131], and thus at least the former part is recognized as a misidentification of *D. glabra* in this study.

## 4. Discussion

### 4.1. Grazing Significantly Increases Within-Habitat Diversity of Macrofungi in Subtropical Forests

Within-habitat diversity, viz. alpha-diversity, represents species diversity in a locally uniform habitat. This study found that macrofungal species number in grazed forests was significantly higher than in ungrazed forests (70 vs. 33) and that diversity and richness indices generally increased after grazing (Figure 2 and Figure 3; Table 4), indicating that grazing significantly increases the within-habitat diversity of macrofungi in these subtropical forests. This phenomenon was correlated with the increase in large plant remains (Figure 5), suggesting that the large woody resources created by livestock foraging may contribute numerous additional niches, evidenced by the dominance of wood saprotrophs in grazed forests (Table 7). This phenomenon was also associated with the decrease in litterfall (Figure 5), suggesting that the exposed ground created by livestock trampling and excretion may also contribute more suitable habitats for soil saprotrophs, evidenced by the general increase in these fungi in grazed forests (Table 7). All forest types were dominated by few monopolistic taxa at both species and order levels before grazing, but after grazing, these dominant groups were generally weakened, with dominance becoming more evenly distributed among various taxa, suggesting that grazing shifted the community structure from one dominated by few specialized species adapted to stable environments to one shared by more species adapted to disturbed environments. With the increase in within-habitat diversity, the productivity, resistance and resilience of grazed forests may also be enhanced.

### 4.2. Grazing May Reduce Between-Habitat Diversity of Macrofungi in Subtropical Forests

Between-habitat diversity, viz. beta-diversity, represents species diversity across a variety of habitats in broader regions. This study found that all forest types contained only cosmopolitan and pantropical species before grazing while introducing species of other geographic components (and often accompanied by a dominance increase in cosmopolitan components and a dominance decrease in pantropical components) after grazing (Table 10). Since pantropical components represent endemic species of the studied ecosystems, this phenomenon suggests that the observed increase in within-habitat diversity may partly result from flora homogenization, which filters out endemic species and filters in widespread species, leading to increased similarity in fungal communities across different regions and thus potentially causing a loss of between-habitat diversity. However, unexpectedly, the grazed environment in dense-tree plantations resulted in a decrease in the dominance of cosmopolitan components and an increase in the dominance of pantropical components. This may be due to some unique microenvironments created by grazing in such forests that favor the development of endemic species (see also Section 4.4).

### 4.3. Grazing Drives Functional Group Succession of Macrofungi in Subtropical Forests

Analysis of dominant trophic types (Table 7) revealed that grazing activities shifted the ecosystem functions of macrofungal communities from symbiosis-dominated to decomposition-dominated. This further implies that the observed increase in within-habitat diversity is biased, with an increase in decomposition functional diversity at the expense of symbiotic functional diversity. As a result, grazed forests may exhibit higher material cycling efficiency but may lose some ecological services dependent on symbiosis.

### 4.4. Differential Responses to Grazing Across Forest Types

Analyses of multi-level data, including characterization indices of macrofungal diversity, dominant species, orders, trophic types and edibility types, consistently demonstrated that secondary mixed forests exhibited stronger resistance to grazing disturbance (with minor changes in data), with the two plantation types being weaker (with larger changes in data). This suggests that the natural forest ecosystem possesses a greater capacity for buffering and maintaining stability. Apart from general patterns, there are still some special responses noticed but unresolved in this study (as mentioned in Section 4.2) requiring further studies to explain.

### 4.5. The Role of Fungal Taxonomy in Ecological Studies

This study heavily relies on taxonomic knowledge, as accurate macrofungal identification is one of the fundamental prerequisites for deriving reliable ecological analyses. However, we found that a considerable number of macrofungal taxa in the experimental site lack correct names in recent taxonomic frameworks, with the following instances: (1) some commonly encountered whitish little agarics, previously identified as *Marasmiellus* spp. in the local literature [132,133], were actually found to be members of two different genera, namely, *Paramarasmius palmivorus* and *Stygiomarasmius scandens*, through molecular approaches (Figure 2 and Figure 11); (2) many grayish mycenoid agarics not documented in the local literature, frequently observed in ungrazed dense-tree plantations, were revealed as the recently known “*Mycena amicta*”, representing a large cryptic species complex that should be transferred to the genus *Basidopus*; (3) sequencing of selected collections indicated that the so-called *Graphis scripta*, which dominated across all forest types in ungrazed conditions, similarly constituted several cryptic species analogous to “*Mycena amicta*”. For all these taxa that require updates and revisions directly or indirectly related to taxa in the experimental site, we have addressed those feasible to enhance the accuracy of ecological interpretations and to improve the fungal taxonomic system. However, there were still many cases that could not be resolved due to insufficient collections or data and thus were temporarily retained under morphological concepts. This situation highlights the urgent need for mycologists and lichenologists to refine the taxonomic framework of especially the commonly encountered taxa to meet the demands of other related fields. Otherwise, practitioners in other fields may easily encounter errors when relying on such knowledge.

## Figures and Tables

**Table 1:** Types and notes of variables of interest.

Variables	Divisions	Notes
Species	-	Identified via morphology and/or molecular phylogeny
Individual number *	1	1 sporocarp; conspecific sporocarp(s) > 2 m away this sporocarp counted as another individual
	2	Group of >1 sporocarps, with adjacent sporocarps inside this group ≤ 0.5 m in distance; conspecific sporocarp(s) > 2 m away this group counted as another individual
	3	Group of >1 sporocarps, with adjacent sporocarps inside this group ≤ 1 m in distance; conspecific sporocarp(s) > 2 m away this group counted as another individual
	4	Group of >1 sporocarps, with adjacent sporocarps inside this group ≤ 2 m in distance; conspecific sporocarp(s) > 2 m away this group counted as another individual
Trophic types	Soil saprotrophs	Fungi utilizing soil (including feces) as primary nutrients
	Wood saprotrophs	Fungi utilizing dead trees, branches and leaves as primary nutrients
	Alga symbionts	Fungi forming close symbiotic (including parasitic) relationships with cyanobacteria and/or eukaryotic algae
	Plant symbionts	Fungi forming close symbiotic (including parasitic) relationships with plants
	Insect symbionts	Fungi forming close symbiotic (including parasitic) relationships with insects
	Fungus symbionts	Fungi forming close symbiotic (including parasitic) relationships with other fungi
Attachment types	Soil-inhabiting	Sporocarps mainly formed on soil (including feces)
	Wood-inhabiting	Sporocarps mainly formed on wood
Sporocarp types	Agaricoid fungi	More or less stipito-pileate and fleshy basidiomycetes with more or less lamellate hymenophore
	Boletoid fungi	More or less stipito-pileate and fleshy basidiomycetes with more or less poroid hymenophore
	Polyporoid fungi	Effused, dimidiate to stipito-pileate and more or less woody basidiomycetes with smooth, lamellate to poroid hymenophore
	Cantharelloid fungi	More or less trumpet-like and fleshy basidiomycetes with smooth to lamellate hymenophore
	Clavarioid fungi	More or less columnar and fleshy basidiomycetes with smooth hymenophore
	Gastroid fungi	More or less globose and fleshy basidiomycetes with endogenous hymenophore
	Jelly fungi	More or less lobate and gelatinous basidiomycetes with smooth hymenophore
	Ascomycetes	Fungi forming sexual spores endogenous in asci
	Slime molds	Fungus-like organisms with amoeboid stages in lifecycle
Taxonomic orders	-	A higher rank in taxonomic system reflecting an earlier level in evolutionary history, determined with recent phylogenetic studies of the corresponding group
Geographic components	Cosmopolitan	Distribution center indistinct
	North temperate	Distribution center in northern temperate zone, occasionally extending to southern temperate zone
	Pantropical	Distribution center in tropical and subtropical regions, occasionally reaching temperate zones
	East Asian	Distribution center in East Asia
Edibility types	Edible	Edible or both edible and medicinal for most people
	Medicinal	Inedible (usually due to the hard texture) but medicinal for most people
	Poisonous	Poisonous for most people
	Unknown	Edibility currently undetermined
Litterfall thickness	Undecomposed layer thickness	Thickness of fresh to slightly decomposed litterfall with recognizable forms
	Semi-decomposed layer thickness	Thickness of partly decomposed and loosely structured litterfall still with organic morphology
	Decomposed layer thickness	Thickness of highly decomposed and amorphous materials mixed with soil
Abundance of large plant remains	1	Almost none, not impeding free movement
	2	Sparse, not impeding free movement
	3	Common, impeding movement in dense areas
	4	Abundant, significantly impeding movement

* Since the individuals of macrofungi are usually vegetative bodies in substrates that we are unable to observe, we estimated individual number based on sporocarp counts.

**Table 2:** Primer pairs and specific PCR settings for each locus.

Loci	Primer Pairs	Denaturation	Annealing	Elongation	References
ITS	ITS1-F/ITS4	94 °C, 30 s	53 °C, 40 s	72 °C, 60 s	[47,48]
nrLSU	LR0R/LR5	94 °C, 30 s	53 °C, 40 s	72 °C, 90 s	[49]
*rpb1*	gRPB1-Af/fRPB1-Cr	94 °C, 60 s	52 °C, 60 s	72 °C, 80 s	[50]
*rpb2*	brpb2-6F/brpb2-7.1R	94 °C, 60 s	52 °C, 60 s	72 °C, 80 s	[51]
*tef-1α*	EF1-983F/EF1-1567R	94 °C, 30 s	53 °C, 40 s	72 °C, 60 s	[52]
*β-tub*	B36f/B12r	94 °C, 60 s	53 °C, 60 s	72 °C, 60 s	[53]
*atp6*	ATP6-1/ATP6-2	94 °C, 60 s	52 °C, 60 s	72 °C, 80 s	[54]

**Table 3:** Generated sequences for taxonomic studies. Unavailable items are indicated with -. Holotypes and epitypes are indicated by HT and ET, respectively.

Species	Collections	ITS	nrLSU	*rpb1*	*rpb2*	*tef-1α*	*β-tub*	*atp6*
*Amparoina heteracantha*	HTBM2964	PX308916	-	-	-	-	-	-
*Astraeus maculatus*	HKAS150766 (ET)	PX308869	PX308986	-	PX310901	PX310950	-	-
*Astraeus maculatus*	HTBM3131	PX308870	PX308987	-	PX310902	PX310951	-	-
*Astraeus ryoocheoninii*	HTBM2842	-	PX308988	-	PX310903	PX310952	-	-
*Astraeus ryoocheoninii*	HTBM3127	-	PX308983	-	PX310898	PX310947	-	-
*Astraeus ryoocheoninii*	HTBM3128	-	PX308984	-	PX310899	PX310948	-	-
*Astraeus ryoocheoninii*	HTBM3129	PX308868	PX308985	-	PX310900	PX310949	-	-
*Candolleomyces striginus*	HKAS150762 (HT)	PX308934	PX309050	-	-	PX310961	PX310998	-
*Candolleomyces striginus*	HTBM0800	PV155166	PV147343	-	-	PV156689	PV156727	-
*Candolleomyces striginus*	HTBM1399	PV155163	PV147340	-	-	PV156687	PV156724	-
*Candolleomyces striginus*	HTBM1548	PV155160	PV147337	-	-	PV156684	PV156722	-
*Candolleomyces vagabundoides*	HKAS150768 (HT)	PX308889	PX309007	-	-	PX310954	PX310997	-
*Chaetocalathus galeatus*	HTBM0583	PX308914	PX309023	-	-	-	-	-
*Collybiopsis clavicystidiata*	HTBM1311	PX308926	PX309046	-	-	-	-	-
*Collybiopsis gibbosa*	HTBM0911	PX308928	PX309042	-	-	-	-	-
*Collybiopsis indocta*	HTBM0739	PX308904	PX309025	-	-	-	-	-
*Collybiopsis melanopus*	HTBM1055	PX308932	PX309048	-	-	-	-	-
*Collybiopsis menehune*	HTBM0761	PX308905	PX309026	-	-	-	-	-
*Collybiopsis silvopastoralis*	HKAS150758 (HT)	PX308917	PX309035	-	-	-	-	-
*Collybiopsis silvopastoralis*	HTBM3041	PX308918	PX309036	-	-	-	-	-
*Collybiopsis subnuda*	HTBM1094	PX308909	PX309029	-	-	-	-	-
*Daedaleopsis glabra*	HTBM0521	PX308871	PX308989	-	-	-	-	-
*Daedaleopsis glabra*	HTBM0570	PX308875	PX308990	-	-	-	-	-
*Daedaleopsis glabra*	HTBM0609	PX308876	PX308991	-	-	-	-	-
*Daedaleopsis glabra*	HTBM0650	PX308872	PX308992	-	-	-	-	-
*Daedaleopsis glabra*	HTBM0651	PX308873	PX308993	-	-	-	-	-
*Gerronema angustum*	HTBM1508	PX308933	PX309049	-	-	-	-	-
*Gerronema kuruvense*	HTBM3056	PX308920	PX309038	-	-	-	-	-
*Gerronema microcarpum*	HTBM3057	PX308921	PX309039	-	-	-	-	-
*Gerronema pubescence*	HTBM1200	PX308924	PX309044	-	-	-	-	-
*Gugumyces columbarius*	HKAS150763 (HT)	PX308864	PX308981	-	-	-	-	-
*Gugumyces columbarius*	HTBM2032	PX308861	PX308978	-	-	-	-	-
*Gugumyces columbarius*	HTBM2166	PX308862	PX308979	-	-	-	-	-
*Gugumyces columbarius*	HTBM1028	PX308863	PX308980	-	-	-	-	-
*Gugumyces columbarius*	HTBM1559	PX308865	PX308982	-	-	-	-	-
*Hexagonia apiaria*	HTBM1180	PX308877	PX308995	-	PX310906	-	-	-
*Hexagonia apiaria*	HTBM2346	PX308878	PX308996	-	PX310904	PX310953	-	-
*Hexagonia apiaria*	HTBM2754	PX308879	PX308997	-	PX310905	-	-	-
*Hexagonia apiaria*	HTBM0779	PX308874	PX308994	-	-	-	-	-
*Leucoinocybe parviauricoma*	HKAS150759 (HT)	PX308922	PX309040	-	-	-	-	-
*Limacella yuexiuensis*	HKAS150756 (HT)	PX308890	PX309008	-	PX310910	PX310955	-	-
*Limacella yuexiuensis*	HTBM1240	PX308891	PX309009	-	-	-	-	-
*Marasmiellomycena entolomatoides*	HTBM2425	PX308930	PX309047	-	-	-	-	-
*Marasmius maximus*	HTBM2533	PX308931	-	-	-	-	-	-
*Neonothopanus nambi*	HTBM0678	PX308971	PX309024	-	-	-	-	-
*Oudemansiella raphanipes*	HTBM2129	PX308929	-	-	-	-	-	-
*Paramarasmius palmivorus*	HTBM0876	PX308927	PX309041	-	-	-	-	-
*Paramarasmius palmivorus*	HTBM1109	PX308923	PX309043	-	-	-	-	-
*Paramarasmius palmivorus*	HTBM1293	PX308925	PX309045	-	-	-	-	-
*Paramarasmius palmivorus*	HTBM1226	PX308910	PX309030	-	-	-	-	-
*Paramarasmius palmivorus*	HTBM1233	PX308911	PX309031	-	-	-	-	-
*Paramarasmius palmivorus*	HTBM1234	PX308912	PX309032	-	-	-	-	-
*Paramarasmius palmivorus*	HTBM3052	PX308919	PX309037	-	-	-	-	-
*Purpureodiscus masticophilus*	HKAS150755 (HT)	PX308961	PX309081	-	PX310942	PX310993	-	-
*Purpureodiscus masticophilus*	HTBM2874	-	PX309077	PX310890	PX310938	PX310988	-	-
*Purpureodiscus masticophilus*	HTBM2073	PX308966	PX309086	-	-	-	-	-
*Purpureodiscus masticophilus*	HTBM2447	PX308960	-	PX310893	-	PX310992	-	-
*Purpureodiscus masticophilus*	HTBM2449	PX308962	PX309082	PX310894	PX310943	PX310994	-	-
*Purpureodiscus masticophilus*	HTBM2877	PX308957	PX309078	-	PX310939	PX310989	-	-
*Purpureodiscus masticophilus*	HTBM2878	PX308958	PX309079	PX310891	PX310940	PX310990	-	-
*Purpureodiscus masticophilus*	HTBM2879	PX308959	PX309080	PX310892	PX310941	PX310991	-	-
*Purpureodiscus masticophilus*	HTBM2489	PX308963	PX309083	PX310895	PX310944	PX310995	-	-
*Purpureodiscus masticophilus*	HTBM2490	PX308964	PX309084	PX310896	PX310945	PX310996	-	-
*Purpureodiscus masticophilus*	HTBM2491	PX308965	PX309085	PX310897	PX310946	-	-	-
*Rufolamptera noctilucens*	HTBM2224	PX308903	PX309022	-	-	PX310960	-	-
*Rufolamptera noctilucens*	HTBM2623	PX308899	-	-	-	PX310959	-	-
*Rufolamptera profundibambusae*	HKAS150760 (HT)	PX308901	PX309017	-	PX310912	PX310957	-	-
*Rufolamptera profundibambusae*	HTBM0921	PX308900	PX309018	-	-	-	-	-
*Rufolamptera profundibambusae*	HTBM2740	PX308902	PX309021	-	PX310913	PX310958	-	-
*Rufolamptera profundibambusae*	HTBM2233	-	PX309019	-	-	-	-	-
*Rufolamptera profundibambusae*	HTBM2235	-	PX309020	-	-	-	-	-
*Satyrus qiandenghuensis*	HKAS150764 (HT)	PX308880	PX308998	-	-	-	-	-
*Satyrus qiandenghuensis*	HTBM2237	PX308881	PX308999	-	-	-	-	PX310999
*Satyrus qiandenghuensis*	HTBM2238	PX308882	PX309000	-	-	-	-	PX311000
*Schizophyllum commune*	HTBM2108	PX308915	PX309034	-	PX310911	PX310956	-	-
*Scleroderma australe*	HTBM2219	PX308867	-	-	-	-	-	-
*Scleroderma australe*	HTBM2569	PX308866	-	-	-	-	-	-
*Scleroderma cruentatum*	HKAS150765 (HT)	PX308883	PX309001	-	-	-	-	-
*Scleroderma cruentatum*	HTBM2663	PX308885	PX309003	-	-	-	-	-
*Scleroderma cruentatum*	HTBM3023	PX308884	PX309002	-	-	-	-	-
*Stygiomarasmius scandens*	HTBM0776	PX308907	PX309028	-	-	-	-	-
*Stygiomarasmius scandens*	HTBM0778	PX308908	-	-	-	-	-	-
*Stygiomarasmius scandens*	HTBM1236	PX308913	PX309033	-	-	-	-	-
*Tetrapyrgos parvispora*	HTBM0766	PX308906	PX309027	-	-	-	-	-
*Trichoderma grossum*	HTBM3134A	PX415258	-	-	-	-	-	-
*Trichoderma grossum*	HTBM3134B	PX415259	-	-	-	-	-	-
*Tortoperdon suspectum*	HKAS150757 (HT)	PX308896	PX309010	-	-	-	-	-
*Tortoperdon suspectum*	HTBM0362	PX308892	PX309012	-	-	-	-	-
*Tortoperdon suspectum*	HTBM0367	PX308893	PX309013	-	-	-	-	-
*Tortoperdon suspectum*	HTBM0368	PX308894	PX309014	-	-	-	-	-
*Tortoperdon suspectum*	HTBM0391	PX308895	PX309015	-	-	-	-	-
*Tortoperdon suspectum*	HTBM1229	PX308897	PX309011	-	-	-	-	-
*Tortoperdon suspectum*	HTBM1266	PX308898	PX309016	-	-	-	-	-
*Xanthagaricus popcorneus*	HKAS150767 (HT)	PX308888	PX309006	-	PX310909	-	-	-
*Xanthagaricus popcorneus*	HTBM2744	PX308886	PX309005	-	PX310907	-	-	-
*Xanthagaricus popcorneus*	HTBM2745	PX308887	PX309004	-	PX310908	-	-	-
*Xuaniella urbica*	HKAS150761 (HT)	PX308937	PX309053	-	PX310915	PX310964	-	-
*Xuaniella urbica*	HTBM0528	PX308935	PX309051	-	-	-	-	-
*Xuaniella urbica*	HTBM2910	PX308939	PX309056	-	PX310918	PX310967	-	-
*Xuaniella urbica*	HTBM2911	PX308940	PX309057	-	PX310919	PX310968	-	-
*Xuaniella urbica*	HTBM2536	PX308943	PX309060	-	PX310921	PX310971	-	-
*Xuaniella urbica*	HTBM2549	PX308944	PX309061	-	PX310922	PX310972	-	-
*Xuaniella urbica*	HTBM2550	PX308945	PX309062	-	PX310923	PX310973	-	-
*Xuaniella urbica*	HTBM2883	PX308936	-	-	-	PX310962	-	-
*Xuaniella urbica*	HTBM2884	PX308972	PX309052	-	PX310914	PX310963	-	-
*Xuaniella urbica*	HTBM2896	PX308973	PX309054	-	PX310916	PX310965	-	-
*Xuaniella urbica*	HTBM2897	PX308938	PX309055	-	PX310917	PX310966	-	-
*Xuaniella urbica*	HTBM1451	PX308941	PX309058	-	-	PX310969	-	-
*Xuaniella urbica*	HTBM1996	PX308942	PX309059	-	PX310920	PX310970	-	-
*Xuaniella urbica*	HTBM2586	PX308946	PX309063	-	PX310924	PX310974	-	-
*Xuaniella urbica*	HTBM2587	PX308947	PX309064	-	PX310925	PX310975	-	-
*Xuaniella urbica*	HTBM2606	PX308948	PX309065	-	PX310926	PX310976	-	-
*Xuaniella urbica*	HTBM2610	PX308949	PX309066	-	PX310927	PX310977	-	-
*Xuaniella urbica*	HTBM2612	PX308974	PX309067	-	PX310928	PX310978	-	-
*Xuaniella urbica*	HTBM2613	PX308950	PX309068	-	PX310929	PX310979	-	-
*Xuaniella urbica*	HTBM2614	PX308951	PX309069	-	PX310930	PX310980	-	-
*Xuaniella urbica*	HTBM2616	PX308975	PX309070	-	PX310931	PX310981	-	-
*Xuaniella urbica*	HTBM2686	PX308976	PX309071	-	PX310932	PX310982	-	-
*Xuaniella urbica*	HTBM2697	PX308952	PX309072	-	PX310933	PX310983	-	-
*Xuaniella urbica*	HTBM2706	PX308953	PX309073	-	PX310934	PX310984	-	-
*Xuaniella urbica*	HTBM2716	PX308954	PX309074	-	PX310935	PX310985	-	-
*Xuaniella urbica*	HTBM2719	PX308955	PX309075	-	PX310936	PX310986	-	-
*Xuaniella urbica*	HTBM2721	PX308956	PX309076	-	PX310937	PX310987	-	-

**Table 4:** Changes in characterization indices of macrofungal diversity before and after grazing. (^#^) Change after grazing compared to before grazing; (↑/|/↓) increase/no change/decrease; (*) statistically significant; (−/+/++/+++) effect negligible/minor/moderate/large.

Forest Types	Characterization Indices	Medians		*U* Values	*p* Values	Cliff’s Delta	Conclusions ^#^
		Ungrazed	Grazed				
Secondary mixed forests	Diversity index	0.570	0.680	48.0	0.173	−0.333	↑ ++
	Evenness index	0.820	0.930	30.0	0.016	−0.583	↑ * +++
	Richness index	1.140	1.345	56.5	0.386	−0.215	↑ +
Dense-tree plantations	Diversity index	0.595	0.790	0	0	−1.000	↑ * +++
	Evenness index	0.955	0.920	47.5	0.163	−0.340	↓ ++
	Richness index	0.960	1.835	0	0	−1.000	↑ * +++
Sparse-tree plantations	Diversity index	0.440	0.705	13.5	0.001	−0.813	↑ * +++
	Evenness index	0.920	0.915	71.5	1.000	−0.007	| −
	Richness index	0.910	1.315	22.5	0.005	−0.688	↑ * +++
General	Diversity index	0.560	0.755	175.5	0	−0.729	↑ * +++
	Evenness index	0.920	0.920	533.5	0.197	−0.177	↑ +
	Richness index	0.960	1.600	241.0	0	−0.628	↑ * +++

**Table 5:** Changes in top three dominant species of macrofungi before and after grazing.

Forest Types	Treatments	Nos.	Species	Dominance Index
Secondary mixed forests	Ungrazed	1	*Graphis scripta*	40.54%
		2	*Truncospora ochroleuca*	19.28%
		3	*Neofomitella guangxiensis*	9.02%
	Grazed	1	*Graphis scripta*	33.12%
		2	*Truncospora ochroleuca*	17.12%
		3	*Neofomitella guangxiensis*	9.18%
Dense-tree plantations	Ungrazed	1	*Graphis scripta*	43.20%
		2	*Coprinopsis urticicola*	10.33%
		3	*Basidopus amictus*	7.75%
	Grazed	1	*Graphis scripta*	20.36%
		2	*Sanguinoderma rugosum*	9.23%
		3	*Schizophyllum commune*	7.54%
Sparse-tree plantations	Ungrazed	1	*Graphis scripta*	51.14%
		2	*Sulzbacheromyces sinensis*	24.43%
		3	*Scortechinia diminuspora*	3.23%
	Grazed	1	*Graphis scripta*	27.18%
		2	*Sulzbacheromyces sinensis*	8.40%
		3	*Auricularia cornea*	7.51%
General	Ungrazed	1	*Graphis scripta*	44.08%
		2	*Truncospora ochroleuca*	10.49%
		3	*Sulzbacheromyces sinensis*	7.55%
	Grazed	1	*Graphis scripta*	25.81%
		2	*Truncospora ochroleuca*	7.11%
		3	*Sanguinoderma rugosum*	4.82%

**Table 6:** Changes in top three dominant orders of macrofungi before and after grazing.

Forest Types	Nos.	Ungrazed		Grazed	
		Orders	Dominance Index	Orders	Dominance Index
Secondary mixed forests	1	Ostropales	40.54%	Polyporales	40.69%
	2	Polyporales	38.84%	Ostropales	33.12%
	3	Agaricales	9.47%	Agaricales	9.05%
Dense-tree plantations	1	Ostropales	43.20%	Agaricales	38.47%
	2	Agaricales	40.75%	Polyporales	20.73%
	3	Lepidostromatales	5.72%	Ostropales	20.36%
Sparse-tree plantations	1	Ostropales	51.14%	Ostropales	27.18%
	2	Lepidostromatales	24.43%	Agaricales	26.56%
	3	Agaricales	11.49%	Polyporales	21.64%
General	1	Ostropales	44.08%	Agaricales	27.00%
	2	Polyporales	21.58%	Polyporales	26.51%
	3	Agaricales	19.25%	Ostropales	25.81%

**Table 7:** Changes in top three dominant trophic types of macrofungi before and after grazing.

Forest Types	Nos.	Ungrazed		Grazed	
		Trophic Types	Dominance Index	Trophic Types	Dominance Index
Secondary mixed forests	1	Wood saprotrophs	49.99%	Wood saprotrophs	55.36%
	2	Alga symbionts	40.54%	Alga symbionts	33.12%
	3	Plant symbionts	4.73%	Soil saprotrophs	7.54%
Dense-tree plantations	1	Alga symbionts	48.92%	Wood saprotrophs	63.19%
	2	Wood saprotrophs	35.59%	Alga symbionts	22.40%
	3	Soil saprotrophs	15.49%	Soil saprotrophs	14.41%
Sparse-tree plantations	1	Alga symbionts	75.57%	Wood saprotrophs	50.54%
	2	Wood saprotrophs	21.92%	Alga symbionts	35.58%
	3	Soil saprotrophs	2.51%	Soil saprotrophs	13.88%
General	1	Alga symbionts	51.63%	Wood saprotrophs	57.50%
	2	Wood saprotrophs	38.82%	Alga symbionts	29.09%
	3	Soil saprotrophs	6.62%	Soil saprotrophs	12.34%

**Table 8:** Dominance index for attachment types of macrofungi before and after grazing.

Forest Types	Nos.	Ungrazed		Grazed	
		Attachment Types	Dominance Index	Attachment Types	Dominance Index
Secondary mixed forests	1	Wood-inhabiting	91.90%	Wood-inhabiting	89.72%
	2	Soil-inhabiting	8.10%	Soil-inhabiting	10.28%
Dense-tree plantations	1	Wood-inhabiting	78.79%	Wood-inhabiting	83.55%
	2	Soil-inhabiting	21.21%	Soil-inhabiting	16.45%
Sparse-tree plantations	1	Wood-inhabiting	73.06%	Wood-inhabiting	77.72%
	2	Soil-inhabiting	26.94%	Soil-inhabiting	22.28%
General	1	Wood-inhabiting	83.52%	Wood-inhabiting	83.64%
	2	Soil-inhabiting	16.48%	Soil-inhabiting	16.36%

**Table 9:** Changes in top three dominant sporocarp types of macrofungi before and after grazing.

Forest Types	Nos.	Ungrazed		Grazed	
		Sporocarp Types	Dominance Index	Sporocarp Types	Dominance Index
Secondary mixed forests	1	Ascomycetes	42.22%	Polyporoid fungi	46.58%
	2	Polyporoid fungi	38.84%	Ascomycetes	33.12%
	3	Agaricoid fungi	9.47%	Agaricoid fungi	9.05%
Dense-tree plantations	1	Ascomycetes	43.20%	Agaricoid fungi	35.49%
	2	Agaricoid fungi	40.75%	Ascomycetes	33.57%
	3	Clavarioid fungi	5.72%	Polyporoid fungi	22.95%
Sparse-tree plantations	1	Ascomycetes	54.37%	Ascomycetes	34.44%
	2	Agaricoid fungi	11.49%	Agaricoid fungi	25.12%
	3	Polyporoid fungi	9.70%	Polyporoid fungi	21.64%
General	1	Ascomycetes	45.62%	Ascomycetes	42.52%
	2	Polyporoid fungi	21.58%	Polyporoid fungi	29.12%
	3	Agaricoid fungi	19.25%	Agaricoid fungi	21.10%

**Table 10:** Changes in top three dominant geographical components of macrofungi before and after grazing.

Forest Types	Nos.	Ungrazed		Grazed	
		Geographical Components	Dominance Index	Geographical Components	Dominance Index
Secondary mixed forests	1	Cosmopolitan	80.14%	Cosmopolitan	80.95%
	2	Pantropical	19.86%	Pantropical	13.16%
	3	-	-	North temperate	4.38%
Dense-tree plantations	1	Cosmopolitan	76.76%	Cosmopolitan	70.80%
	2	Pantropical	23.24%	Pantropical	27.33%
	3	-	-	North temperate/ East Asian	0.93%/0.93%
Sparse-tree plantations	1	Cosmopolitan	64.08%	Cosmopolitan	76.92%
	2	Pantropical	35.92%	Pantropical	18.76%
	3	-	-	North temperate	2.89%
General	1	Cosmopolitan	81.00%	Cosmopolitan	75.29%
	2	Pantropical	19.00%	Pantropical	19.21%
	3	-	-	North temperate	2.36%

**Table 11:** Changes in top three dominant edibility types of macrofungi before and after grazing.

Forest Types	Nos.	Ungrazed		Grazed	
		Edibility Types	Dominance Index	Edibility Types	Dominance Index
Secondary mixed forests	1	Unknown	90.22%	Unknown	90.26%
	2	Edible	5.04%	Edible	5.76%
	3	Poisonous	3.05%	Poisonous	2.47%
Dense-tree plantations	1	Unknown	94.84%	Unknown	70.57%
	2	Edible	3.14%	Edible	14.42%
	3	Medical	2.03%	Medical	10.34%
Sparse-tree plantations	1	Unknown	96.77%	Unknown	82.39%
	2	Medical	3.23%	Edible	11.84%
	3	-	-	Medical	4.03%
General	1	Unknown	93.22%	Unknown	79.35%
	2	Edible	3.24%	Edible	11.31%
	3	Medical	2.15%	Medical	6.13%

**Table 12:** Changes in environmental factors of interest before and after grazing. Litterfall layers were measured by thickness (cm); abundance of large plant remains were measured by four levels (1/2/3/4); (^#^) change after grazing compared to before grazing; (↑/|/↓) increase/no change/decrease; (*) statistically significant; (−/+/++/+++) effect negligible/minor/moderate/large.

Forest Types	Environmental Factors	Medians		*U* Values	*p* Values	Cliff’s Delta	Conclusions ^#^
		Ungrazed	Grazed				
Secondary mixed forests	Undecomposed layer	2.50	1.00	12.5	0.001	−0.826	↓ * +++
	Semi-decomposed layer	1.25	0.50	14.0	0.001	−0.806	↓ * +++
	Decomposed layer	0.50	0.25	33.0	0.016	−0.542	↓ * +++
	All litterfall layers	4.25	1.75	13.0	0.001	−0.819	↓ * +++
	Large plant remains	2.00	3.00	22.5	0.003	−0.6875	↑ * +++
Dense-tree plantations	Undecomposed layer	1.00	0.50	23.5	0.003	−0.674	↓ * +++
	Semi-decomposed layer	0.50	0.25	60.0	0.437	−0.167	↓ +
	Decomposed layer	0	0	72.0	1.000	0	| −
	All litterfall layers	1.50	0.75	32.5	0.020	−0.549	↓ * +++
	Large plant remains	2.00	4.00	10.0	0	−0.861	↑ * +++
Sparse-tree plantations	Undecomposed layer	1.00	1.00	52.0	0.203	−0.278	↓ +
	Semi-decomposed layer	0.50	0.25	66.0	0.713	−0.083	↓ −
	Decomposed layer	0	0	72.0	1.000	0	| −
	All litterfall layers	1.50	1.00	54.5	0.302	−0.243	↓ +
	Large plant remains	1.50	3.00	3.0	0	−0.958	↑ * +++
General	Undecomposed layer	1.00	1.00	298.5	0	−0.539	↓ * +++
	Semi-decomposed layer	0.50	0.50	443.0	0.012	−0.316	↓ * +
	Decomposed layer	0	0	561.0	0.179	−0.134	↓ −
	All litterfall layers	1.50	1.00	358.5	0.001	−0.447	↓ * ++
	Large plant remains	2.00	4.00	116.0	0	−0.821	↑ * +++

**Table 13:** Significant generic names either synonymized with or threatening those applied in this study.

Generic Names of Concern	Basionym of Type Species	Comments	Generic Names Applied in This Study Being Threatened by Priority
*Bactroboletus* Clem. (1909)	*Filoboletus mycenoides* Henn. (1899)	Later synonym of *Filoboletus* Henn. (1899).	-
*Corrugaria* Métrod (1949)	*Corrugaria viridiflava* Métrod (1949)	Type species unsequenced. Similar species with clear concept unrecognized.	*Amparoina* Singer (1958) *Cruentomycena* R.H. Petersen, Kovalenko & O.V. Morozova (2008) *Cynema* Maas Geest. & E. Horak (1995) *Decapitatus* Redhead & Seifert (2000) *Resinomycena* Redhead & Singer (1981) *Roridomyces* Rexer (1994) *Rufolamptera* (this study)
*Dictyoploca* Mont. ex Pat. (1890)	*Marasmius plectophyllus* Mont. (1854)	Type species unsequenced. Similar species with clear concept unrecognized.	*Amparoina* Singer (1958) *Basidopus* Earle (1909) *Collopus* Earle (1909) *Cruentomycena* R.H. Petersen, Kovalenko & O.V. Morozova (2008) *Cynema* Maas Geest. & E. Horak (1995) *Decapitatus* Redhead & Seifert (2000) *Favolaschia* (Pat.) Pat. (1892) *Filoboletus* Henn. (1899) *Galactopus* Earle (1909) *Insiticia* Earle (1909) *Linopodium* Earle (1909) *Resinomycena* Redhead & Singer (1981) *Roridomyces* Rexer (1994) *Rufolamptera* (this study)
*Eomycenella* G.F. Atk. (1902)	*Eomycenella echinocephala* G.F. Atk. (1902)	Type species unsequenced, similar to *Amparoina* Singer (1958) and *Basidopus* Earle (1909).	*Amparoina* Singer (1958) *Basidopus* Earle (1909) *Collopus* Earle (1909) *Cruentomycena* R.H. Petersen, Kovalenko & O.V. Morozova (2008) *Cynema* Maas Geest. & E. Horak (1995) *Decapitatus* Redhead & Seifert (2000) *Galactopus* Earle (1909) *Insiticia* Earle (1909) *Linopodium* Earle (1909) *Resinomycena* Redhead & Singer (1981) *Roridomyces* Rexer (1994) *Rufolamptera* (this study)
*Leiopoda* Velen. (1947)	*Leiopoda moranae* Velen. (1947)	Type species unsequenced. Similar species with clear concept unrecognized.	*Amparoina* Singer (1958) *Cruentomycena* R.H. Petersen, Kovalenko & O.V. Morozova (2008) *Cynema* Maas Geest. & E. Horak (1995) *Decapitatus* Redhead & Seifert (2000) *Resinomycena* Redhead & Singer (1981) *Roridomyces* Rexer (1994) *Rufolamptera* (this study)
*Mycenoporella* Overeem (1926)	*Mycenoporella lutea* Overeem (1926)	Type species unsequenced, similar to *Favolaschia* (Pat.) Pat. (1892) and *Filoboletus* Henn. (1899).	*Amparoina* Singer (1958) *Cruentomycena* R.H. Petersen, Kovalenko & O.V. Morozova (2008) *Cynema* Maas Geest. & E. Horak (1995) *Decapitatus* Redhead & Seifert (2000) *Resinomycena* Redhead & Singer (1981) *Roridomyces* Rexer (1994) *Rufolamptera* (this study)
*Mycenopsis* Velen. (1947)	*Mycenopsis globispora* Velen. (1947)	Type species unsequenced. Similar species with clear concept unrecognized.	*Amparoina* Singer (1958) *Cruentomycena* R.H. Petersen, Kovalenko & O.V. Morozova (2008) *Cynema* Maas Geest. & E. Horak (1995) *Decapitatus* Redhead & Seifert (2000) *Resinomycena* Redhead & Singer (1981) *Roridomyces* Rexer (1994) *Rufolamptera* (this study)
*Mycenula* P. Karst. (1889)	*Agaricus purus* Pers. (1794)	Later synonym of *Prunulus* Gray (1821).	-
*Phlebomycena* R. Heim (1966)	*Phlebomycena madecassensis* R. Heim (1966)	Type species unsequenced. Similar species with clear concept unrecognized.	*Cruentomycena* R.H. Petersen, Kovalenko & O.V. Morozova (2008) *Cynema* Maas Geest. & E. Horak (1995) *Decapitatus* Redhead & Seifert (2000) *Resinomycena* Redhead & Singer (1981) *Roridomyces* Rexer (1994) *Rufolamptera* (this study)
*Poromycena* Overeem (1926)	*Poromycena decipiens* Overeem (1926)	Type species unsequenced, similar to *Favolaschia* (Pat.) Pat. (1892) and *Filoboletus* Henn. (1899).	*Amparoina* Singer (1958) *Cruentomycena* R.H. Petersen, Kovalenko & O.V. Morozova (2008) *Cynema* Maas Geest. & E. Horak (1995) *Decapitatus* Redhead & Seifert (2000) *Resinomycena* Redhead & Singer (1981) *Roridomyces* Rexer (1994) *Rufolamptera* (this study)
*Pseudomycena* Cejp (1929)	*Agaricus tenerrimus* Berk. (1836)	Later synonym of *Basidopus* Earle (1909).	-
*Stereopodium* Earle (1909)	*Agaricus galericulatus* Scop. (1772)	Later synonym of *Mycena* (Pers.) Roussel (1806).	-

## Data Availability

The sequences generated in this study are available in GenBank (https://www.ncbi.nlm.nih.gov/genbank/, accessed on 1 July 2025) with the accession numbers shown in Table 3. The collections examined in this study were deposited in the Herbarium of Cryptogams in Kunming Institute of Botany of Chinese Academy of Sciences (KUN-HKAS) and Kun L. Yang’s private herbarium (HTBM). Supplementary Materials were deposited in Zenodo (https://zenodo.org/) via www.doi.org/10.5281/zenodo.17106910.

## Supplementary Materials

The following supporting information can be downloaded at: https://www.mdpi.com/article/10.3390/jof11100749/s1, Alignments S1: Concatenated ITS-nrLSU-*rpb2*-*tef-1α* alignment used for phylogenetic analyses of *Catatrama*, *Limacella*, *Limacellopsis* and *Zhuliangomyces*; Alignments S2: ITS alignment used for phylogenetic analyses focusing on recently accepted but ambiguous genera of Marasmiineae, Mycenineae and Schizophyllineae; Alignments S3: nrLSU alignment used for phylogenetic analyses focusing on recently accepted but ambiguous genera of Marasmiineae, Mycenineae and Schizophyllineae; Alignments S4: ITS alignment used for phylogenetic analyses focusing on recently accepted *Mycena* species within Mycenaceae; Alignments S5: ITS alignment used for phylogenetic analyses focusing on recently accepted *Mycena* species excluded from Mycenaceae in this study; Alignments S6: Concatenated ITS-nrLSU-*rpb2*-*tef-1α* alignment used for phylogenetic analyses focusing on *Daedaleopsis*, *Hexagonia* and their allies.
